# Adjusting for covariates and assessing modeling fitness in machine learning using MUVR2

**DOI:** 10.1093/bioadv/vbae051

**Published:** 2024-04-04

**Authors:** Yingxiao Yan, Tessa Schillemans, Viktor Skantze, Carl Brunius

**Affiliations:** Department of Life Sciences, Chalmers University of Technology, Gothenburg, Sweden; Cardiovascular and Nutritional Epidemiology, Institute of Environmental Medicine, Karolinska Institute, Stockholm, Sweden; Fraunhofer-Chalmers Research Centre for Industrial Mathematics, Gothenburg, Sweden; Department of Life Sciences, Chalmers University of Technology, Gothenburg, Sweden; Chalmers Mass Spectrometry Infrastructure, Chalmers University of Technology, Gothenburg SE-41296, Sweden

## Abstract

**Motivation:**

Machine learning (ML) methods are frequently used in Omics research to examine associations between molecular data and for example exposures and health conditions. ML is also used for feature selection to facilitate biological interpretation. Our previous MUVR algorithm was shown to generate predictions and variable selections at state-of-the-art performance. However, a general framework for assessing modeling fitness is still lacking. In addition, enabling to adjust for covariates is a highly desired, but largely lacking trait in ML. We aimed to address these issues in the new MUVR2 framework.

**Results:**

The MUVR2 algorithm was developed to include the regularized regression framework elastic net in addition to partial least squares and random forest modeling. Compared with other cross-validation strategies, MUVR2 consistently showed state-of-the-art performance, including variable selection, while minimizing overfitting. Testing on simulated and real-world data, we also showed that MUVR2 allows for the adjustment for covariates using elastic net modeling, but not using partial least squares or random forest.

**Availability and implementation:**

Algorithms, data, scripts, and a tutorial are open source under GPL-3 license and available in the *MUVR2* R package at https://github.com/MetaboComp/MUVR2.

## 1 Introduction

Omics technologies developed over the last decades have permitted biomedical and life science research from genes down to metabolites (Perakakis *et al.*, 2018). However, omics technologies typically measure more variables than the number of observations (Perakakis *et al.*, 2018) for which supervised machine learning (ML) is well suited (Wiemken and Kelley, 2019). Typically, ML requires fewer assumptions of the data and can natively manage interactions and collinearities among a large number of predictors, as well as circumvent multiple testing biases (Wiemken and Kelley, 2019).

However, ML methods also have concerns: Overfitting models to data exaggerates prediction performance (Hawkins, 2004). Although overfitting can be dramatically reduced by cross-validation (CV) and quantified by permutation tests (Afanador *et al.*, 2016, Yi *et al.*, 2016), a general framework for assessing modeling overfitting in ML is lacking.

With the large number of features and noise in omics datasets, selecting informative features of interest is needed for biological interpretation. This can be achieved using variable importance ranks (Afanador *et al.*, 2016, Yi *et al.*, 2016). The Shapley additive explanation (SHAP) procedure was recently shown to provide interpretable variable selection (Lundberg and Lee, 2017). However, using all available data for feature selection introduces the risk of data leakage and false discovery (Ambroise and McLachlan, 2002, Berisha *et al.*, 2021). To overcome these issues, we developed the MUVR algorithm, which performs ML modeling and variable selection through recursive elimination within a repeated double CV (rdCV) (Shi *et al.*, 2019).

The addition of covariates is common in univariate analysis to accommodate for known causal structures, for example for confounder adjustment. However, a key trait in ML approaches is that one need not anticipate such causal structures and covariate adjustment is typically not even possible (Posma *et al.*, 2018). Consequently, variables of interest identified in ML often reflect covariates, which may not be of causal interest. Adjusting for covariates already in ML modeling can thus help to filter out likely non-relevant predictors and shift the focus towards more interesting candidates, making ML modeling better suited for use in, for example epidemiological studies. Some approaches were suggested, for example counterweighting in PLS (Posma *et al.*, 2018), regularization in lasso (Tibshirani, 1996) and elastic net (EN) (De Mol *et al.*, 2009), and regression strategies (Posma *et al.*, 2018). Nevertheless, these approaches struggle with non-linearities and interactions between predictor variables and can lead to reduced predictive power. There is also a scarcity of implementations (Posma *et al.*, 2018).

Herein, we aimed to investigate the possibility of developing the MUVR framework to include additional ML methods [support vector machines (SVM), artificial neural networks (ANN), and EN] (Mendez *et al.*, 2019). Furthermore, we aimed to incorporate covariate adjustment and investigate prediction performance and overfitting across different modeling strategies. These were implemented in the new *MUVR2* package to highlight the added functionality and reduce compatibility issues for users of the old package.

## 2 Methods

### 2.1 Datasets

#### 2.1.1 Freelive2

This dataset describes metabolic profiles in relation to dietary exposures and is adapted from the MUVR Freelive dataset in the original *MUVR* package (Shi *et al.*, 2019). One thousand one hundred forty-seven urine metabolite features of 58 unique participants are used as predictors. Their reported wholegrain rye consumption is used as a continuous target variable. Detailed information is described elsewhere (Hanhineva *et al.*, 2015).

#### 2.1.2 Mosquito

The dataset describes the microbiota composition in mosquitos in relation to their villages of capture in Burkina Faso (Shi *et al.*, 2019). The predictors consist of 1678 16S operational taxonomic units (of which 738 show non-near-zero variance) and the village of capture is used as a categorical target variable. Detailed information is described elsewhere (Buck *et al.*, 2016).

#### 2.1.3 BioDiva

This dataset describes the metabolic profiles of 421 individuals who later developed type 2 diabetes and their individually matched controls from the Västerbotten Intervention Program (Shi *et al.*, 2018). Twenty-four thousand seven hundred fifty-eight metabolite features are used as predictors and future diabetes status is used as a binary categorical target variable. Additionally, information is available for covariates, including age and sex. Detailed information regarding the study design is described elsewhere (Norberg *et al.*, 2010, Shi *et al.*, 2018).

### 2.2 The original MUVR algorithm

**Figure 1.**
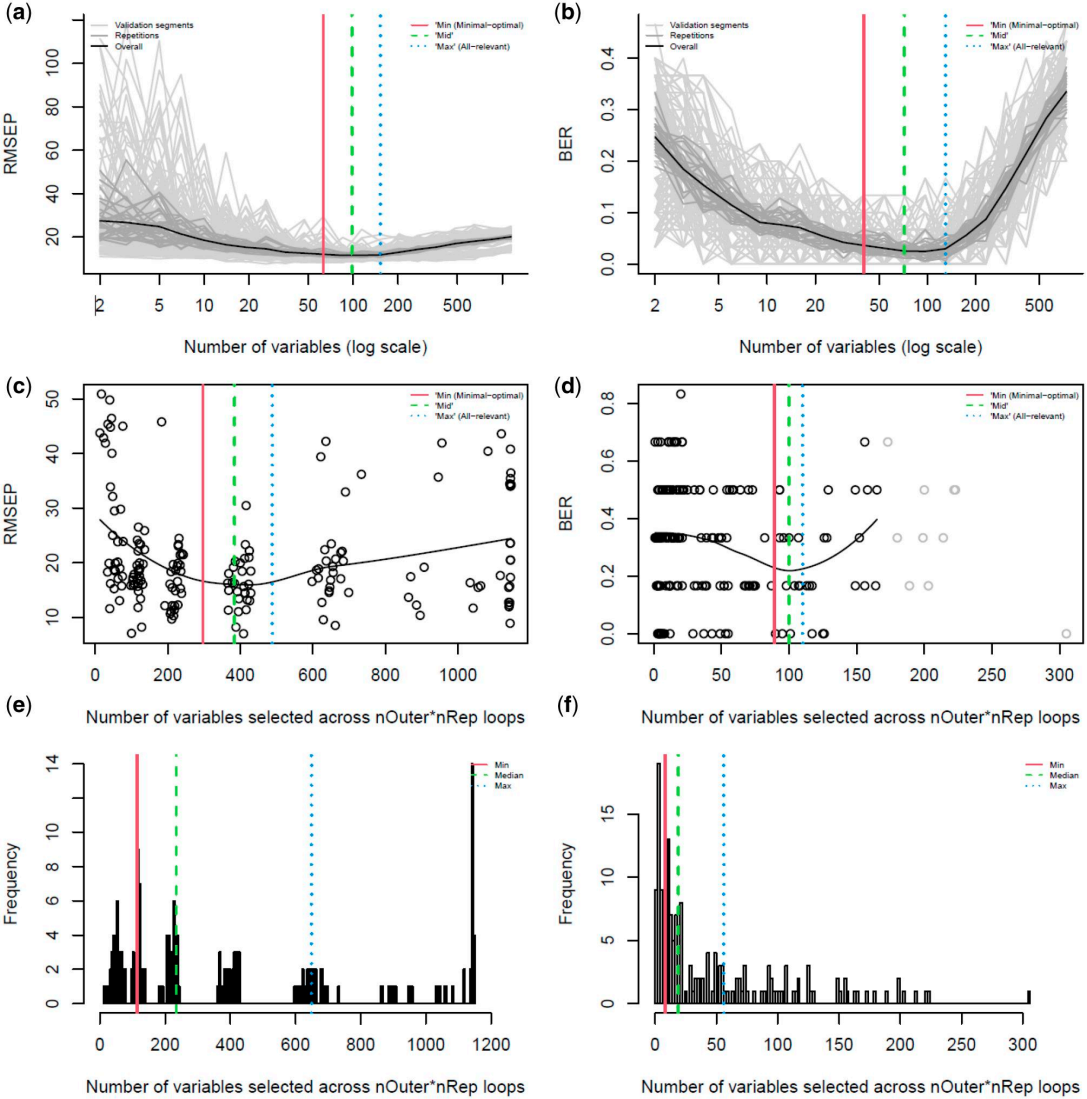
Variable selection procedures in MUVR2. The left column (**a, c, e**) represents regression (Freelive2 data). The right column (**b, d, f**) represents classification (Mosquito data). The top row (**a, b**) represents the standard variable selection exemplified for PLS, showing prediction performance as a function of the number of variables through recursive elimination and the “min,” “mid,” and “max” variable selections. The middle row (**c, d**) shows prediction performance in MUVR2-EN as a function of the number of selected variables, similar to the standard procedure above, excluding outliers (identified by the interquartile range procedure; in grey). The bottom row (**e, f**) shows the variable selection in MUVR2-EN, based directly on quantiles of selected variables, not taking modeling performance into account. All models were run using *nRep *=* *30 and *nOuter *=* *6. For PLS, *varRatio *=* *0.75.

The original MUVR algorithm is described in detail elsewhere (Shi *et al.*, 2019). In brief, PLS and RF were supported for regression and classification as well as multilevel problems, that is classification analysis of dependent samples (Szymańska *et al.*, 2012). MUVR performs rdCV (Filzmoser *et al.*, 2009) with recursive backward elimination based on variable importance ranks (Shi *et al.*, 2019). This results in four nested loops, governed by key parameters (*in parenthesis*): (i) The outmost loop performs repetitions of the overall procedure (*nRep*) to address stochastic effects from CV segmentation and obtain more stable estimates; (ii) The outer CV loop separates the entire data into testing and calibration sets (*nOuter*); (iii) A recursive variable elimination loop removes a proportion of variables (*varRatio*) ranked to have the worst variable importance in the calibration set; (iv) The inner CV loop separates the calibration set into validation and training sets (*nInner*) for hyperparameter tuning and the calculation of variable importance. Model performance for the calibration set model is assessed by root-mean-squared error of prediction in regression and balanced error rate (BER), number of misclassifications (MISS), or the area under receiver operation characteristic curve (AUROC) in classification. Final model performance is evaluated by *Q*^2^ in regression and BER, MISS, and AUROC in classification. Three consensus models with similar prediction performance but different numbers of selected variables (i.e. “min,” “mid,” “max”) are obtained. The “min” and “max” correspond to the minimal-optimal and all-relevant number of predictors (e.g. Fig. 1a and b). The “mid” model corresponds to their geometric mean and is an approximated “best” model. In addition, the *MUVR* package provides functionality for permutation tests to assess prediction performance.

### 2.3 The MUVR2 algorithm

We investigated the incorporation of SVM (Noble, 2006), ANN (Venkateswaran and Ciaburro, 2017), and EN (De Mol *et al.*, 2009). In addition, we included covariate adjustment (Section 2.4) and upgraded permutation tests into what we herein denote as resampling tests (Section 2.5), which also includes a reference distribution for assessing overfitting. For convenience, we further incorporated one-hot encoding (Yu *et al.*, 2022) of categorical variables to facilitate their use in ML analysis. This procedure entails re-coding categorical variables onto multiple numerical variables coded as 1 or 0 for class membership (Supplementary Fig. S1). MUVR2 is accompanied by a tutorial accessible at the web repository.

To investigate MUVR2-SVM, we used the *kernlab* package (Karatzoglou *et al.*, 2006) to perform SVM and the *rminer* package (Cortez, 2022) to calculate variable importance, which allowed flexible penalty tuning. For MUVR2-ANN, we investigated both the *neuralnet* (Günther and Fritsch, 2010) and *nnet* (Venkateswaran and Ciaburro, 2017) to perform ANN and both the *caret* package (Kuhn, 2008) and Olden and Garson’s algorithms provided by *NeuralnetTools* (Beck, 2018) to obtain variable importance. We applied a simple neural network with one hidden layer, where the number of nodes could be customized manually. Variable selection for MUVR2-SVM and MUVR2-ANN was performed as in MUVR2-PLS and MUVR2-RF described above.

For MUVR2-EN, we used the *glmnet* package. Calibration set models were obtained using the built-in CV function instead of through recursive elimination. However, a similarly nested CV structure was achieved by nesting the built-in CV in an outer CV loop. Variable importance was calculated per variable as the proportion of having a non-zero beta coefficient across the *nRep* × *nOuter* calibration set models. Variable selection is obtained either from assessing model performance in relation to the number of non-zero beta coefficients (Fig. 1c and d) or directly from quantiles of the distribution of non-zero beta coefficients across calibration set models (see tutorial) (Fig. 1e and f).

### 2.4 Covariate adjustment

We originally hypothesized that adjustment for covariates could be achieved by consistently forcing inclusion in the modeling (governed by the *keep* argument), that is by excluding them from recursive elimination in the standard procedure or by suppressing their regularization in MUVR2-EN. The rationale was that it would diminish the importance of covariate-associated predictors. For convenience, we refer to this procedure as *keep*ing a variable.

**Figure 2.**
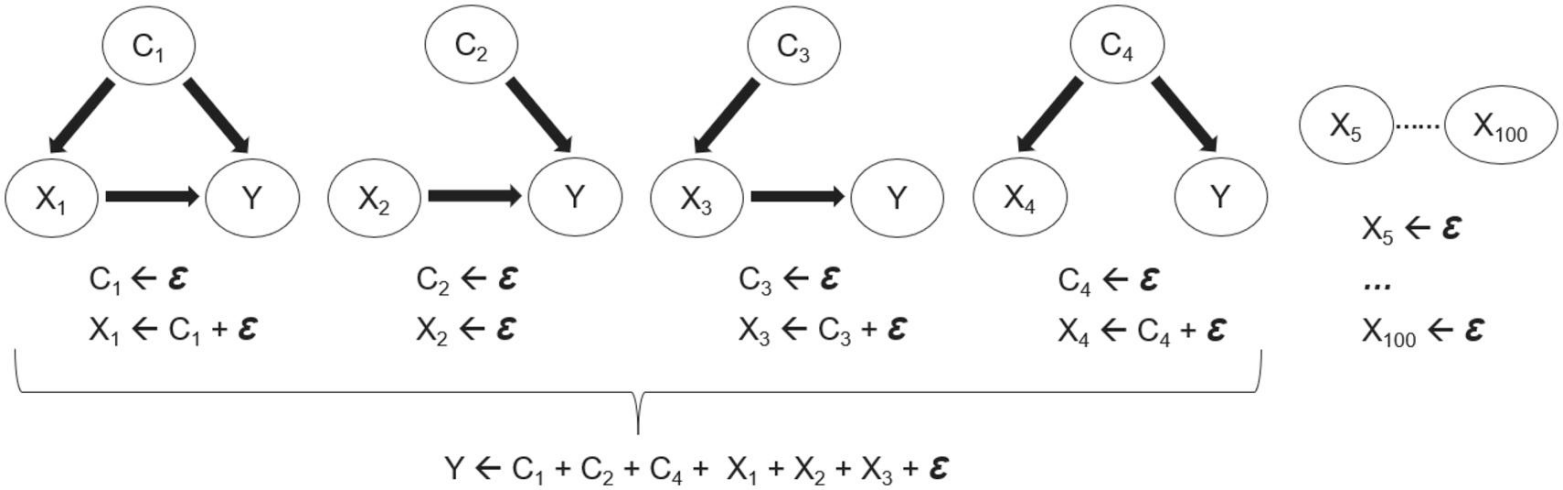
Causal structures between simulated predictors (X, *n* = 100), covariates (C, *n* = 4), and the target variable Y. Ɛ represents random noise with a standard normal distribution. Arrows indicate linear (causal) dependency. Additional information is in Supplementary Fig. S2.

We then simulated variables according to different causal structures (Fig. 2 and Supplementary Fig. S2) and observed the effects of *keep*ing covariates on the variable importance ranks of predictors of interest (i.e. X_1_, X_2_, X_3_, and X_4_ in Fig. 2). Additional details on data generation, and correlation between predictors of interests, covariates, and the target variable are available in Supplementary Fig. S2.

We then further tested the *keep* functionality in a real-world classification problem, using the BioDiva data. The functionality was assessed by comparing how many times sex-correlated features had non-zero beta coefficients in the calibration set models, when *keep*ing versus not *keep*ing sex as a potential confounder.

### 2.5 Resampling tests to assess model fitness and overfitting

Permutation tests are used to assess model performance, by comparing actual prediction performance to that using permuted target variables, that is when breaking the underlying associations between predictors and target variables (Westerhuis *et al.*, 2008, Shi *et al.*, 2019). For convenience, we refer to the prediction performance, such as *Q*^2^ for regression and BER for classification analysis, as fitness.

Here, we further elaborate on permutation tests in two areas: First, in the simulation of a null-hypothesis target variable, we have increased variability compared to standard permutations. The rationale comes from observing that model predictions are not bounded by exact values or proportions of the actual target variable. We argue that the null-hypothesis target variable should similarly not have such constraints and instead obtain it from random draws from its empirical distribution. In regression, this results in numeric values not necessarily observed in the actual target variable but representing the same underlying distribution, given enough samples. In classification, this represents sampling the target variable based on class probabilities. We refer to this new type of test as resampling tests.

**Figure 3.**
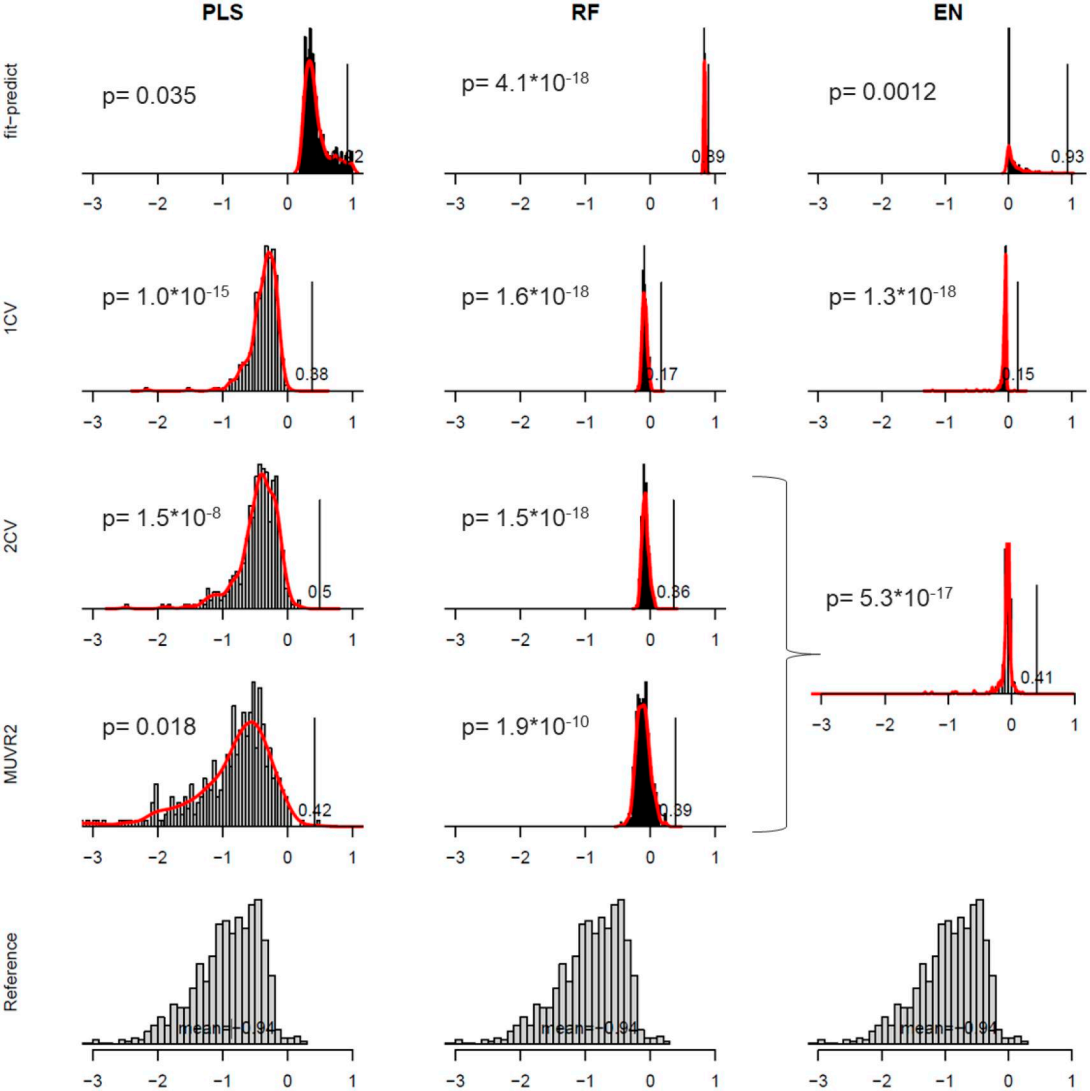
Predictive performance in regression (*Q*^2^) for actual modeling (**fitness_actual_**; vertical lines) and resampling tests **(H0_modeled_**; histograms and smoothed curves) and reference distribution from resampling the target variable without modeling (**H0_reference_**; histograms at the bottom). Modeling was performed using PLS (left), RF (middle), and EN (right) with different validation strategies, including *fit-predict*, *1CV*, *2CV*, and *MUVR2* (except for EN, since *2CV* is identical to *MUVR2*) using the Freelive2 data. *P*-values were generated from the smoothed curve of the **H0_modeled_** distribution. Partial least squares, PLS; Random forest, RF; Elastic net, EN.

Second, to further assess overfitting, we introduce a reference distribution for the null-hypothesis conditions by calculating fitness directly from the resampled target variables, instead of from any ML modeling, effectively excluding overfitting altogether. The reference distribution represents a natural scenario of the fitness that can be obtained through random guessing. If the distribution of a resampling test deviates from this reference distribution, it means that some overfitting occurs, since the model should not perform better than random guessing. We can then compare the fitness distribution from the models using resampled target variables (**H0_modeled_**) not only with the fitness calculated from the model using the actual target variable (**fitness_actual_**) to assess the prediction performance, but also with the reference distribution (**H0_reference_**) to examine systematic deviations in fitness under any type of modeling conditions (Fig. 3 and Supplementary Fig. S3). A more detailed description of **H0_reference_** is available in the tutorial.

We examined fitness across four different CV strategies: (i) *fit-predict*, where the entire data are used both for training and testing, but hyperparameters (e.g. number of PLS components) are selected from single CV, similar to procedures employed in conventional software (Sadeghi-Bazargani *et al.*, 2010); (ii) Single cross-validation (*1CV*), where data are divided into training and test sets in folds and hyperparameter optimization, predictions, and fitness estimations are based on hold-out predictions. Importantly, the *1CV* terminology is thus different from that employed in several conventional software, which performs what we refer to as *fit-predict* above. (Sadeghi-Bazargani *et al.*, 2010); (iii) Double cross-validation (*2CV*), where data are divided into training, validation, and test set. The training and validation sets optimize hyperparameters and are used to build prediction models. The test set is thus held out from all training and hyperparameter tuning and used to evaluate model fitness. The predictions thus have less bias compared to *1CV* and finally; (iv) *MUVR2*, where recursive variable elimination is added in the *2CV* as previously described. For EN modeling, only *fit-predict*, *1CV*, and *2CV* were tested, since MUVR2-EN does not perform recursive variable elimination (as described in section 2.3) and therefore is identical to *2CV*.

To account for stochastic effects in the sampling into CV segments and achieve more stable predictions, the **fitness_actua_**_l_ was averaged over 50 repetitions. **H0_modeled_** distributions were obtained from *n* = 400 resampled target variables per CV strategy, averaged over 10 repetitions instead of 50 to decrease computation time. **H0_reference_** distributions were obtained from 1000 resamplings.

We further obtained *P*-values for the **fitness_actual_** versus **H0_modeled_** distributions using three strategies: (i) Non-parametrically, using the rank order of **fitness_actual_** and **H0_modeled_** distribution (Szymańska *et al.*, 2012); (ii) By assuming that **H0_modeled_** distribution follows a t-distribution and calculating cumulative probability (Shi *et al.*, 2019) and; (iii) As cumulative probabilities from a smoothed empirical distribution of **H0_modeled_**.

### 2.6 Software and hardware

All calculations were performed in the R Statistical software (v 4.2.1). The MUVR2 algorithm is freely available in the R package *MUVR2* together with data, tutorial, and scripts at https://github.com/MetaboComp/MUVR2. Due to the sensitive nature of the BioDiva data, it does not appear in the *MUVR2* package and calculations using the data were performed using the SNIC-SENS resources provided by the Uppsala Multidisciplinary Centre for Advanced Computational Science (https://www.uppmax.uu.se/). All other calculations were performed on a laptop computer with an 11^th^ Gen Intel i7 processor with eight cores and 32 GB internal memory.

## 3 Results

In the present work, we developed the *MUVR2* package through the incorporation of EN, but not SVM and ANN due to computational restraints. The incorporation of EN also allowed for simultaneous adjustment for covariates, which was not obtained using PLS and RF. In addition, we have extended the use of permutation tests into resampling tests, constituting a general framework for assessing modeling performance and overfitting. MUVR2 showed optimal prediction performance with the added benefit of automatic selection of features of interest without introducing overfitting.

### 3.1 Expanding ML strategies

Incorporating EN into MUVR2 allowed modeling with similar fitness and computational efficiency as PLS and RF (Table 1), even with tuning of both the alpha and lambda hyperparameters (Friedman *et al.*, 2010) (Table 1). Within one MUVR2-EN model, the variable selection was also shown to be stable across *nRep* × *nOuter* calibration set models (Supplementary Fig. S4a), where different calibration set models selected similar sets of variables, albeit using different segments of data. Consequently, MUVR2-EN was shown to produce stable variable importance ranks and selections also across re-analysis (Supplementary Fig. S4b).

**Table 1.** Computation time and prediction performance of machine learning methods in the MUVR2 framework, with each method’s default hyperparameters (*nRep = *5, *nOuter = *6, *varRatio = *0.75).

	Regression		Classification	
	Time	*Q* ^2^	Time	BER
PLS	0.31 min	0.48	0.54 min	0.21
RF	1.11 min	0.41	0.88 min	0.26
EN	0.36 min	0.39	1.64 min	0.22
SVM	125.49 min	0.12	49.47 min	0.67
ANN	>24 h	N/A	>24 h	N/A

Regression was tested using the Freelive2 data and using *Q*^2^ to assess fitness. The classification used the Mosquito data and BER for fitness. Results are reported as averages from triplicate analyses.

PLS, partial least squares; RF, random forest; EN, elastic net; SVM, support vector machine; ANN, artificial neural network; BER, balanced error rate; N/A, not available—calculation was aborted after >24-h computational time.

An important feature of the MUVR2 standard variable selection procedure is that modeling fitness is estimated with the same density over the range of the number of selected variables (Fig. 1a and b). However, MUVR2-EN does not perform recursive elimination at consistent intervals, and modeling fitness does not have such equal density. We therefore offer two alternatives: The preferred option, conceptually similar to the standard approach, estimates prediction performance as a function of the number of variables using locally weighted least squares regression (Gijbels and Prosdocimi, 2010) (Fig. 1c and d). However, the resulting curve may cover certain variable selection regions poorly and end up in irregular shapes, for example lacking a clearly defined global minimum, making final variable selection obscure. In this case, increasing the number of the calibration set models (*nRep*×*nOuter*) may help produce a more well-defined curve. The removal of outliers (Fig. 1d) can further improve fitness estimation in low-density areas. However, if visual inspection of the fitness curve implies poor fit, we offer the option of performing variable selection directly from the distribution of the number of non-zero beta coefficients from the calibration set models (Fig. 1e and f), which leads to faster-converging “min,” “mid,” and “max” variable selections, but disregards fitness at the various selections. A more detailed description of variable selection in MUVR2-EN is available in the tutorial.

Performing SVM within the MUVR2 framework was too computationally expensive for practical use (Table 1), likely resulting from the high number of variables, which makes the number of possible solutions increase exponentially (Noble, 2006). Also, prediction performance was low compared to PLS, RF, and EN (Table 1), likely due to the performance depending on kernel options and penalty parameters (Tharwat, 2019), which were not optimized due to the high computational time. In MUVR2-ANN, none of the examined procedures yielded stable variable importance ranks, likely related to the random initial node weights (Olden and Jackson, 2002, Olden *et al.*, 2004, Venkateswaran and Ciaburro, 2017). Although Olden’s algorithm has been accurate in quantifying variable importance in ANN using low-dimensional simulated data (Olden *et al.*, 2004) this may not hold true for large, real-life datasets. Additionally, relevant hyperparameters, such as the number of nodes in the hidden layer, learning rate, and the selection of activation function, need to be tuned in ANN to optimize the prediction fitness (Olden *et al.*, 2004). However, even without such hyperparameter tuning, the computational time required for MUVR2-ANN far exceeded PLS, RF, and even SVM, effectively prohibiting its use in MUVR2. We therefore excluded SVM and ANN from the MUVR2 framework (Table 1).

We further compared the minimal-optimal variable selections from MUVR2-PLS, MUVR2-RF, and MUVR2-EN in regression (Supplementary Fig. S5) and classification (Supplementary Fig. S6). Being a component-based method, PLS selects variables sharing similar variance patterns. RF instead upweighs complementarity between variables and thus achieves more parsimonious variable selections (Biau and Scornet, 2016). EN, on the other hand, being a linear variance-based method, tends to select the individually strongest predictors. In brief, all methods were able to identify a core set of relevant predictors, while different methods also produced unique variable selections likely reflecting their different operating principles. A detailed comparison is provided in Supplementary Text S1.

Additionally, we compared variable importance ranks from MUVR2-RF with averaged SHAP values obtained from 100 random resamples of observations and using *fit-predict* random forest modeling (Supplementary Fig. S7). Top-ranking variables in MUVR2 also had the highest SHAP values, thus strengthening the validity of the variable selection in MUVR2.

In addition, MUVR2 was also extended to support automatic one-hot encoding of categorical predictor variables. While support for categorical variables is native to RF and EN, this simplifies the use of nominal variables in PLS analysis (Hogan *et al.*, 2021). Moreover, users can customize if they would like to use one-hot-encoding or native support for categorical variables in RF and EN.

### 3.2 Covariate adjustment

According to the causal structure in the synthetic data (Fig. 2), the expected variable selection when not including any covariates among the predictors should prioritize all X_1–4_, but not X_5–100_. Upon adding and *keep*ing C_1–4_, we further expected that the importance of X_1_, X_3_, and X_4_ should decrease, and X_2_ remain unchanged. However, in MUVR2-PLS and MUVR2-RF, the results did not conform to these expectations for X_1_, X_3_, and X_4_ (Table 2), highlighting an important conclusion: Forcing covariates to be excluded from recursive elimination does not correspond to covariate adjustment for these methods. This likely reflects that covariates may not be fully used in the models: For PLS, latent variables calculated may not contain the full information of covariates. For RF, even if each model has access to the covariates, each node or even tree will not necessarily have such access.

**Table 2.** Variable importance ranks of predictors and covariates (lower is better), number of selected variables, and prediction performance (*Q*^2^) (median from 100 simulations in a regression using synthetic data generated according to the causal structure described in Supplementary Fig. S2).

	MUVR2-PLS			MUVR2-RF			MUVR2-EN		
	C_none_	C_add_	C_keep_	C_none_	C_add_	C_keep_	C_none_	C_add_	C_keep_
X_1_	1.0	2.0	1.0	1.0	2	1	1.0	3.0	73.5
X_2_	6.5	8.0	5.0	8.5	11.5	8.5	4.0	4.0	1.0
X_3_	5.5	8.0	5.0	6.0	9.0	8.0	3.0	6.0	54.5
X_4_	9.0	13.5	10.0	13.5	16.0	14.5	6.5	16.0	92.0
C_1_	N/A	2.0	0^a^	N/A	2.0	0^a^	N/A	4.0	0^a^
C_2_	N/A	10.0	0^a^	N/A	14.0	0^a^	N/A	4.0	0^a^
C_3_	N/A	12.0	0^a^	N/A	12.5	0^a^	N/A	21.0	0^a^
C_4_	N/A	8.5	0^a^	N/A	12.0	0^a^	N/A	8.0	0^a^
nVar	29	33	32	8	9	10	30	43	50
*Q* ^2^	0.18	0.34	0.38	0.17	0.27	0.31	0.26	0.36	0.51

Three modeling approaches, C_none_, C_add_, C_keep_ were tested with MUVR2-PLS, RF and EN. Only MUVR2-EN conformed to the expectation of decreased ranks of X_1_, X_3_, and X_4_ upon *keep*ing C (i.e. C_1_, C_2_, C_3_, C_4_).

C_none_, model using only X (i.e. X_1_, X_2_ ….…X_100_) as predictors; C_add_, model using X and C as predictors, but not *keep*ing C; C_keep_, as C_add_, but *keep*ing C; nVar, number of selected variables by the “max” model; N/A, not available since the covariate was not included in modeling.

a Represents that a model *keep*s the variable and the variable was therefore excluded from ranking.

Using MUVR2-EN, however, the variable importance for X_1_, X_3_, and X_4_ decreased as expected (Table 2). This should come as no surprise since EN builds regularized linear models, where the full information of the covariates is always included in the model (Zou and Hastie, 2005, De Mol *et al.*, 2009). A more detailed description is available in Supplementary Text S2. Similar results were obtained when testing the four causal structures in Fig. 2 separately (data not shown).

We further investigated adjusting for sex as a potential confounder in MUVR2-EN, when associating metabolite features (predictors) to T2D status (target variable) in the real-world BioDiva data. We assumed that predictors affected by confounding would be selected less often among the calibration set models in MUVR2-EN when *keep*ing sex, compared with not *keep*ing them, which was confirmed by observation (Supplementary Figs S8 and S9). A more detailed description is available in Supplementary Text S3.

Thus, suppressing EN regularization from covariates influences how predictors are selected using MUVR2-EN, corresponding to covariate adjustment. In fact, EN has previously been reported for its effective covariate adjustment in high-dimensional problems (Yue *et al.*, 2019). However, it is nonetheless reassuring that both synthetic and real-world data support this notion also in MUVR2-EN.

### 3.3 Assessing modeling fitness and overfitting using resampling tests

We introduced a new strategy for resampling tests, where target variables under the null-hypothesis are simulated from an empirical distribution rather than fixed probabilities. This increased variability should reflect in more realistic (higher) *P*-values compared to permutation tests. In addition, we must acknowledge that not only the actual fitness (**fitness_actual_**), but also the permutation (or resampling) distribution (**H0_modeled_**) could suffer from overfitting, hence impeding inference. To evaluate such systematic overfitting, we introduced a reference state (**H0_reference_**), which could help assess whether there is a suitable match between the data and the model (including the choice of ML method and CV strategy): Differences between the **H0_modeled_** and **H0_reference_** distributions could imply that the modeling strategy may not be adequately suited for the data, possibly from general overfitting.

To assess the *P*-value of **fitness_actual_** versus **H0_modeled_**, previous approaches have used either the rank order of **fitness_actual_** in the **H0_modeled_** distribution (Szymańska *et al.*, 2012) or calculated the *P*-value from the cumulative probability under the assumption that H0 is t-distributed (Shi *et al.*, 2018). Both these approaches are problematic: The former cannot quantitate *P*-values below 1/nPerm (Szymańska *et al.*, 2012) and we have frequently observed that **H0_modeled_** is not well-represented by a t-distribution (Supplementary Fig. S10). We instead opt to calculate *P*-value as the cumulative probability in the empirical **H0_modeled_** distribution, represented by a smoothed curve. This is conceptually similar to calculating *P*-values from *t*-values in a Student’s *t*-distribution or *z*-values in a normal distribution. Simulations showed that this approach generated *P*-values similar to those from the t-distribution approach when H0 was Gaussian (data not shown) and was also able to generate *P*-value estimates from non-Gaussian distributions that better corresponded to intuitive assessment (Supplementary Fig. S10).

In the regression example, we observed higher *Q*^2^ for **H0_modeled_** compared to **H0_reference_** for all ML methods and CV strategies (Fig. 3). This implies structural overfitting, effectively limiting the certainty by which we can draw inference from the models. We further compared resampling test to permutation test, which showed similar discrepancies (data not shown). We also compared to an alternative permutation approach based on resampling both predictor and target variables, again showing a similar discrepancy between **H0_modeled_** and **H0_reference_** (data not shown). Additionally, PLS modeling generated **H0_modeled_** distributions more similar to **H0_reference_** compared to RF and EN. We speculate that different ML methods may be differentially sensitive to the change from the actual target variable to resampled target variables, using this specific data.

In general, *fit-predict* models showed high *Q*^2^ for the actual modeling in PLS, RF, and EN and a large difference between **H0_modeled_** and **H0_reference_**, suggesting that performance was indeed driven by overfitting. Thus, *P*-values cannot be trusted to accurately represent the underlying difference between **fitness_actual_** and **H0_modeled_** and the actual model cannot be trusted for inference. With increasing complexity in CV, we observe that the **H0_modeled_** distribution becomes more similar to the reference distribution. However, it should be noted that the largest leap in reducing modeling overfitting compared to *fit-predict* comes from incorporating holdout predictions—regardless of the CV complexity. More complex CV procedures indeed seem to reduce general modeling overfitting additionally, albeit not to the same extent. This also conforms to Westerhuis’s permutation tests comparing *fit-predict*, *1CV*, and *2CV* (Westerhuis *et al.*, 2008).

**Fitness_actual_** was also affected by the CV procedure. As expected, going from *fit-predict* to *1CV*, the **fitness_actual_** decreased due to lower degree of overfitting. Going from *1CV* to *2CV*, the **fitness_actual_** increased, which we interpret as improved generalizability from the nested CV procedure, effectively leveraging on the variance-bias tradeoff (Belkin *et al.*, 2019). Importantly, the added element of variable selection in *MUVR2* compared to *2CV* did not strongly affect **fitness_actual_** or **H0_modeled_**. This observation strengthens the notion that the MUVR2 procedure has considerable informatics benefits from achieving an automated selection of variables of interest largely without affecting prediction performance or imposing bias. These trends were confirmed also in the classification example (Supplementary Fig. S3), that is we similarly observed that the largest reduction in general modeling overfitting comes from employing CV for holdout predictions, that the nested CV procedures seem to boost prediction generalizability and that the MUVR2 variable selection does not impose overfitting.

In summary, the comparison between **H0_modeled_** and **H0_reference_** provides useful information about the suitability between model and data. We thus propose that resampling tests and reference distributions can be used as a general framework to assess prediction performance and overfitting in ML modeling, as well as a tool for providing data-driven choices of ML modeling strategies. Nevertheless, it remains apparent that the area of model evaluation merits further investigation.

### 3.4 Strengths and limitations

A major strength of the MUVR2 algorithm is the state-of-the-art nested CV to ensure minimal overfitting (Westerhuis *et al.*, 2008, Filzmoser *et al.*, 2009, Shi *et al.*, 2019). Another strength is that MUVR2 performs variable selection within the nested CV, which we have shown does not impose overfitting. However, computations are also time-demanding compared to simpler CV frameworks. Future versions should consider porting implementation to faster languages (Eddelbuettel, 2013, Krasnovidov and Khomonenko, 2021). We also showed that the EN method in MUVR2 effectively adjusts for covariates, which provides opportunities for ML in epidemiological studies. We have further expanded from permutation tests into resampling tests, including a comparison to a reference state, which provides a framework to assess prediction performance and overfitting. However, investigations should be extended to additional datasets, for example proteomics data, and other metabolomics data. In addition, more research is required in the area of modeling fitness evaluation.

## 4 Concluding remarks

In addition to partial least squares and random forest, the *MUVR2* package was extended from the original MUVR framework to include EN for modeling and variable selection within repeated *2CV*. Using simulated and real-world data, we showed that this addition provided possibilities for covariate adjustment directly during ML analysis while maintaining the highest levels of safeguards against overfitting. Moreover, we introduced a framework for the systematic assessment of modeling fitness and overfitting based on resampling tests and a reference distribution for fitness under null-hypothesis conditions. Comparing MUVR2 to other CV strategies, we showed that it performs prediction at a state-of-the-art level and also performs variable selection without imposing additional overfitting compared to nested CV, which has significant informatics benefits. While applications reported herein have focused on the analysis of metabolomics data, MUVR2 can also be applied to other types of high-dimensional data where variable selection is of interest.

## Supplementary Material

vbae051_Supplementary_Data
