# Supplementary material for: Adjusting for covariates and assessing modeling fitness in machine learning using MUVR2
Source: Bioinform Adv. 2024 Apr 4;4(1):vbae051. doi: 10.1093/bioadv/vbae051 (PMC11031361; doi:10.1093/bioadv/vbae051)
Supplement: vbae051_Supplementary_Data [file vbae051_supplementary_data.zip › Revised_Supplemental_material_cleaned.docx]

# **Supplemental material belonging to the manuscript:**

**Adjusting for covariates and assessing modeling fitness in machine learning using MUVR2**

Yingxiao Yan^1^, Tessa Schillemans^2^, Viktor Skantze^3^, Carl Brunius^1,4^

###

*^1^ Department of Life Science, Chalmers University of Technology, SE-41296 Gothenburg, Sweden.*

*^2^Cardiovascular and Nutritional Epidemiology, Institute of Environmental Medicine, Karolinska Institutet, SE-171 77 Stockholm, Sweden.*

*^3^ Fraunhofer-Chalmers Research Centre for Industrial Mathematics, Gothenburg, Sweden*

*^4^ Chalmers Mass Spectrometry Infrastructure, Chalmers University of Technology, SE-41296 Gothenburg, Sweden.*

**Supplemental Material**

**1. Supplemental texts**

Supplemental text 1: Comparing variable selection in MUVR2-PLS, MUVR2-RF and MUVR2-EN to examine similarities and differences.

Supplemental text 2: Testing covariate adjustment in MUVR2 using simulated data

Supplemental text 3: Testing covariate adjustment in MUVR2 using real-world data

**2. Supplemental Figures**

Figure S1-S10

**3. Supplemental Table**

Table S1

***Supplemental text 1***

**Comparing variable selection in MUVR2-PLS, MUVR2-RF and MUVR2-EN to examine similarities and differences.**

In the regression example (**Figure S5**), besides the small set of core variables selected by all three methods (marked in red), a group of variables that were selected by MUVR2-PLS and MUVR2-EN showed strong inter-correlations (marked in blue), implying that these variables capture common information that associates with the target variable linearly, given that both PLS and EN are linear methods. The fact that they were selected using PLS highlights the shared variance pattern of these variables, which contributes to an increased signal-to-noise ratio in the underlying PLS components, whereas the selection using EN implies that these variables are the strongest individual predictors (i.e., sharing the strongest covariance with the target variable). These variables were not selected using RF, which further suggests that they may constitute redundant information that does not contribute to an exclusive predictive capacity beyond the shared variance pattern.

Among the variables selected using EN, there is also a group of inter-correlating variables (marked in green) that do not correlate to the blue pattern, potentially suggesting biologically independent mechanisms. Interestingly, it appears that both the blue and the green variance patterns are represented in the small core set of predictors (in red), indicating that these core predictors may capture the latent information in both patterns. Moreover, EN picks up on an additional group of variables (marked in black), not represented by the two patterns discussed directly, but rather anticorrelated, suggesting involvement in both mechanisms.

In the classification example (**Figure S6**), the core set of variables selected by all methods and additional variables selected by both MUVR2-PLS and RF were strongly correlated (marked in red), representing one distinct microbiota pattern. In addition, there was another pattern (marked in green), which associated inversely with the red pattern. However, the variables in the green pattern did not overlap between the three methods and also showed a larger variability in their inter-correlations. This discrepancy may reflect a potential downside in using ML for variable selection, i.e. that different inference and biological interpretation can be drawn when variable selections are different between methods. In such a scenario, EN may have an advantage due to its fundamental linear modeling strategy, which is more directly interpretable than other ML strategies.

***Supplemental text 2***

**Testing covariate adjustment in MUVR2 using simulated data**

Based on the causal structures and parameters described in **Figure S2**, we simulated data to obtain 100 predictors (i.e. X_1_ … X_100_), 4 covariates (i.e. C_1_, C_2_, C_3_ and C_4_) and a target variable Y for 50 observations. We then performed MUVR2-PLS, MUVR2-RF and MUVR2-EN and constructed 3 models for each ML method: C_none_ models the target variable using 100 X predictors, while C_add_ also included covariates along with the predictors but without *keep*ing them. C_keep_ additionally *keep*s all covariates, i.e. omitting the covariates from elimination in MUVR2-PLS and RF and suppressing regularization for these covariates in MUVR2-EN. Variable importance ranks for predictors of interest (X_1-4_) and covariates were extracted from the maximal variable selection in MUVR2, along with the number of selected variables and prediction performance (Q^2^) (**Table 2**). To reduce the impact of stochastic effects, the procedure was repeated 100 times, keeping only models with Q^2^ >= 0.1 in order not to draw inference from uninformative models. We then reported and compared median variable importance ranks, the average number of selected variables and the average Q^2^.

Difference in predictors’ variable importance ranks between C_none_ and C_add_ captures the effect of including covariates in the model while the rank difference between C_add_ and C_keep_ informs whether predictors are influenced by *keep*ing covariates. To effectively filter out noise, we only compare the median ranks of a predictor of interest when it is at least ranked within the first 20% of the features in C_none_ and C_add_ (i.e. rank within 1-20).

Our expectations were that: First, C_none_ and C_add_ will give similar variable importance ranks for X_1-4_ in all MUVR2 ML methods since merely including a covariate in the machine learning model as an ordinary predictor may not achieve the purpose of covariate adjustment. Second, when *keep*ing covariates in MUVR2 in C_keep_, the variable importance ranks of X_1_ should decrease because C_1_ serves as a confounder for the association between X_1_ and Y; The variable importance rank of X_2_ should remain unchanged. Since the corresponding covariate (i.e. C_2_) of X_2_ is not associated with Y through X_2_, the addition of C_2_ may contribute to the prediction of Y but may not influence X_2_’s contribution to the prediction; The ranks of X_3_ and X_4_ are expected to decrease after *keep*ing their covariates (i.e. C_3_ and C_4_) in MUVR2 since X_3_ is a mediator for the pathway from C_3_ to Y and C_4_ is a mediator for the pathway from X_4_ to Y.

While our first expectation was fulfilled and the expectation of similar ranks for X_2_ between model C_add_ and C_keep_ was confirmed using all modeling strategies (PLS, RF and EN), the assumption of decreased ranks for X_1_, X_3_ and X_4_ was not fulfilled in MUVR2-PLS and MUVR2-RF (**Table 2**). This may be explained by the fact that PLS and RF in MUVR2, as machine learning methods, do not perform variable selection directly on the original predictors. PLS projects data on the lower dimension space and RF’s bootstrapping resamples the predictors with replacement. In this process, the latent variables in PLS may not capture the full information in covariates and the covariates are not necessarily included in each RF tree. The information for the associations between the specific predictors and covariates may thus drown in the information of all the other variables in the transformation. Therefore, when a covariate that is associated with a predictor is reserved in MUVR2’s nested loops, the variable importance ranks of the predictor may not necessarily be affected.

We have further investigated MUVR2-RF implementation of forced inclusion of covariates in the variables tried for splitting, using the *ranger* package. However, the forced inclusion of covariates in the tried variables for each node also did not correspond to the expected behavior. Other approaches to adjust for covariates in RF modeling are thus required.

On the contrary, ranks for X_1_, X_3_ and X_4_ indeed decreased in MUVR2-EN. Elastic net, as the combination of 2 regularization methods on a univariate linear regression, performs variable selection on the original predictors more intuitively by focusing on variables that have non-zero beta coefficients. In MUVR2-EN, *keep*ing a covariate means forcing a covariate to have a non-zero beta coefficient in each model across the repetition and outer CV loop. This has effectively prevented the information loss that exists in MUVR2-PLS and MUVR2-RF. Therefore, the variance of a model can more likely be explained by a covariate and the correlated predictor variables may more frequently exhibit a zero-beta coefficient, representing lower variable importance.

We also performed the analyses using variable importance ranks from the ‘min’ and ‘mid’ variable selections and obtained similar results (data not shown).

***Supplemental text 3***

**Testing covariate adjustment in MUVR2 using real-world data**

Using the BioDiva data (842 observations, 24758 features), which describes the metabolic profiles from a nested case-control study on 421 individuals who later developed type-2 diabetes and their individually matched controls, we similarly performed 3 models in MUVR2-EN classification (nRep = 30, nOuter = 6), each using future diabetes status as the target variable. C_none_ included only metabolic features among the predictors. C_add_ additionally included sex as a predictor without *keep*ing it and C_keep_ further *kept* sex. All models resulted in BER<0.25, implying informative modeling scenarios, thus suitable for variable selection.

Similar variable importance ranks were observed in C_none_ and C_add_ (data not shown), implying that merely including sex in modeling does not influence variable selection. Out of the 2057 metabolite features selected in C_add_ using minimal variable selection by quantiles (see manuscript and tutorial), 286 features associated with sex at Bonferroni-adjusted p-value < 0.05.

We then compared the selection ratio of how many times the sex-correlated features were selected (i.e. having non-zero beta coefficient) over the 30 x 6 = 180 calibration set models, with (C_keep_) and without (C_add_) *keep*ing sex. If *keep*ing a variable does not influence variable selection in MUVR2-EN, the distribution of change in the selection ratio should be normally distributed around zero. We thus expected that the distribution should be left-skewed, given that *keep*ing covariates may diminish the contribution of covariate-related features to prediction, which may effectively decrease their selection ratios in C_keep_.

The distribution for sex-correlated features was indeed shown to be left-skewed (**Figure S8**) and we therefore further investigated the strength of these features’ correlations with sex. The change in selection ratio among features with weak correlations with sex were, as expected, randomly distributed around zero, confirming that *keep*ing the covariate did not exert a major influence on the variable selection for these features. In addition, we also observed that features correlating more strongly with sex were less likely to be selected upon *keep*ing sex (**Figure S9**), thus confirming that *keep*ing covariates affects the selection ratio of covariate-related predictors.

With the same settings, we also performed similar models using age as the covariate instead of sex. Among the 684 features selected in the C_add_ model, 108 features were associated with age at Bonferroni-adjusted p-value < 0.05. However, changes in the selection ratio were only minor, which was also reflected by the features in general having weak correlations with age (data not shown).

These results confirm that *keep*ing a covariate does not affect the selection ratio when the covariate is not meaningfully affecting the feature-target variable association.

**Supplemental Figures**


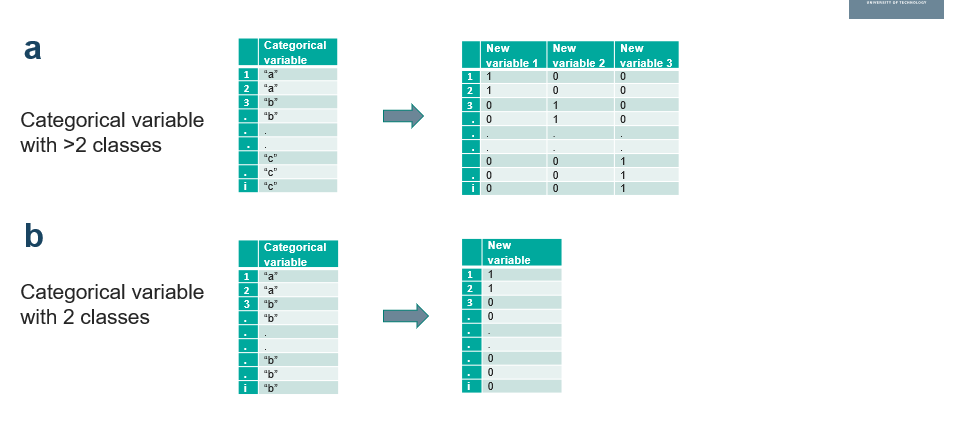


**Figure S1**. a. Illustration of one-hot-encoding for categorical variables a. with more than 2 classes. b. with 2 classes.


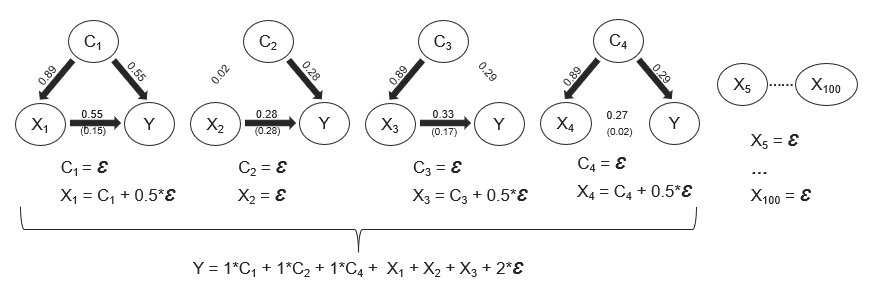


**Figure S2** Causal structures between simulated predictors (X, n = 100), covariates (C, n = 4) and the target variable Y. ***Ɛ*** represents random noise with a standard normal distribution. Covariates, predictors and the target variable were generated following the temporal order of the causal structure: First, covariates C_1_, C_2_, C_3_ and C_4_ were generated randomly from a standard normal distribution; Then X_1_, X_3_ and X_4_ were generated from their corresponding covariates, while X_2_, X_5_……X_100_ were generated randomly from a standard normal distribution. Finally, the target variable was generated as having a linear relationship with C_1_, C_2_, C_4_, X_1_, X_2_ and X_3_. The average coefficients of the Spearman correlation between predictors of interest, corresponding covariates and the target variable over 100 data simulations are reported in the figure, with average partial Spearman correlation coefficients (adjusting for the corresponding C) in parentheses.


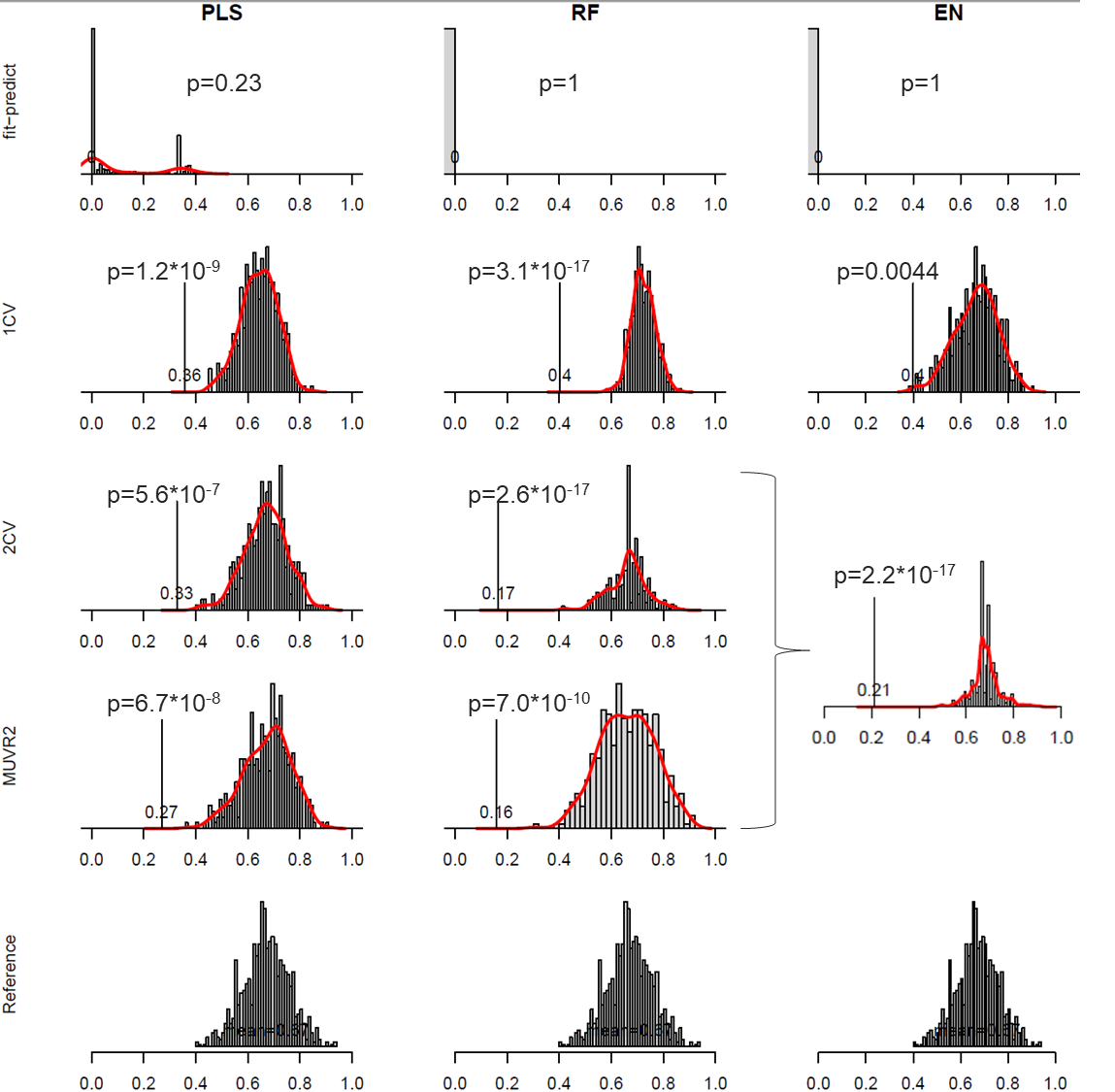


**Figure S3.** Predictive performance in classification (BER) for actual modeling (**fitness_actua_**_l_; vertical lines) and resampling tests (**H0_modelled_**; histograms and smoothed curves) and reference distribution from resampling the target variable without modeling **(H0_reference_**; histograms at bottom). MUVR2 modeling was performed using PLS (left), RF (middle) and EN (right) with different validation strategies, including *fit-predict*, *1CV*, *2CV* and *MUVR2* (except for EN, since *2CV* is identical to *MUVR2*) using the Mosquito data. p-values were generated from the smoothed curve of the **H0_modelled_** distribution. Partial least squares, PLS; Random forest, RF; Elastic net, EN; BER, balanced error rate.


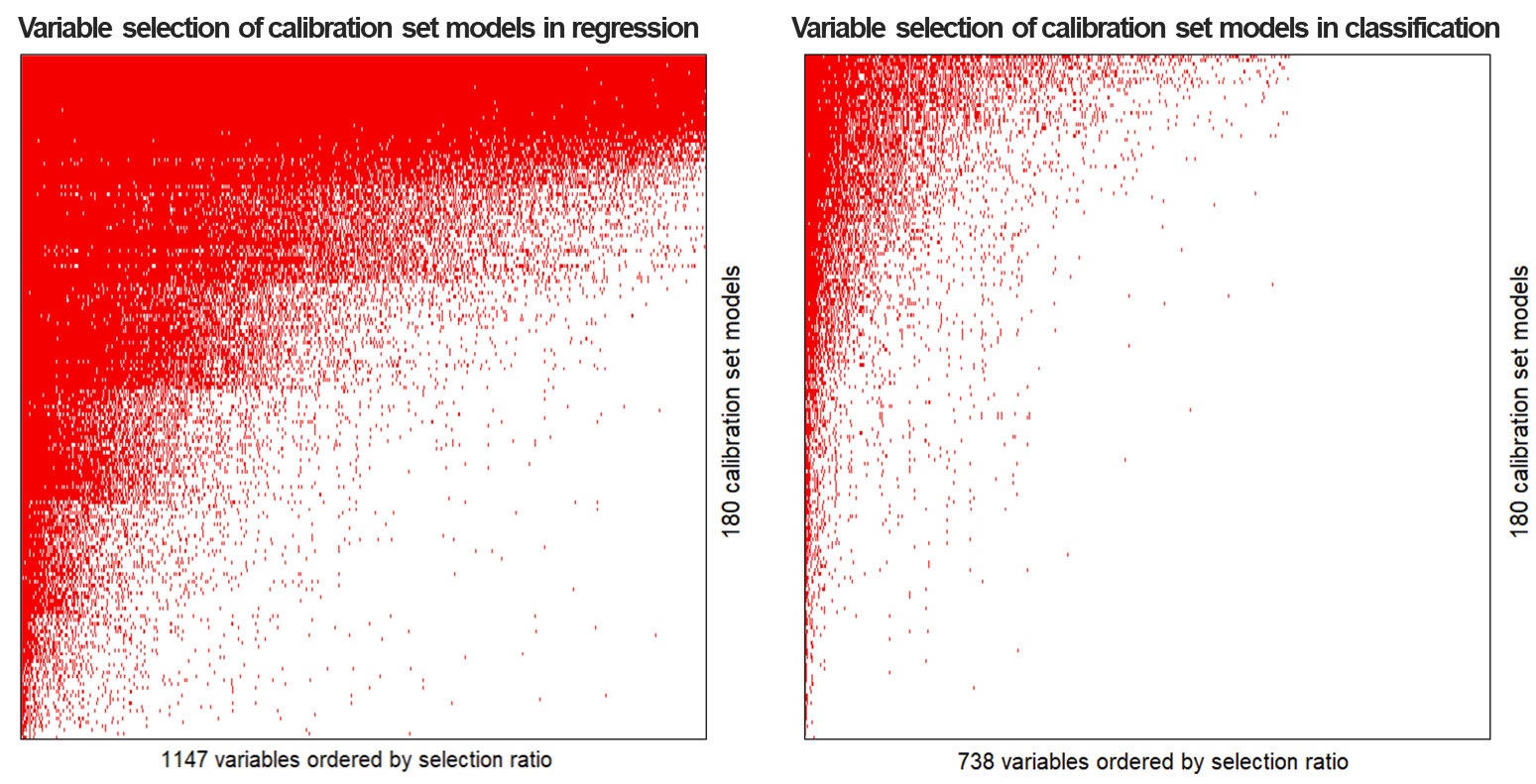


**a**


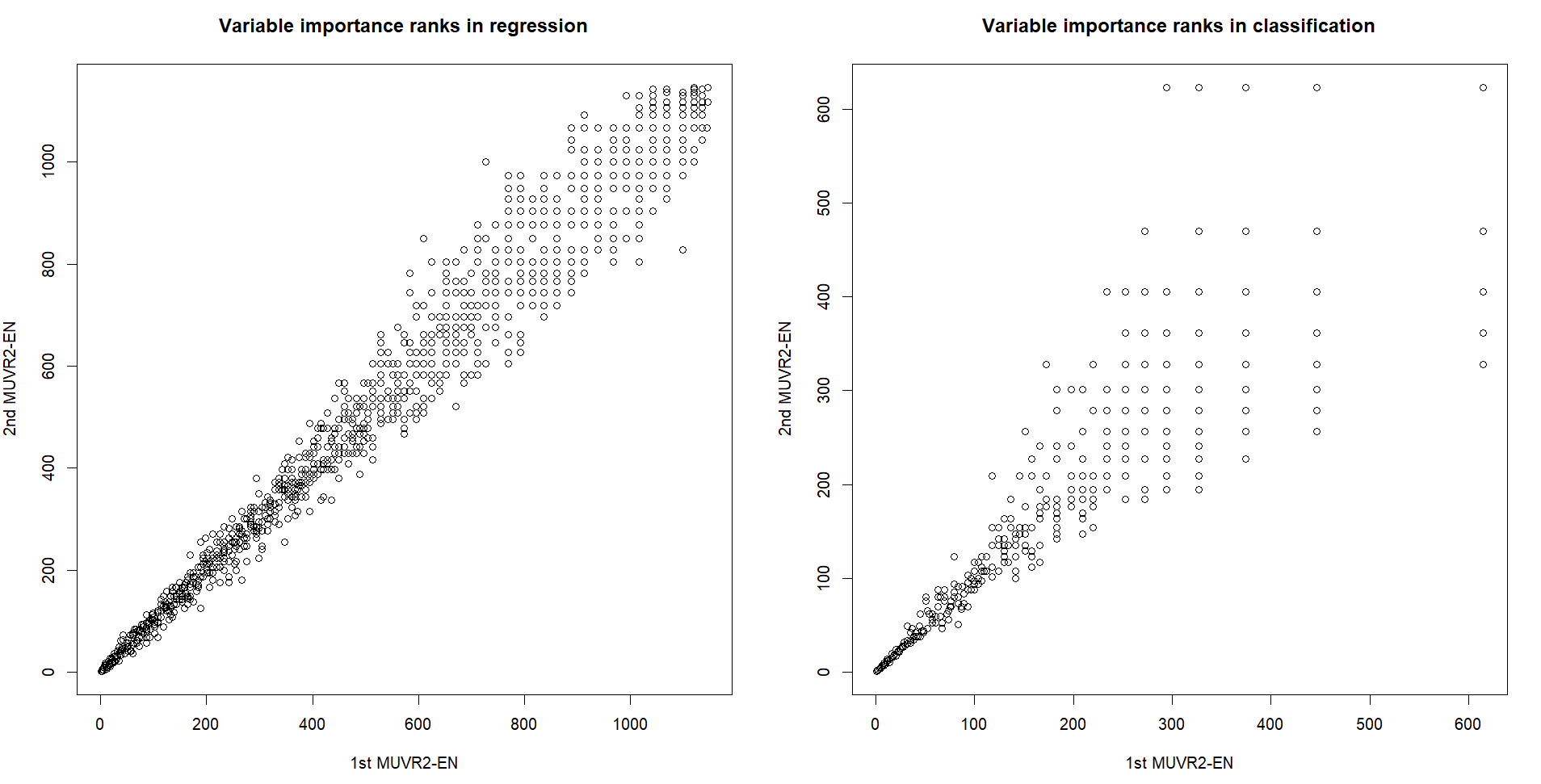


**bb**

**Figure S4**. Stability of variable selection and variable importance ranks in MUVR2-EN (nRep = 30, nOuter = 6) performed on the Freelive 2.0 data for regression (58 observations, 1147 variables) and the Mosquito data for classification (29 observations, 738 variables). **a)** Each row represents one calibration set model (*nRep* x *nOuter* models) and each column represents a variable. Red denotes that the variable had a non-zero beta coefficient in the corresponding calibration set model (i.e., being selected in the model). Calibration set models are ordered from selecting the most to the fewest variables and variables are sorted based on selection rates. Despite a wide variability in the number of selected variables, a core set of variables was systematically selected. **b)** MUVR2-EN was performed twice in both regression and classification (nRep = 30, nOuter = 6). Variable importance ranks (lower is better) show a high degree of stability. EN, elastic net.


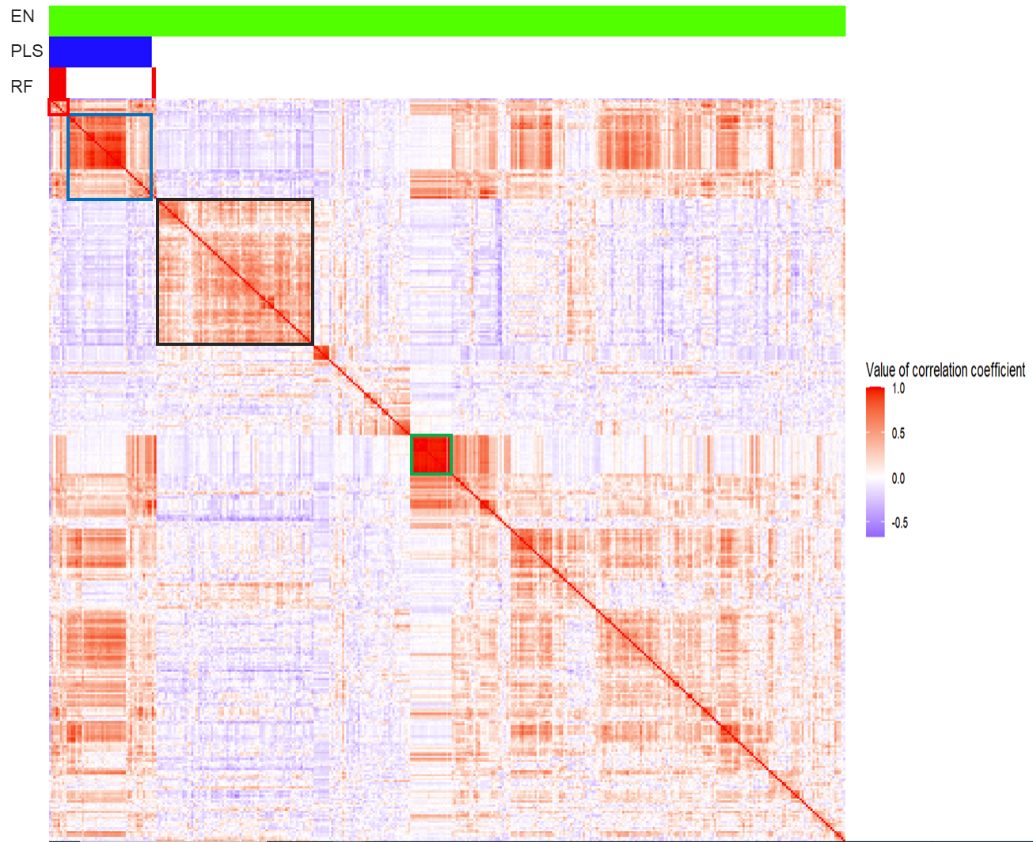


**Figure S5.** Pearson correlation coefficients between variables selected by the ‘min’ consensus models in MUVR2-PLS, MUVR2-RF and MUVR2-EN in regression (nRep = 30, nOuter = 6, varRatio = 0.75 (not applicable for EN)) using the Freelive 2.0 data (58 observations, 1147 variables). In total, 364 variables were selected by at least one method (47 by MUVR2-PLS, 10 by MUVR2-RF, and 364 by MUVR2-EN). Variables were grouped by what methods they were selected and further clustered based on the similarities (1- Pearson correlation coefficient) within each group. PLS, partial least squares; RF, random forest; EN, elastic net.


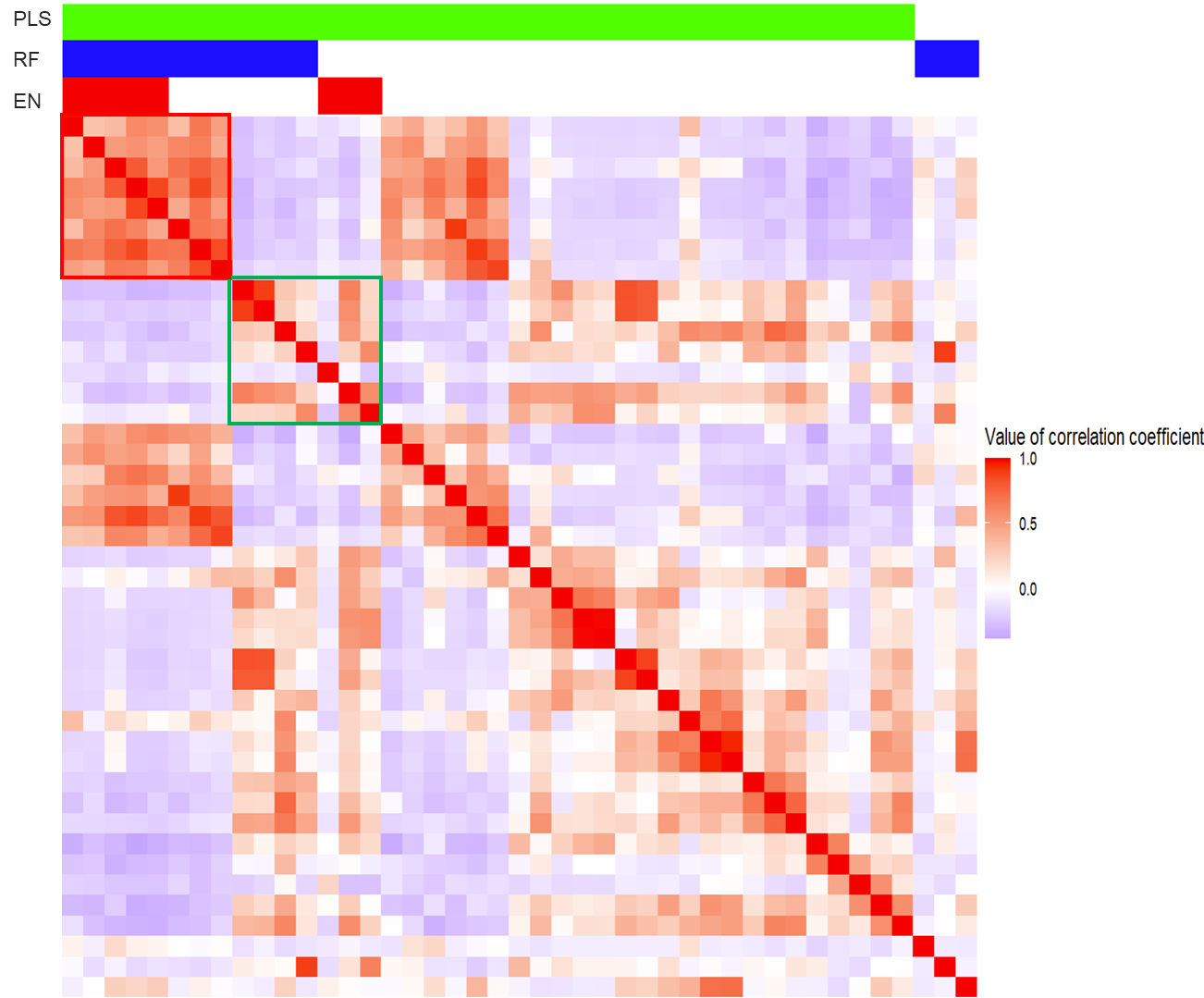


**Figure S6.** Pearson correlation coefficients between variables selected by the ‘min’ consensus models in MUVR2-PLS, MUVR2-RF and MUVR2-EN in classification (nRep = 30, nOuter = 6 , varRatio = 0.75 (not applicable for EN)) using the mosquito data (29 observations, 738 variables). In total, 43 variables were selected by at least one method (40 by MUVR2-PLS, 15 by MUVR2-RF, and 8 (**Figure 1f**) by MUVR2-EN). Variables were grouped by what method they were selected and further clustered based on the similarities (1- Pearson correlation coefficient) within each group. PLS, partial least squares; RF, random forest; EN, elastic net.

**
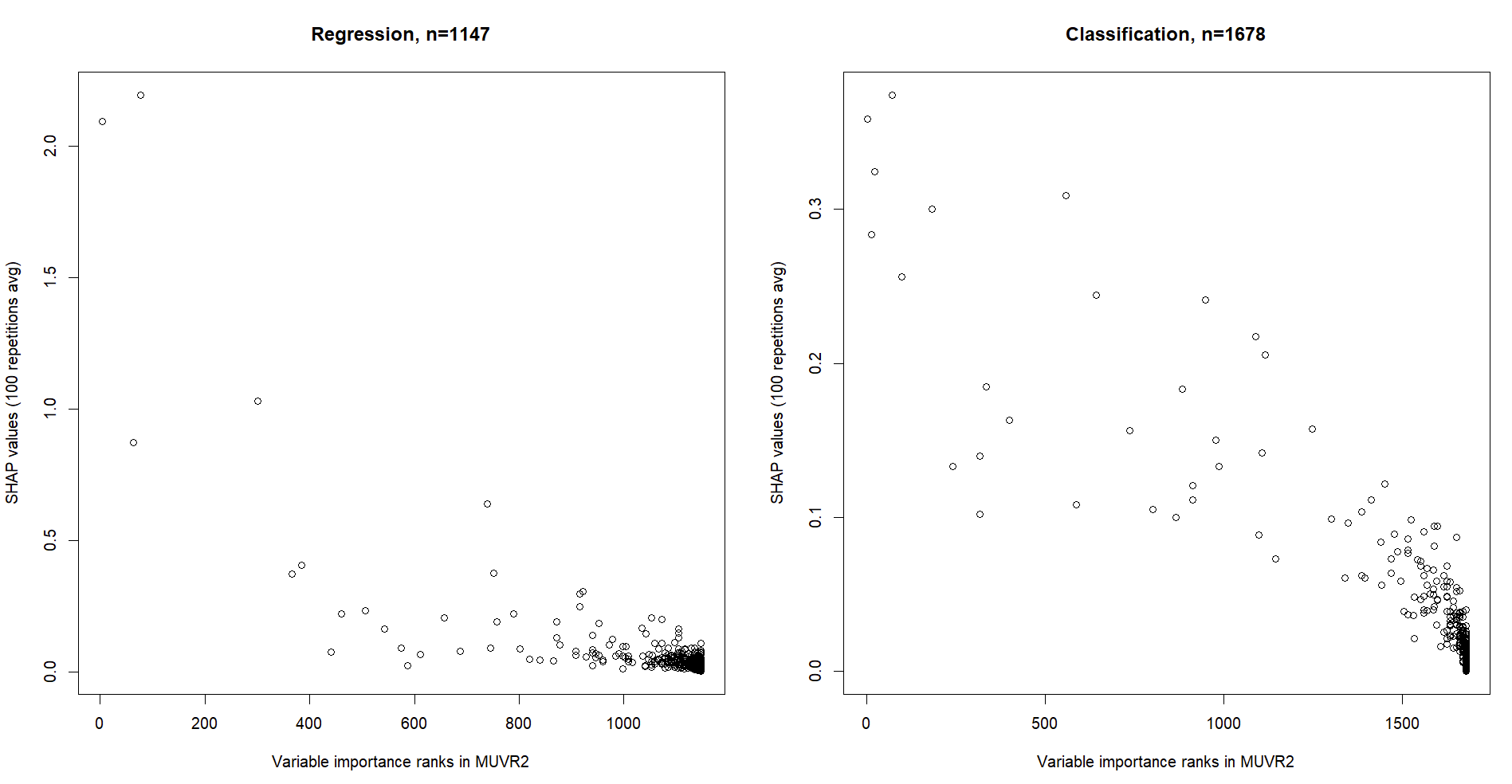
**

**Figure S7.** Comparison between variable importance ranks from MUVR2-RF and the SHAP values calculated from *fit-predict* random forest modeling. The SHAP value is averaged from SHAP values calculated from 100 resampling of observations. The figure on the left represents regression using the Freelive 2.0 data (58 observations, 1147 variables). The figure on the right represents classification using the Mosquito data (29 observations, 1678 variables). SHAP, Shapley additive explanations.


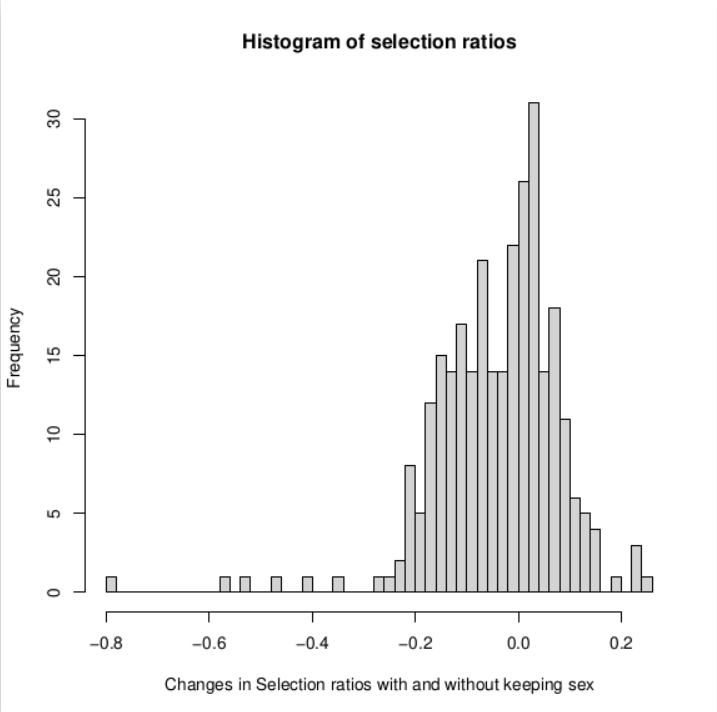


**Figure S8**. Change in selection ratios, i.e. difference in selection with (C_keep_) and without (C_add_) *keep*ing sex in MUVR2-EN’s 180 calibration set models (nRep = 30, nOuter = 6), shown for 286 metabolite features that were selected by the minimal variable selection of MUVR2-EN without *keep*ing sex and also correlated with sex at Bonferroni-adjusted p-value < 0.05.


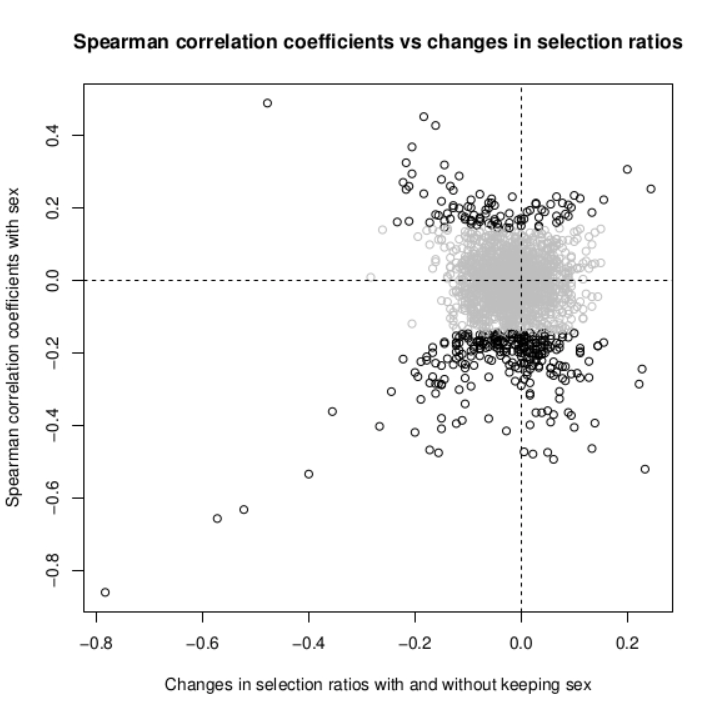


**Figure S9.** Association between sex-association (Spearman correlation between metabolite features and sex on the y-axis) and variable selection ratio (difference in selection with (C_keep_) and without (C_add_) *keep*ing sex in MUVR2-EN’s 180 calibration set models (nRep = 30, nOuter = 6) on the x-axis), shown for 2057 metabolite features that were selected by the minimal variable selection of MUVR2-EN without *keep*ing sex. Black denotes the 286 features that were also correlated with sex at Bonferroni-adjusted p-value < 0.05.


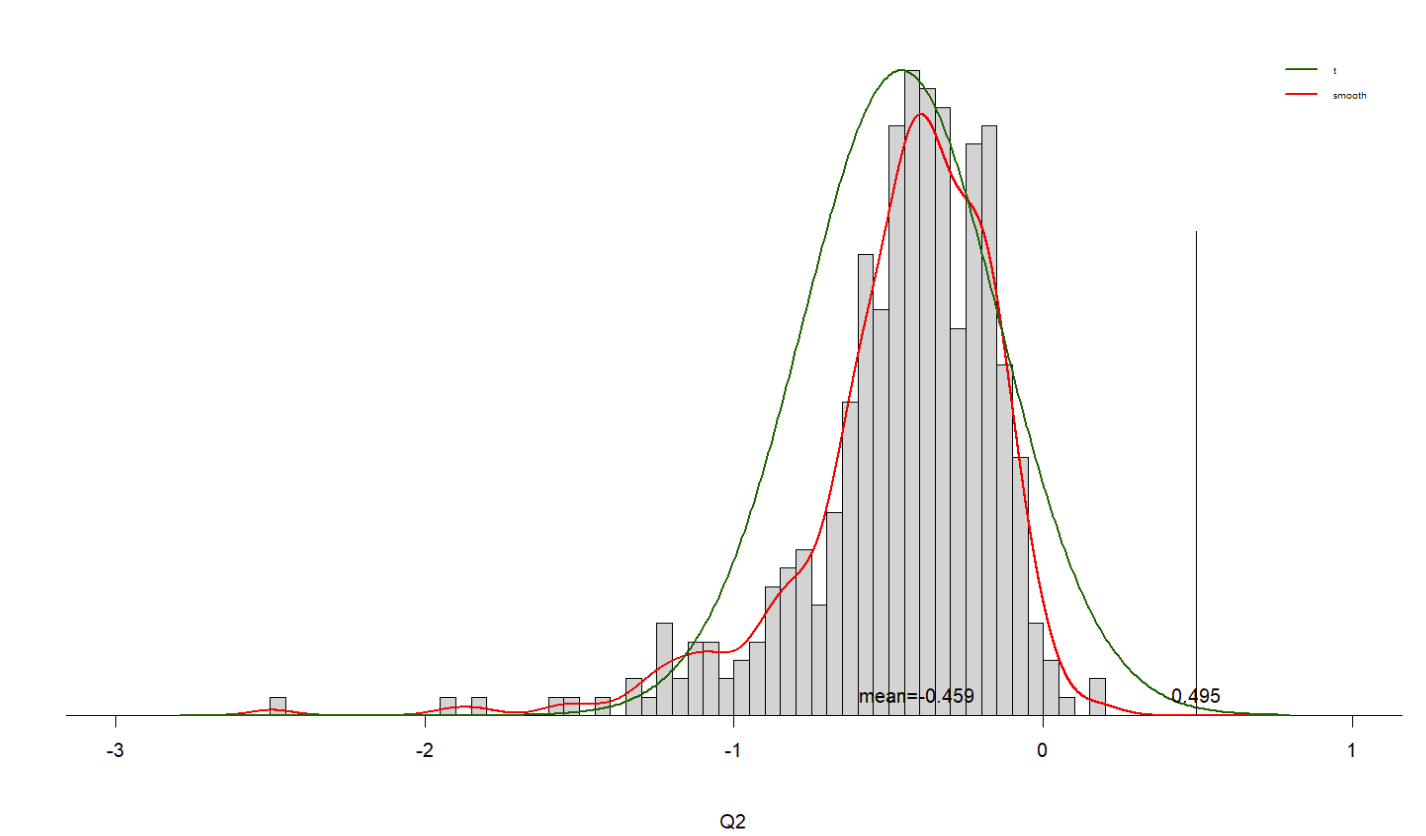


p_rank_ = 0.0025

p_t_ = 0.00153

p_smooth_ = 1.52*10^-8^

**Figure S10.** An example of comparing p-values by rank order (p_rank_), t-distribution (p_t_), and smoothed empirical distribution (p_smooth_) using predictive performance in regression (Q^2^) for actual modeling (**fitness_actual_**, vertical line) from resampling tests (**H0_modelled_** distribution of 400 values < **fitness_actual_**). The example uses MUVR2-PLS and the Freelive 2.0 data (58 observations, 1147 variables). A curve fitted for the t-distribution approximated from the values in the **H0_modelled_** distribution is shown in green, which reflects the **H0_modelled_** distribution poorly. A smoothed curve generated directly from the values in the **H0_modelled_** distribution is shown in red. PLS, Partial least squares.
