# Supplementary material for: Adjusting for covariates and assessing modeling fitness in machine learning using MUVR2
Source: Bioinform Adv. 2024 Apr 4;4(1):vbae051. doi: 10.1093/bioadv/vbae051 (PMC11031361; doi:10.1093/bioadv/vbae051)
Supplement: vbae051_Supplementary_Data [file vbae051_supplementary_data.zip › Revised_tutorial_cleaned.docx]

A Brief Tutorial on MUVR2:

Multivariate methods with Unbiased Variable selection in R

Yingxiao Yan and Carl Brunius, Gothenburg, 22 Jan 2024

#
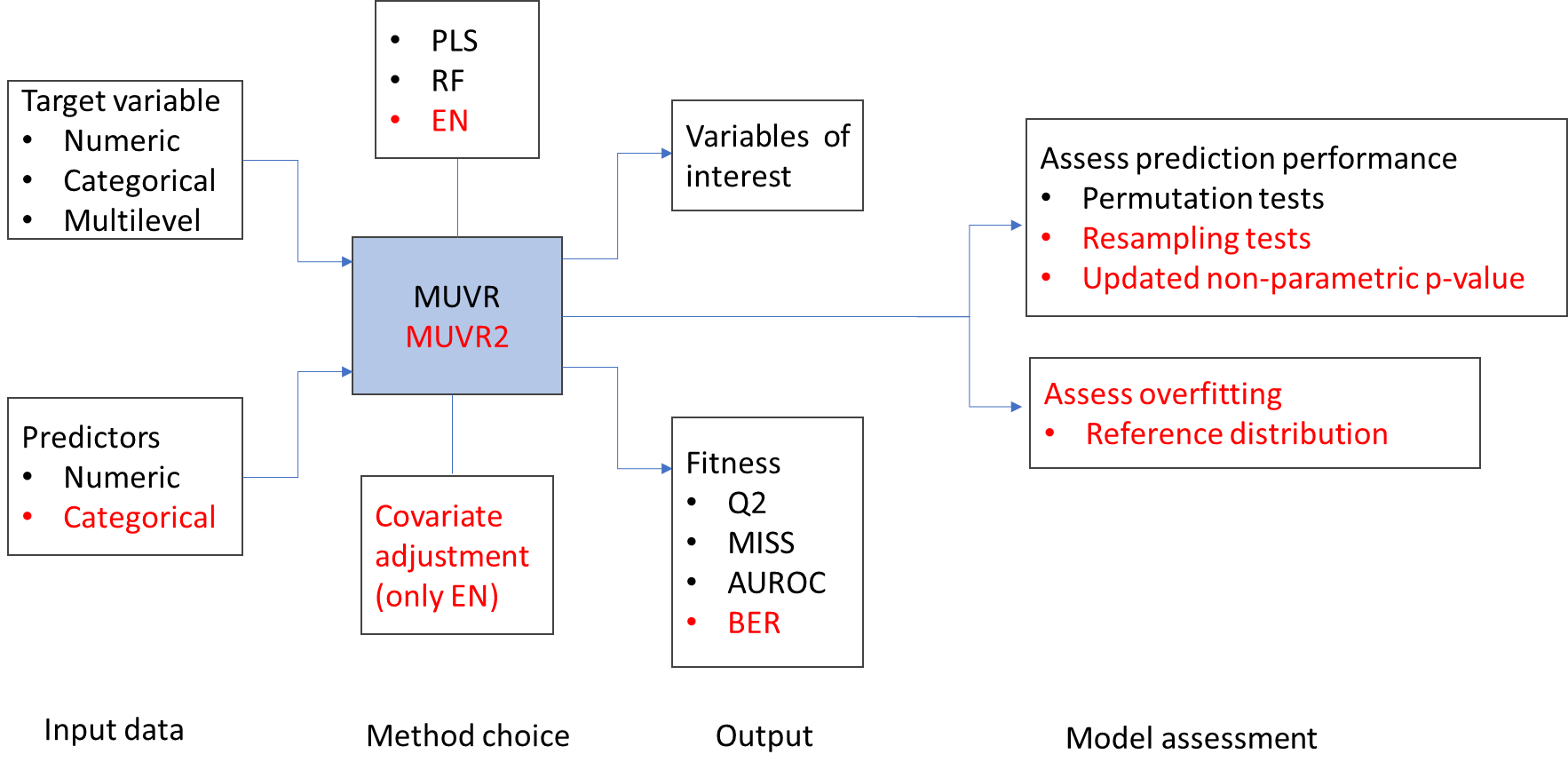


# Catalogue

1. What is MUVR2?

2. Installation

3. Preparing data

4. Analysis

4.1. Regression analysis

4.2. Classification analysis

4.3. Dealing with repeated samples

4.4. Multilevel analysis

4.5 Recap

5. Covariate adjustment

6. Resampling tests

7. Reference

# 1. What is MUVR2?

The MUVR2 package contains ­­algorithms for machine learning (ML; also known as multivariate modeling), aimed at finding associations between predictors (an X matrix of continuous and categorical variables) and a target variable (a Y vector, which is a continuous variable for *regression* or a categorical variable for *classification*). MUVR2 is particularly useful to cope with data that has large numbers of variables and few observations: MUVR2 constructs robust, parsimonious ML models that generalize well, minimize overfitting and facilitate interpretation of results through an automatic selection of variables-of-interest (Shi et al 2019). The operating principles of the MUVR2 package originate from the MUVR package (still maintained at [Carl Brunius / MUVR · GitLab](https://gitlab.com/CarlBrunius/MUVR)) (Shi et al 2019). However, the package has expanded with more functionality and also undergone some change in syntax, which warranted a breakoff into a separated R package.

From a technical perspective, MUVR2 is a statistical validation framework which incorporates minimally biased variable selection through recursive elimination performed within a repeated double cross-validation (rdCV) scheme. MUVR2 selects both minimal-optimal variables, i.e. the smallest set of highly informative variables-of-interest (useful e.g. for discovery of predictive biomarkers) and all-relevant variables, i.e. containing all variables-of-interesting that are relevant to describe the association between predictor and target variables (e.g. for biological interpretation and mechanistic investigation) (Shi et al 2019). MUVR2 supports several different data analytical problems, largely corresponding to different data types in the target and/or predictor variables: regression (Y is continuous), classification (Y is categorical) and multilevel (Y is a dummy variable and X is a so-called effect matrix, calculated from data with sample dependency, e.g. before/after or cross-over interventions). Please see additional explanations and details later in section 4.

The MUVR2 package allows elastic net (EN) core modeling in addition to partial least squares (PLS) and random forest (RF). Through this addition, covariate adjustment can now be performed in the EN modeling. We further incorporated one-hot encoding (Yu *et al.*, 2022), which entails re-coding categorical variables in the predictors onto multiple continuous variables coded as 1 or 0 for class membership. Furthermore, we upgraded the permutation test procedure into what we herein denote as resampling tests, which also include a reference distribution for assessing overfitting. The details of these functionalities are explained in section 5.

The working principle of MUVR2-PLS and MUVR2-RF modeling is visualized in **Figure 1**. The original data is randomly subdivided into ***Outer*** segments. For each outer segment, the remaining (***Inner***) data is divided into Training and Validation sets, used for tuning model parameters. This procedure is repeated for successively fewer predictor variables, achieved through recursive ranking and backwards elimination. Each ***Outer*** segment is then predicted using an optimized consensus model trained on all ***Inner*** observations, ensuring that the holdout test set was never used for training or tuning of modeling parameters. The entire procedure is then repeated to reduce stochastic effects from the sampling of data into segments, thus improving modeling stability and performance.


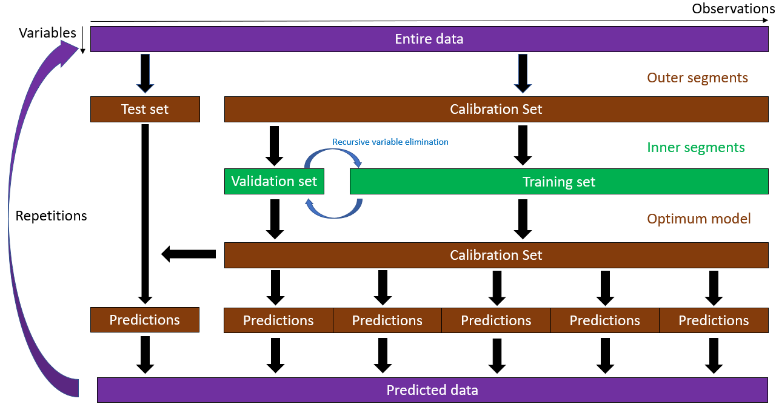

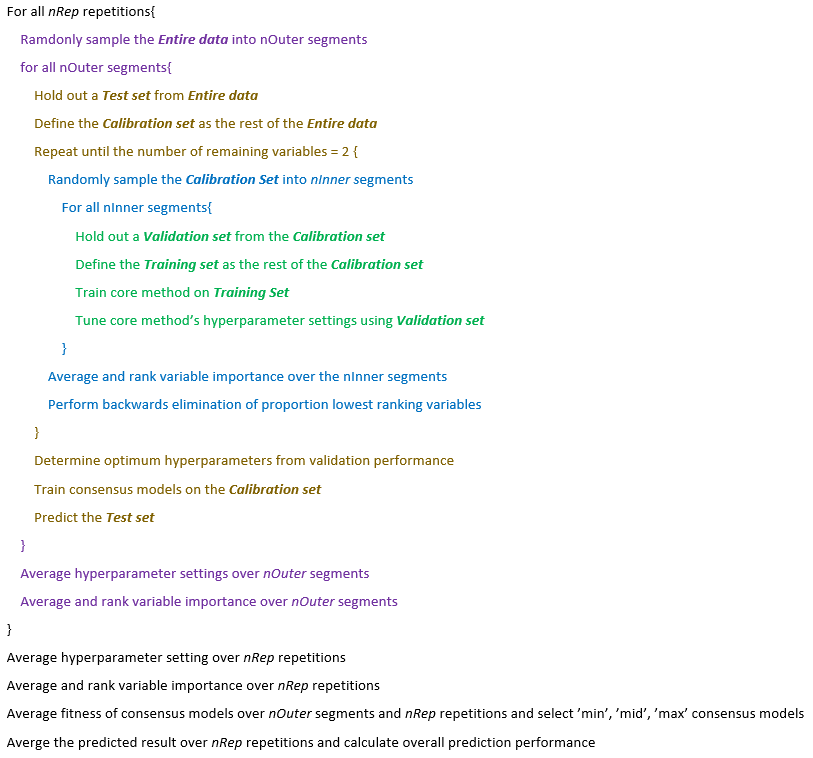


**B**
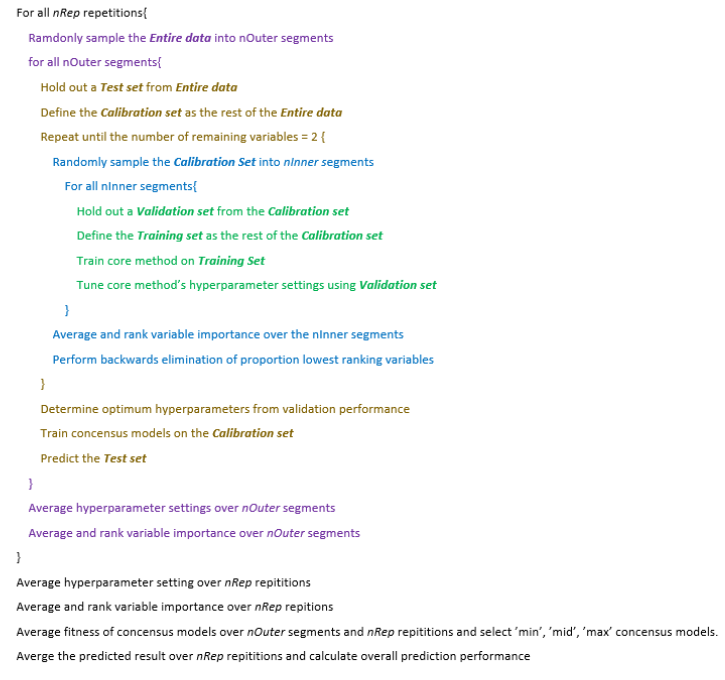


**A**
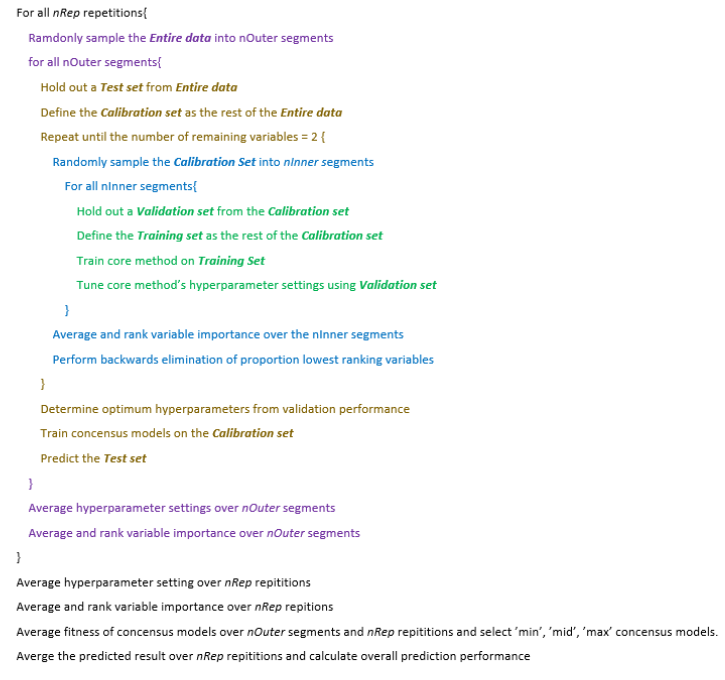


**Figure 1.** Graphical representation (A) and pseudocode (B) of the MUVR2 algorithm for PLS and RF core modeling.


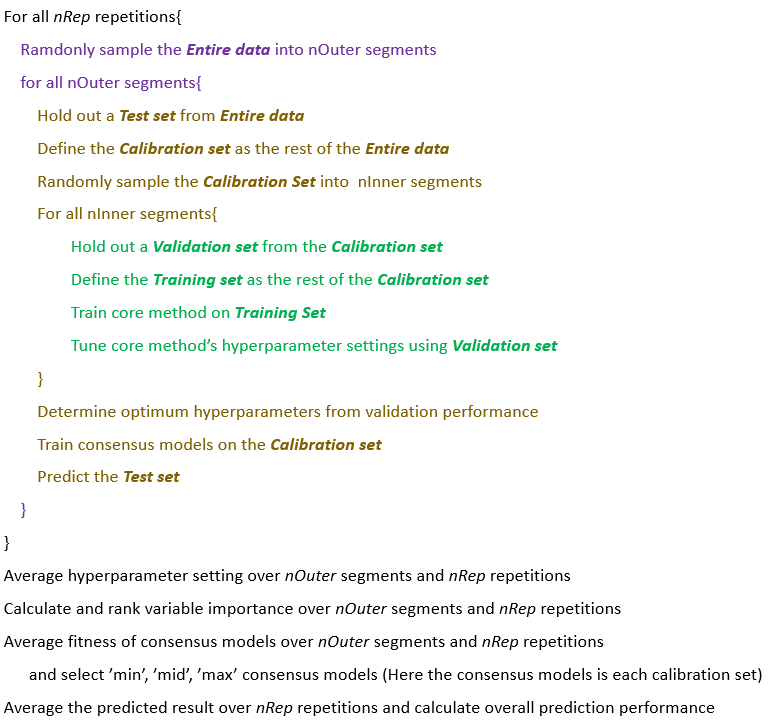
The working principle of MUVR2-EN modeling is visualized in **Figure 2**. Differing from the working principle of MUVR2-PLS and MUVR2-RF, MUVR2-EN does not perform recursive ranking and backwards elimination and thus does not perform variable selection within the model building stage (Please see section 4.2 for further details).

**B**
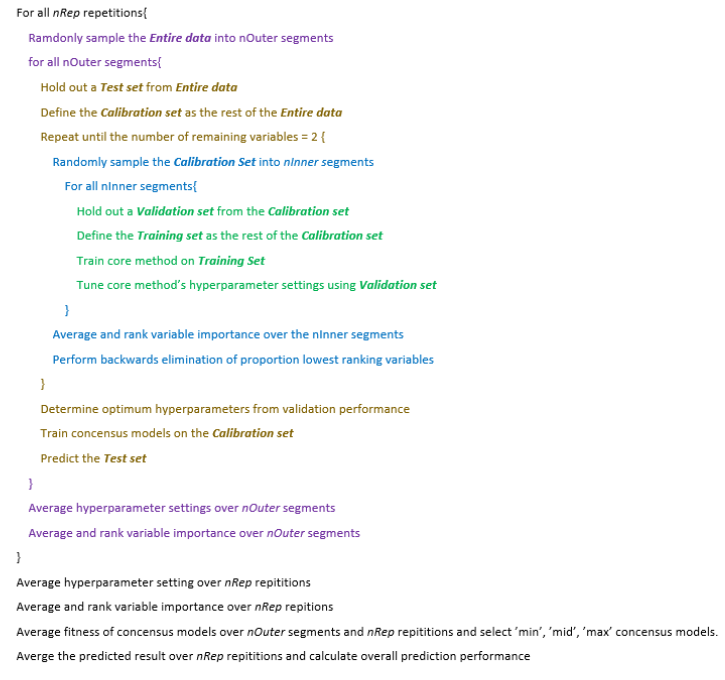


**A**
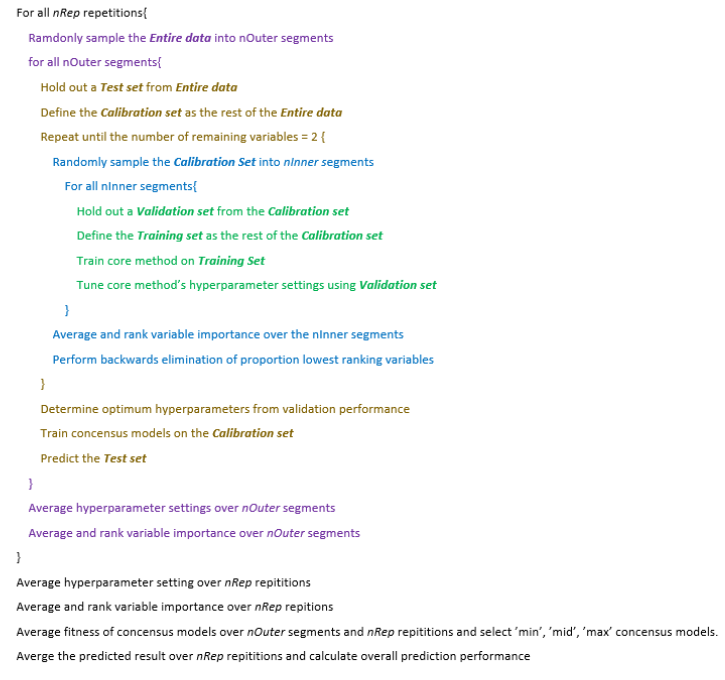


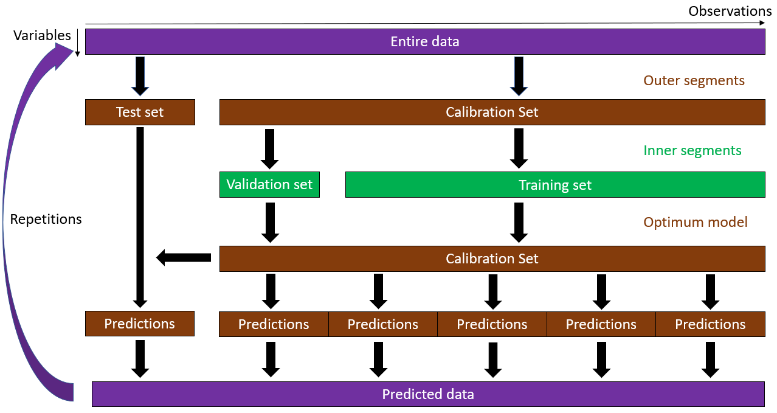


**Figure 2.** Graphical representation (A) and pseudocode (B) of the MUVR2 algorithm for EN core modeling.

# 2. Installation

First, please ensure that you have downloaded and installed R on your computer (<https://www.r-project.org/>). Furthermore, for practical data analytical work we recommend to download, install and work in RStudio (<https://www.rstudio.com/>) or another IDE of your choice, which has several advantages over working in “simple” command line R. There are several online resources for learning to work efficiently with R and RStudio (e.g. <https://www.rstudio.com/online-learning/>) and you can use any search engine to find good, freely available material. In this tutorial, R code that you may directly run in your Rstudio console is shown in red monotype font. Functions, parameters and arguments are written in *italics*.

1. Install the ‘remotes’ package from CRAN:

install.packages("remotes")

1. Install MUVR2 from Gitlab.

library(remotes)

install_github("MetaboComp/MUVR2")

1. To reduce computation time, MUVR2 uses the ‘doParallel’ package for parallel processing:

install.packages("doParallel")

# 3. Preparing data

The MUVR2 algorithm uses predictors (X) and a target (Y) data which are matched by observations, i.e. each position in the Y target variable must be matched to the corresponding row in the X matrix. The X matrix thus has observations in the rows and variables in the columns. The X columns need to have unique names (variable identifiers). This can be checked with colnames(X).

Some data types are not readily modeled by PLS without some preprocessing. For efficient analysis by PLS or EN, microbiota data may for example be log-transformed following the addition of a “pseudo count” (i.e. an offset) to manage zero values. For convenience, MUVR2 provides the function preProcess() which performs 5 different preprocessing tasks: offset (of all data); zero offset (of zero values in the data), transformation (log, sqrt or none), centering (mean, none or customized – see help file using ?preProcess) and scaling (unit variance, pareto, none or customized – see help file using ?preProcess).

The effect of predictor variables’ scaling in MUVR2 will depend on the choice of the core algorithm. For example, random forest is a scale-invariant technique and therefore insensitive to transformations such as log or sqrt, to centering and to scaling. PLS, on the other hand, is very sensitive to both transformations and scaling. The default in MUVR2-PLS is to internally center to the mean and scale data to unit variance in all underlying submodels (*scale = TRUE*). If other scaling options are desired, the user should disable automatic internal scaling (*scale = FALSE*) and pre-perform a desired preprocessing technique, e.g. using the preProcess() function.

The following tutorial sections give examples of how you can use different core modeling methods in different problems, i.e. MUVR2-PLS for regression analysis, MUVR2-EN for classification analysis and MUVR2-RF for multilevel analysis. Importantly, to avoid any confusion, we emphasize that you can use any of the 3 core modeling methods for any of the 3 problem types (regression, classification or multilevel), with or without repeated (dependent) samples.

# 4. Analysis

Please note that the MUVR2 algorithm performs resampling of the data in each repetition leading to slightly different results each time an analysis is run, wherefore results may differ slightly from those reported for the regression, classification and multilevel analysis reported here.

# 4.1. Regression analysis

In this example, we will walk through a MUVR2 regression analysis using PLS core modeling. In this regression example, we will use the “freelive2” dataset, which has data on dietary exposure to wholegrain rye and urine metabolomics data from 58 individuals to identify biomarkers of whole-grain rye exposure (Hanhineva et al 2015).

First, we set up libraries, data and parameters: Typing library(doParallel) and library(MUVR2) will call in relevant libraries and then typing data("freelive2") will load 3 objects: A continuous ‘YR2’ target variable with wholegrain rye consumption for 58 individuals; A numeric ‘XRVIP2’ matrix, consisting of 1147 metabolomics features from the samples of the 58 individuals; An ‘IDR2’ vector with unique identifiers of each individual (numerical IDR2). Note that we will discuss the scenario of repeated samples later in section 4.4, where each individual could have more than one observation, representing multiple samples from the same individual.

# Call in relevant libraries
library(doParallel) # Parallel processing
library(MUVR2) # Machine Learning modeling

# Call in the "freelive2" data from the MUVR2 package
data("freelive2")

It is often practical to separate between *nCore* (i.e. the number of computer cores to use for processing) and *nRep* (the number of repetitions in the MUVR2 algorithm). Performing nCore <- detectCores() - 1 uses all but one thread (kept for everyday computer usage), which makes for efficient processor use. Performing instead nCore <- detectCores() will result in slightly more efficient CPU usage for calculations, but leave you with practically no possibility to use your computer for other tasks during calculations. *nRep* is usually set to a multiple of *nCore* for efficient processor usage. For initial “quick’n’dirty” modeling (as in all the examples we show below), we normally set *nRep = nCore*. For final processing, we often set *nRep* between 20 and 50 and check modeling convergence using the plotStability() function (see below). Depending on your parameter settings and size of data, the modeling may take minutes or even hours. Initial modeling using “quick’n’dirty” settings is thus encouraged to get a feel for the modeling potential before final processing.

We normally set the number of outer cross-validation segments between 6-8, with higher number of segments when there are fewer observations to increase the ratio of observations used for model training. A general recommendation is also to ensure that all classes are present in all segments (by ensuring that *nOuter* is not larger than the smallest class size within the target variable). The variable ratio (*varRatio*) parameter governs the proportion of variables kept for iteration of the recursive variable elimination in the inner loop. We normally start out low (*varRatio* = 0.6-0.75) and increase towards 0.8-0.9 for final processing.

# Set method parameters
nCore <- detectCores() - 1 # Number of processor threads to use
nRep <- nCore # Number of MUVR2 repetitions
nOuter <- 8 # Number of outer cross-validation segments
varRatio <- 0.75 # Proportion of variables kept per iteration
method <- 'PLS' # Selected core modeling algorithm

After setting parameters, it is time to initialize parallel processing and perform the actual modeling.

# Set up parallel processing
cl <- makeCluster(nCore)
registerDoParallel(cl)

# Perform modeling

regrModel <- MUVR2(X = XRVIP2,

Y = YR2,

nRep = nRep,

nOuter = nOuter,

varRatio = varRatio,

method = method)

# 1.49 mins using 7 threads on a laptop computer with 11th Gen Intel I Core I i7 -1185G7 processor with 8 cores and 31.7 Gb internal memory.

# Stop parallel processing
stopCluster(cl)

After the model finishes running, we can check the prediction performance, hyperparameter selection and number of variables selected by ‘min’, ‘mid’, ‘max’ consensus models (see below).

regrModel$fitMetric # Look at fitness metrics for min, mid and max models
# $R2
# [1] 0.9196686 0.9317392 0.9359603
# $Q2
# [1] 0.4311033 0.4140635 0.4633704

regrModel$nComp # Number of components for min, mid and max models
# min mid max
# 3 3 3

regrModel$nVar # Number of variables for min, mid and max models
# min mid max
# 47 85 152

cbind(YR2, regrModel$yPred) # Actual exposures side-by-side with min, mid and max predictions
# YR2 min mid max

# 1_ID1 11.0666667 22.329490 21.730449 23.196849

# 2_ID100 12.1000000 8.743642 7.655014 8.654301

# 3_ID101 57.7333333 64.920179 78.548436 82.403806

# … … … … …

In the validation plot generated by plotVAL(regrModel)(**Figure 3**), light grey lines represent validation performance for the individual *inner* segments as a function of the number of variables in the recursive variable elimination procedure. The darker grey lines represent inner segment validation curves averaged over each repetition. Similarly, the black line represents validation curves averaged over all repetitions. The “quick’n’dirty” model with few repetitions (Figure 3A) and the final model (Figure 3B) show similar validation trends, although with improved resolution in the final model.

Minimal-optimal (‘min’) and all-relevant (‘max’) models represent the outer borders of variable selections where the validation performance is optimal (in this case represented by root mean squared error of prediction (RMSEP)). This is in practice determined as having validation performance within a certain percentage of slack allowance from the actual minimum (default 5%), when rescaling the fitness range from the worst to the best fitness to {0, 1}. The minimal-optimal model thus represents the minimal variable set required for optimal method performance, i.e. with the strongest predictors e.g. suitable for biomarker discovery. The all-relevant model instead represents the data set with all variables with relevant signal-to-noise in relation to the research question: i.e. the strongest predictors and, additionally, variables with redundant but not erroneous information. The ‘mid’ model represents a trade-off between the ‘min’ and ‘max’ model and is found at the geometric mean.

plotVAL(regrModel)


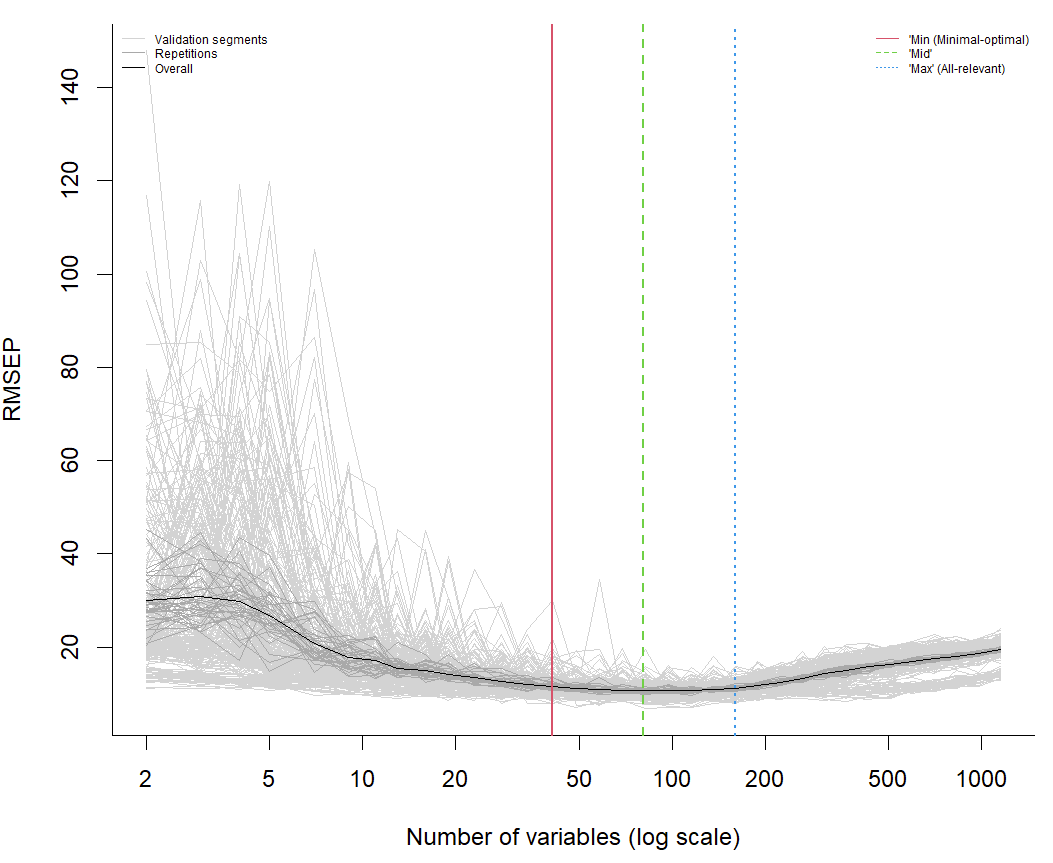

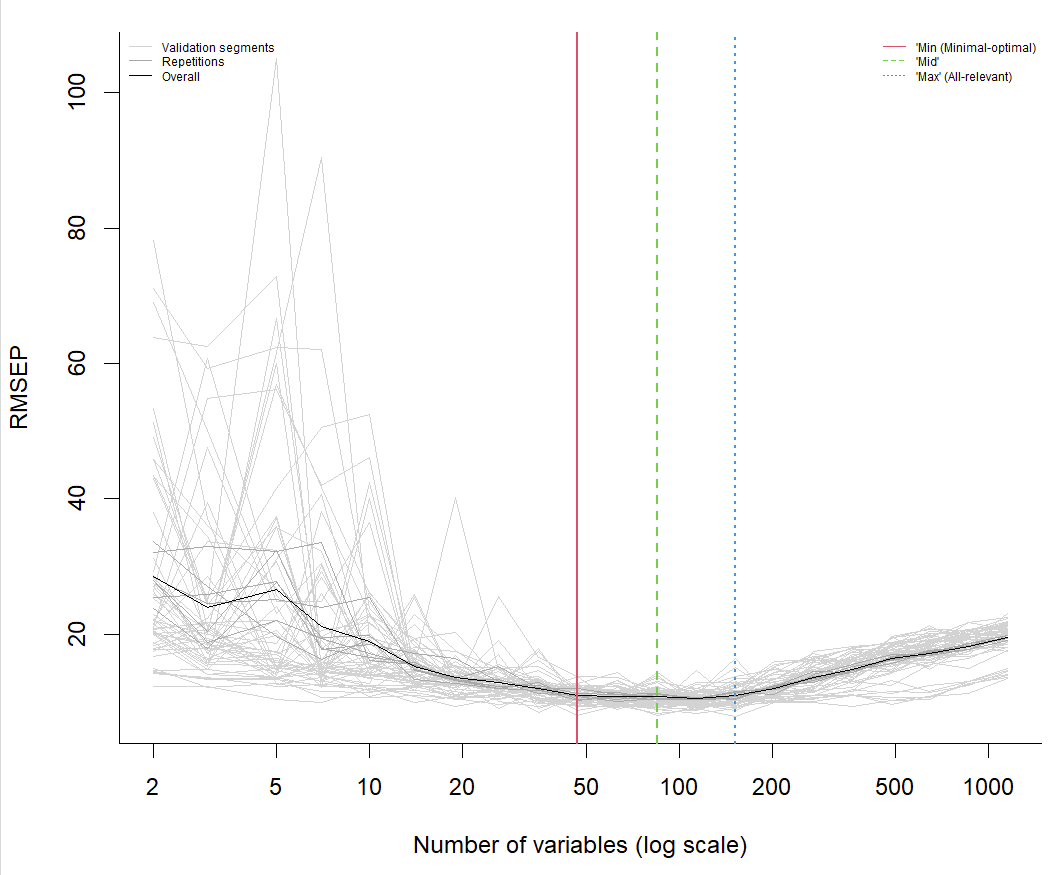


**B**
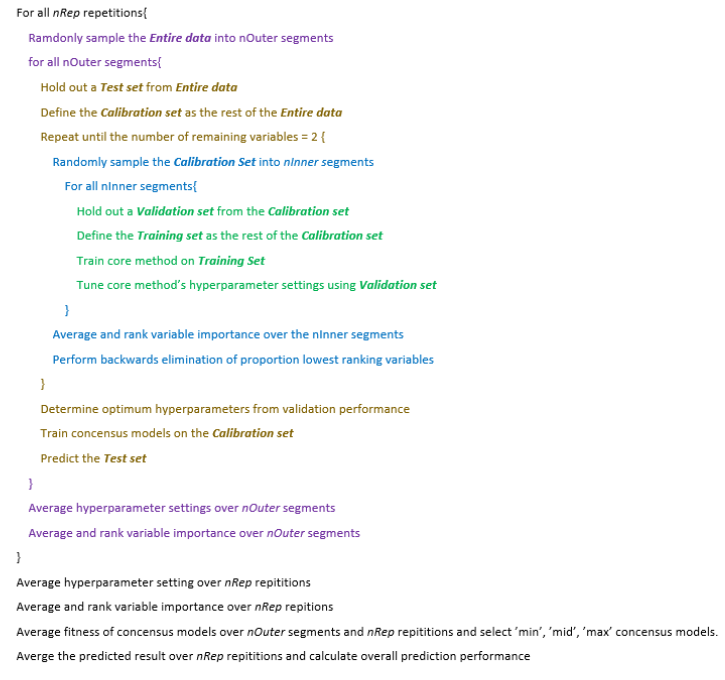


**A**
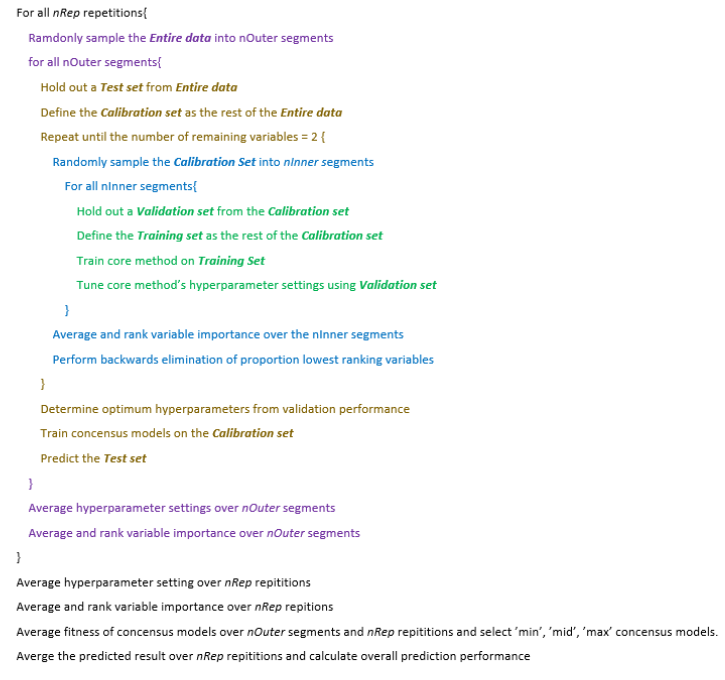


**Figure 3**. Validation plots of MUVR2-PLS models in the regression analysis using different settings, plotted by *plotVAL()*. The left figure (A) uses *nRep = 7, varRatio = 0.75, nOuter = 8* and the right figure (B) uses *nRep = 35, varRatio = 0.85, nOuter = 8*.

For regression analysis, the *plotMV()* function plots the actual target variable on the x-axis and predictions on the y-axis (**Figure 4**). Predictions from individual repetitions are represented as smaller, grey dots, whereas overall predictions are represented by larger black dots. This provides an overview of prediction precision between repetitions. Inlaid is validation R^2^ obtained from overall consensus model (i.e. a model using all data for training and prediction, but with the number of components and variable selections obtained from MUVR2) and Q^2^ obtained from MUVR2 modeling.

For convenience, the MUVR2 package also provides a PLS biplot function, *biplotPLS()* for visual interpretation of PLS models by giving an overview of observation scores and variable loadings (**Figure 5)**. In the present case, the symbols are color coded (grey scale) according to their exposure (*xCol = YR2*). To avoid cluttering, score and loading labels were omitted.

**Figure 4**. Comparison between actual and predicted target variable of MUVR2-PLS in the regression analysis, plotted by *plotMV()*

plotMV(regrModel, model = 'min') # Look at the model of choice: min, mid or max
PLSfit = regrModel$Fit$plsFitMin # Extract consensus PLS model


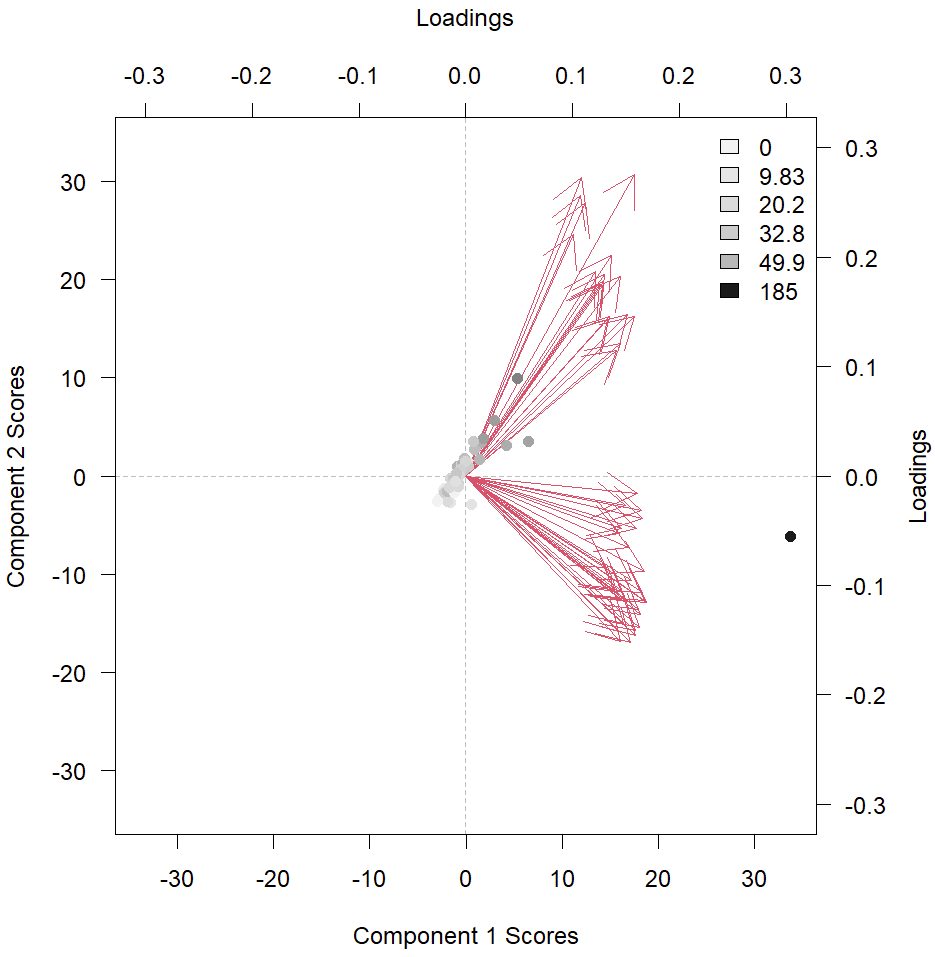

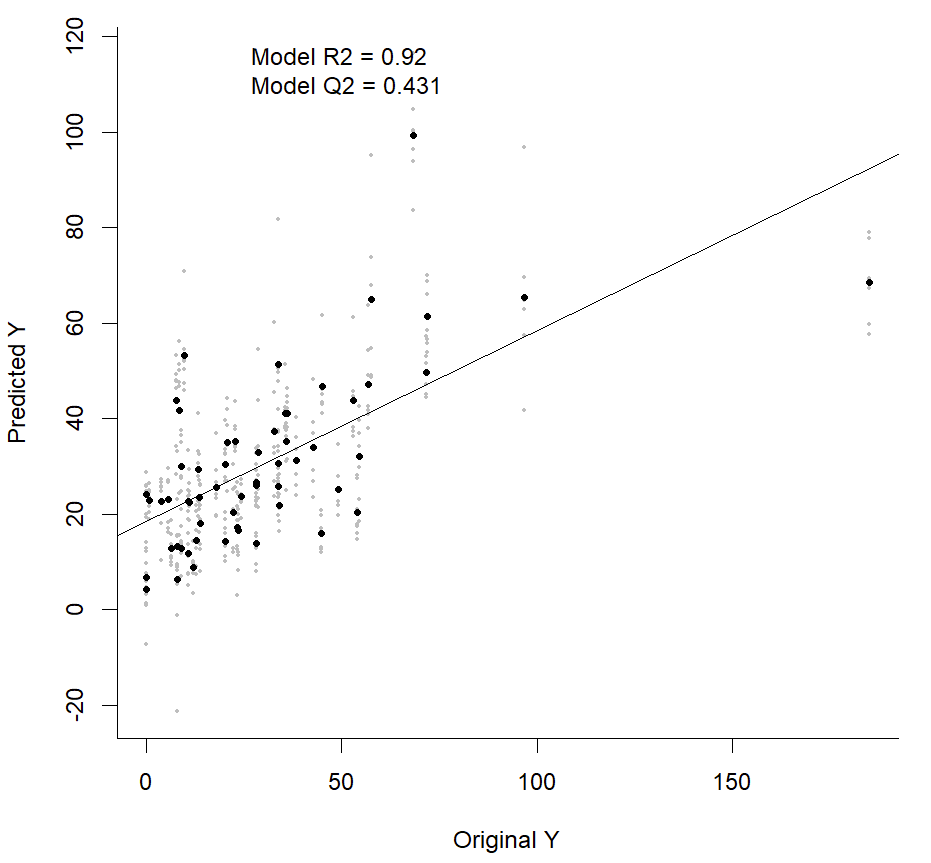
biplotPLS(PLSfit, comps = 1:2, xCol = YR2, labPlSc = FALSE, labPlLo = FALSE)

**Figure 5.** Scores and loadings of variables selected by the minimal-optimal consensus model of MUVR2-PLS in the regression analysis, plotted by *biplotPLS()*

The stability plot from *plotStability()* for regression analysis generates three subplots (**Figure 6**): (i) The number of selected variables in each repetition, as well as the cumulative average over the repetitions; (ii) The proportion of selected variables shows the ratio of the final variable selection found in each repetition and cumulatively, averaged over the number of repetitions; (iii) Q^2^ per repetition and cumulatively. For all subplots, there may be some variability between individual repetitions due to the random sampling of observations into the cross-validation segments. However, cumulative averages converge rapidly, and we normally observe convergence within 20-50 observations or even faster, depending on the strength of the association between predictor and target variables. If the stability plot shows high variability, especially for the cumulative curves, a higher *nRep* argument setting may be warranted.

plotStability(regrModel, model = 'min')


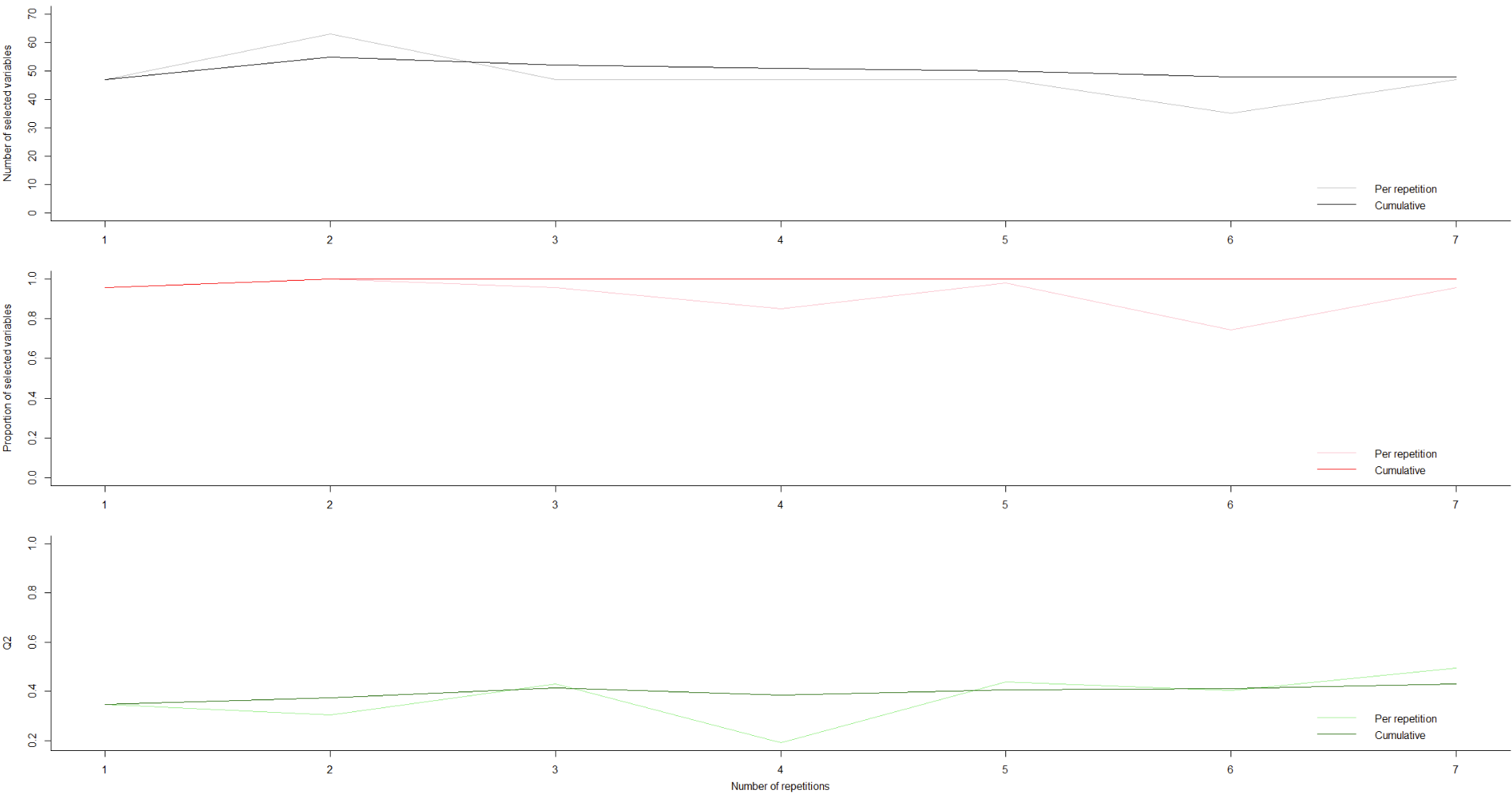


**Figure 6**. Stability plots under the minimal-optimal consensus model of MUVR2-PLS in the regression analysis, plotted by *plotStability().*

The *plotVIRank()* function generates a boxplot of the ranks-per-repetition (lower is better) of a number of top-ranked variables (argument *n* specific the number of variables) (**Figure 7**) or the variables that are automatically selected (argument *model = ‘min’*, *‘mid’* or *‘max’*). This output generates an intuitive overview of which variables are reproducibly selected with low rank, which may thus be the strongest predictors of the target variable. For the sake of readability of variable names, the following VIRank plot was truncated to showing the top 20 predictors. However, the default is to show the number of variables corresponding to either the ‘min’, ‘mid’ or ‘max’ selection.

plotVIRank(regrModel, n = 20)


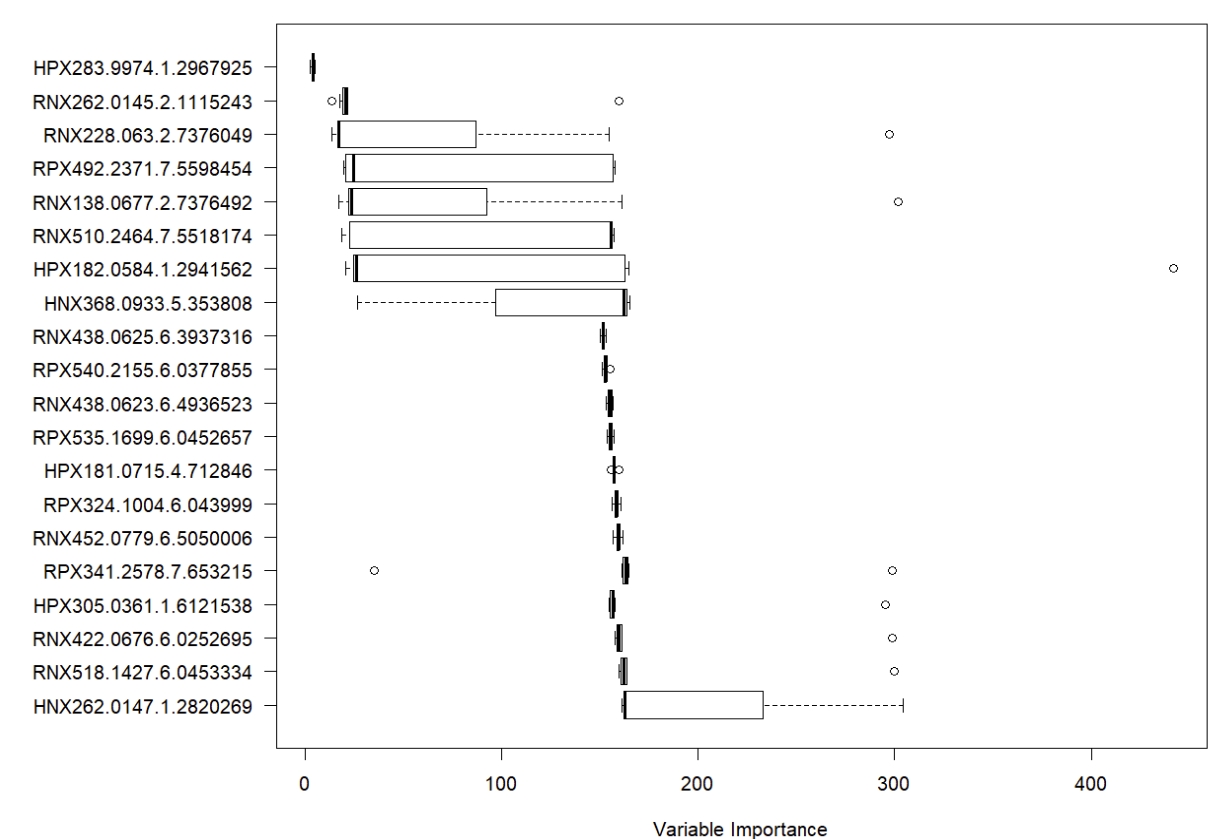


**Figure 7**. Variable importance ranks under the minimal-optimal consensus model of MUVR2-PLS in the regression analysis, plotted by *plotVIRank()*. Truncated to top 20 variables for the sake of readability of variable names.

The selected variables can also be obtained using *getVIRank()*:

getVIRank(regrModel, model = 'min') # Extract most informative variables: Lower rank is better
# order name rank

# HPX283.9974.1.2967925 1 HPX283.9974.1.2967925 3.903061

# RNX262.0145.2.1115243 2 RNX262.0145.2.1115243 39.155612

# RNX228.063.2.7376049 3 RNX228.063.2.7376049 76.359694

# RPX492.2371.7.5598454 4 RPX492.2371.7.5598454 79.339286

# ...

Note that regression using Random forest can easily be achieved by changing the ‘method’ argument into *method = ”RF”* and regression using elastic net will be achieved by the *MUVR2_EN()* function (see section 4.2).

# 4.2. Classification analysis

The general outline of a classification analysis follows the same approach as the regression analysis described above, including considerations for parameter settings, albeit with the difference that the Y target variable consists of factor levels rather than numeric values. We will walk through a MUVR2 classification analysis using elastic net (EN) core modeling. We will use the “mosquito” dataset, which has data on microbiota composition data (OTU data from 16S rRNA analysis) from 29 *Anopheles gambiae* mosquitoes sampled from 3 different villages in western Burkina Faso (Buck et al 2016).

First, we set up libraries, data and parameters as in section 4.1: We call in relevant libraries and typing data("mosquito") will load 2 objects: A categorical ‘Yotu’ target variable (three levels, i.e. villages) for 29 samples and; A numeric ‘Xotu’ matrix, consisting of 1678 16S rRNA operational taxonomic units (OTU) measured for the 29 samples.

# Call in relevant libraries
library(doParallel) # Parallel processing
library(MUVR2) # Machine Learning modeling

# Call in the "mosquito" data from the MUVR2 package
data("mosquito")

We then check for the number of observations per class to make sure *nOuter* is not bigger than the number of observations of the smallest class.

# Check the number of observations per class
table(Yotu) # As a general principle, nOuter should be ≤ n of the smallest class (i.e. 8)

# Yotu

# VK3 VK5 VK7

# 10 11 8

The elastic net in the MUVR2 algorithm is slightly different from PLS and RF. First, we use the *MUVR2_EN()* function instead of *MUVR2()* to perform the modeling and we do not need to specify a ‘method’ since *MUVR2_EN()* only performs elastic net (EN) core modeling. Second, EN core modeling in MUVR2 does not perform the variable selection by recursive variable elimination similar to MUVR2-PLS and MUVR2-RF. Instead, it ranks variables by the proportion of times that variables had a non-zero beta coefficient across the calibration set consensus models (i.e. over a total of *nOuter* x *nRep* models). Variables that had non-zero beta coefficients more frequently are ranked as more important. Finally, *MUVR2_EN()* does not report the selected number of variables directly since the variable selection requires some manual assessment (see below).

We can now set parameters, initialise parallel processing and perform the actual modeling. Note that to do a classification, you should specify the argument *DA = TRUE*, where DA stands for discriminant analysis. However, for convenience, the DA argument can be omitted and *MUVR2()* or *MUVR2_EN()* will automatically perform a classification analysis if the Y vector is either in factor or character format (or a regression analysis if the vector Y is in numeric format).

# Set method parameters
nRep <- 35 # Number of MUVR2 repetitions
nOuter <- 8 # Number of outer cross-validation segments

# Set up parallel processing using doParallel

nCore <- detectCores() - 1 # Number of processor threads to use
cl <- makeCluster(nCore)
registerDoParallel(cl)

# Perform modeling (It may take some time to run).
classModel <- MUVR2_EN(X = Xotu,

Y = Yotu,

nRep = nRep,

nOuter = nOuter,

varRatio = varRatio,

DA = TRUE)

# Stop parallel processing
stopCluster(cl)

The reason *nVar* is not reported using *MUVR2_EN()* is that variables are not selected from recursive elimination as in MUVR2-PLS and MUVR2-RF. There are thus no continuous validation curves from which to estimate prediction performance. Instead, there are two options for variable selection in MUVR2-EN, using the *getVar()* function: In the first approach (*option = ”fitness”*), an analogous validation curve is generated from the smoothing of the *nRep* x *nOuter* prediction performances for each *test set.* Using this approach, the ‘min’, ‘mid’ and ‘max’ selections are decided similarly as for MUVR2-PLS and MUVR2-RF (i.e. validation performance within a certain percentage of slack allowance from the actual minimum (default *robust* = *5%*).

However, this dependence on individual data points rather than continuous curves may result in the smoothed validation curve having uneven coverage over the range of selected variables. Users should therefore first visualize the validation curve using *plotVAL()*. For example, in **Figure 8A**, the validation plot (*nRep = 35, nOuter = 8*) shows that the ‘min’, ‘mid’ and ‘max’ selections all reside in places where the density of data points is not the highest. However, although it is obvious that higher numbers of selected variables are under-represented, the validation curve from the model above with the 280 underlying fitness estimates looks as expected with a well-defined global minimum (U-shape). Additionally, in some scenarios, the smoothed curve may show strange behavior, not conforming to the expected U-shape. For example, a corresponding “quick’n’dirty” model (*nRep = 5, nOuter = 5*) for the same data clearly does not produce a similar U shape and does not have a sufficient amount of fitness estimates to serve as grounds for producing a reliable validation curve (**Figure 8B**). This suggests that the number of repetitions (and possibly *nOuter* segments) should be increased.

classModel <- getVar(classModel,

option = "fitness",

robust = 0.05,

outlier = "none")

**
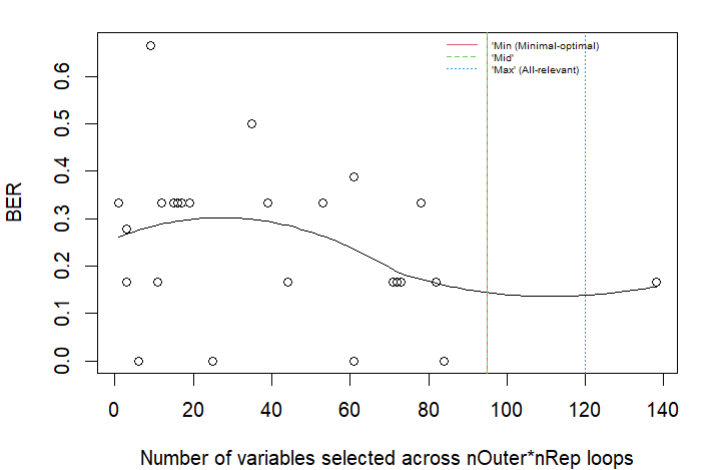

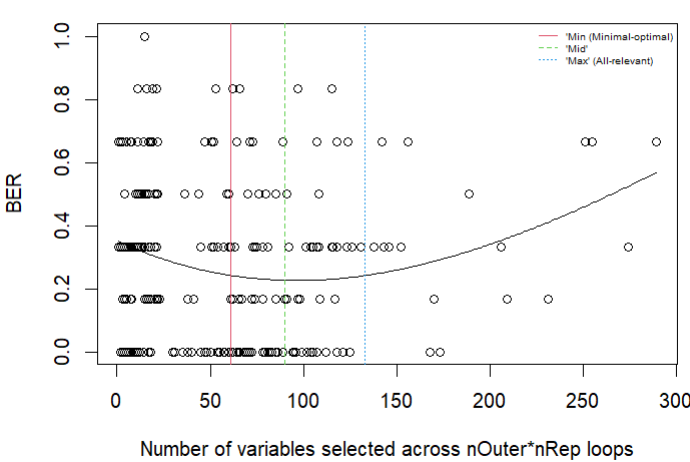
**plotVAL(classModel)

**A**
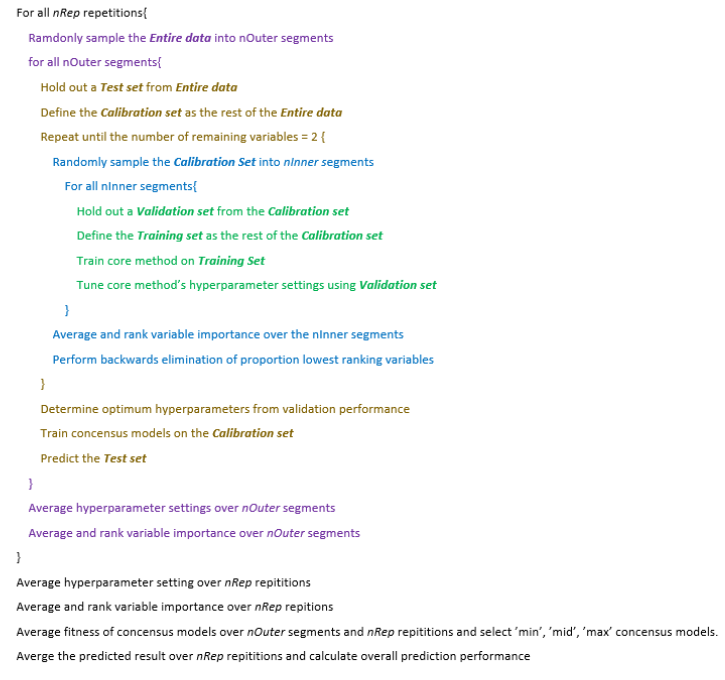


**B**
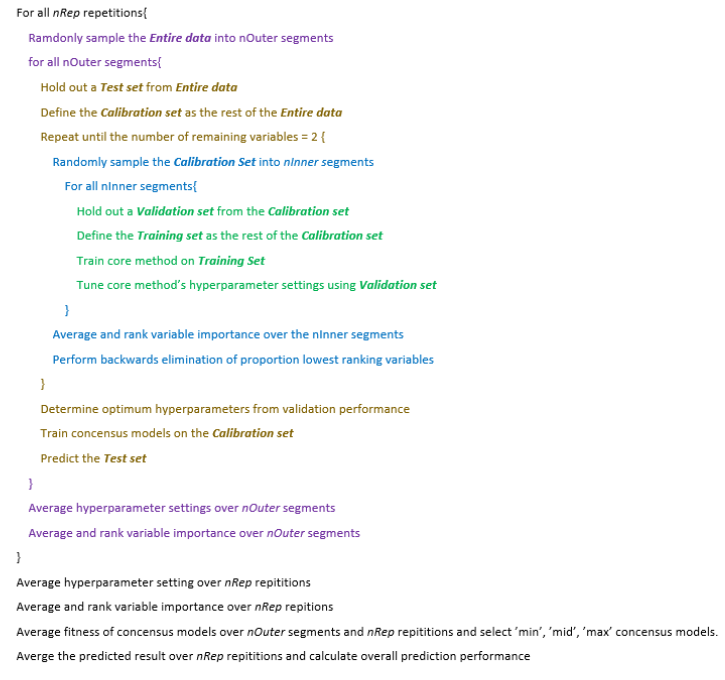


**Figure 8**. Validation plots of MUVR2-EN models using the smooth curve method for variable selection. The left figure (A) uses *nRep = 35, nOuter = 8* and the right figure (B) uses *nRep = 5, nOuter = 5*.

To further explore the ‘min’, ‘mid’ and ‘max’ selections, users can rerun *getVar()* with different allowances for validation performance (changing the *robust* argument from its default 0.05). In addition, the curve smoothing is sensitive to outliers, which can result in “squiggly” curve shapes. In this case, users can both choose to automatically detect and remove outliers using either interquartile range or the residuals from generalized additive models (specifying the argument *outlier = “IQR” or outlier = ”residual”* rather than its default *outlier =“none”*) and also adjust the amount of smoothing (decreasing the *span* argument from its default 1) in *getVar().*

If well-defined validation curves can still not be achieved by increasing the number of repetitions or *nOuter*, by removing outliers or modifying the degree of smoothing, this first approach is not viable and we instead suggest a second approach for selecting variables, using the argument *option = ”quantile”.* Contrary to the standard MUVR2 approach and the above described "smoothed" approach, this method disregards the modeling performance and instead selects ‘min’, ‘mid’ and ‘max’ directly from the distribution of the number of selected variabels (i.e. non-zero beta coefficients) across the *nRep* *x nOuter* calibration set models. The ‘mid’ option always corresponds to the median, whereas the argument *quantile* allows the user to choose the number of selected variables for ‘min’ and ‘max’ based on quantiles. For example, based on the same modeling result as **Figure 8A**, **Figure 9** used *option = ”quantile”, quantile = 0.25* in *getVar(),* which gives the first quartile as the ‘min’, median as the ‘mid’ and the third quartile as the ‘max’ number of selected variables. This concentrates the variable selections to the positions where the density of the number of variables selected by *nRep* *x nOuter* calibration set models is the highest although at the cost of not simultaneously incorporating overall prediction performance in the assessment.

classModel <- getVar(classModel,

option = "quantile",

quantile = 0.25,

outlier = "none")

plotVAL(classModel)


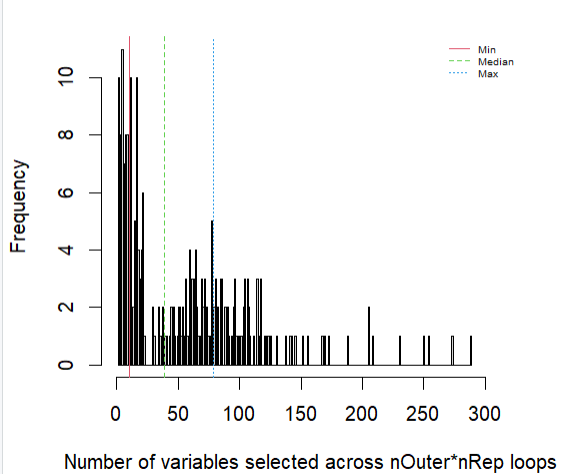


**Figure 9**. Validation plots of MUVR2-EN models using the quantile method for variable selection (based on the same modeling result as **Figure 8A)**.

We can then look at the final model outputs, similar to the previous regression example. However, note that classification analysis uses different fitness metrics than regression (with balanced error rate (BER) as default). An important difference in MUVR2-EN compared to MUVR2-PLS and MUVR2-RF is that we only have one BER and one predicted target variable for the model instead of one each for ‘min’ ‘mid’ and ‘max’. This is because there is no recursive variable elimination for MUVR2-EN and *nVar* is selected after model building.

classModel$ber # Balanced error rate for min, mid and max models

# 0.1356061

classModel$nVar # Number of variables for min, mid and max models

# min mid max

# 10 39  79

cbind.data.frame(Yotu, classModel$yClass) # Actual class side-by-side with min, mid and max predictions

# Yotu classModel2$yClass

# 1 VK3 VK3

# 2 VK3 VK3

# 3 VK3 VK3

# 4 VK3 VK3

# … … …

classModel$varTable

# Number of times a variable is being selected across nRep*nOuter calibration set models

# OTU_28 OTU_4133 OTU_400 OTU_1620 OTU_1013 OTU_243 OTU_134 OTU_337 OTU_26 OTU_1624 OTU_1679 …

# 280 263 225 224 223 210 191 189 184 179 178 …

The prediction plot for classification analysis is different from its regression counterpart. The *plotMV()* function generates what we call a “swim lane” plot, where each lane represents predictions for an observation (**Figure 10**). Predictions are color-coded and jittered by class. The smaller dots represent predictions from individual repetitions and the larger dots represent class prediction averaged over all repetitions. Misclassified predictions are circled. The spread in the individual predictions gives an intuitive graphical overview of prediction precision. In MUVR2-PLS and MUVR2-RF, using the *model* argument, the users can easily switch between ‘min’, ‘mid’ and ‘max’ models.

plotMV(classModel)

# In MUVR2-PLS and MUVR2-RF, you can specify min, mid or max using the ‘model’ argument.


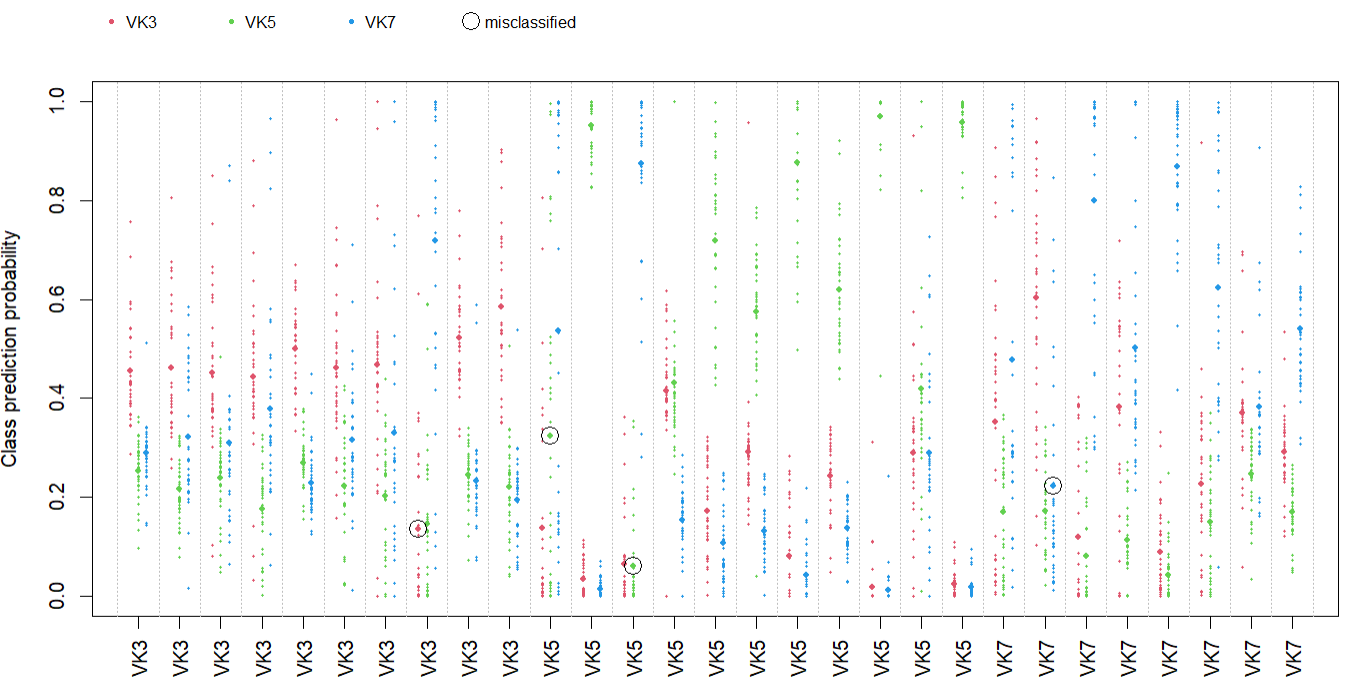


**Figure 10**. Class prediction probability for observations of MUVR2-EN in the classification analysis, plotted by *plotMV()*

The stability plot now gives misclassifications and BER instead of Q^2^ in the bottom part of the plot (**Figure 11**), but is otherwise similar to the regression stability plot.

plotStability(classModel, model = 'mid')


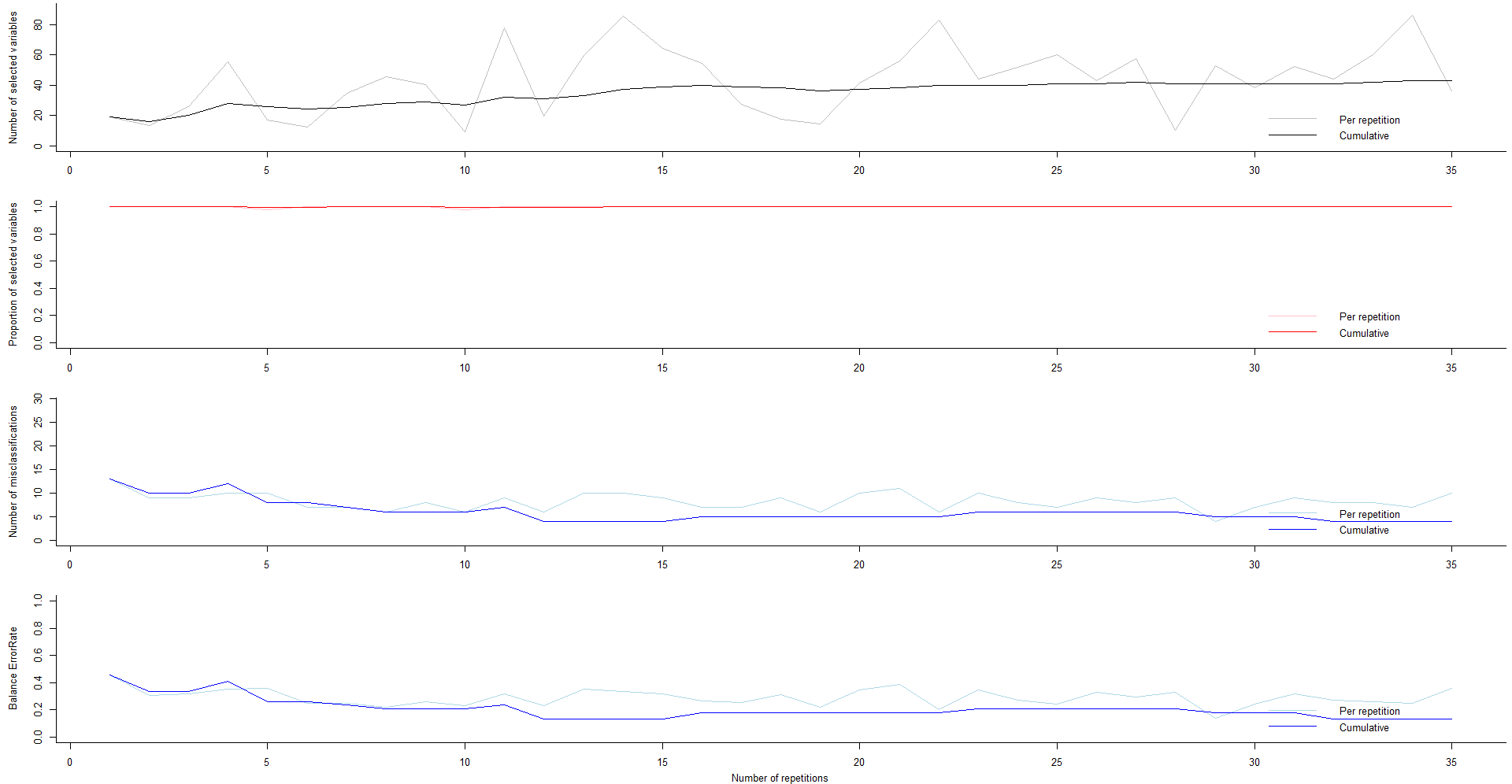


**Figure 11**. Stability plots under the 'mid' variable selection of MUVR2-EN in the classification analysis, plotted by *plotStability().*

Selected variables can be obtained similar to the regression analysis using *plotVIRank()* and *getVIRank()*. Note that *plotVIRank()* in MUVR2-EN gives a different plot than MUVR2-PLS and MUVR2-RF, since variable selection in MUVR2-EN is based on the variables that have non-zero beta-coefficient over *nOuter* segments and *nRep* repetitions rather than recursive variable elimination. In **Figure 12**, each row represents one calibration set model (*nRep* x *nOuter* models) and each column represents a variable. Red denotes that the variable was selected (i.e. had a non-zero beta coefficient) in the corresponding calibration set model. Calibration set models are ordered from selecting the most to the fewest variables and variables are sorted based on selection rates. Despite a wide variability in the number of selected variables, a core set of variables was systematically selected.

plotVIRank(classModel, model = 'mid', maptype = "heatmap")


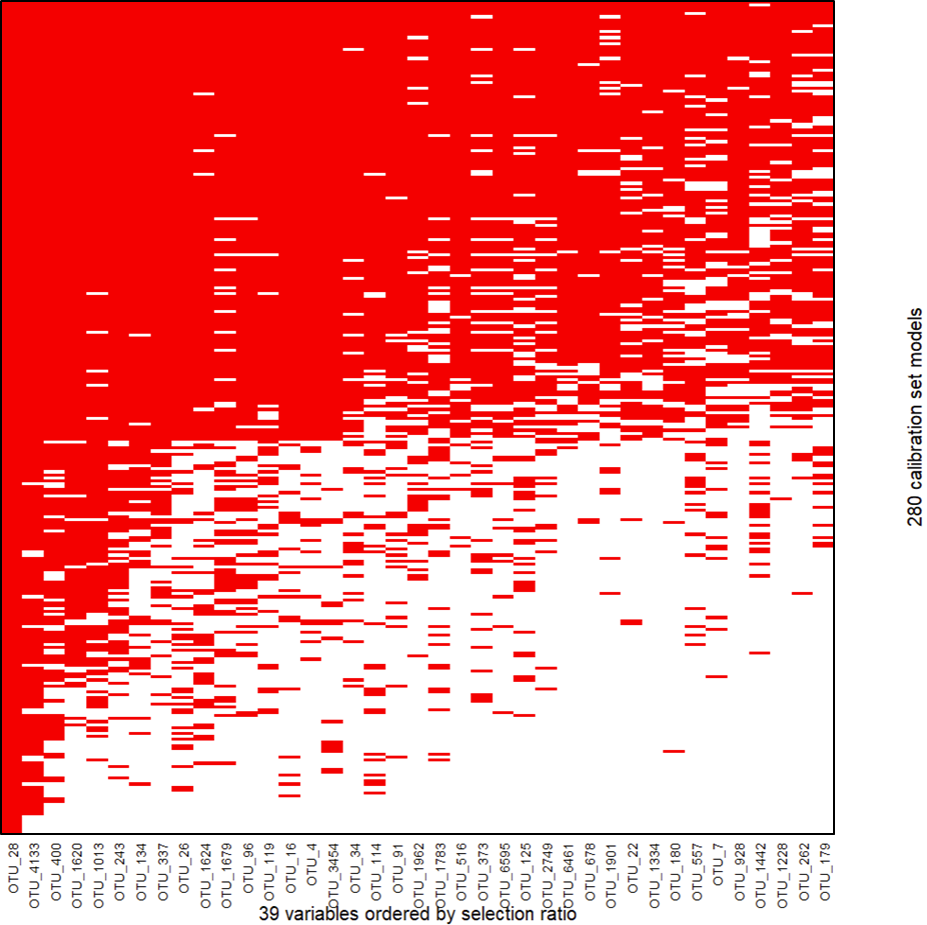


**Figure 12**. Variable importance ranks under the 'mid' variable selection of MUVR2-EN in the classification analysis, plotted by *plotVIRank()*

getVIRank(classModel, model = 'mid') # Extract most informative variables: Lower rank is better
# order name rank
# OTU_28 1 OTU_28 1.0

# OTU_4133 2 OTU_4133 2.0

# OTU_1620 3 OTU_1620 3.0

# OTU_400 4 OTU_400 4.0
# … … … …

# 4.3. Dealing with repeated samples

In the regression example in section 4.1, we have used the ”freelive2” data, where the observations are from 58 different individuals, i.e. with one sample per individual. What if there are more than one sample for some individuals in the data? Standard procedures for cross-validation will not take the this into account during the division of data into cross-validation segments (folds). Consequently, there is a high likelihood of overfitting to the data by having observations from the same individual present in both model training, validation and/or testing segments. To reduce such overfitting, *MUVR2()* and *MUVR2_EN()* can use the *ID* argument to ensure that samples from the same individual are always kept together in the cross-validation segments.

An example is given here to add complexity of repeated samples using the “freelive” data, which is similar to “freelive2” data but including more than one sample per individual. First, we set up libraries, data and parameters as in section 4.1: We call in relevant libraries and typing data("freelive") will load 3 objects: A continuous ‘YR’ target variable with wholegrain rye consumption for 112 samples from 58 individuals (some individuals did not provide repeated samples); A numeric ‘XRVIP’ matrix, consisting of 1147 metabolic features for the 112 samples; An ‘IDR’ vector with identifiers of the individuals that informs which samples are from which individual.

To ensure that all observations per individual are co-sampled into the same cross-validations segment, we can simply add the ID argument to the *MUVR2()* function call from section 4.1.

# Set method parameters
nCore <- detectCores() - 1 # Number of processor threads to use
nRep <- nCore # Number of MUVR2 repetitions
nOuter <- 8 # Number of outer cross-validation segments
varRatio <- 0.75 # Proportion of variables kept per iteration
method <- 'PLS' # Selected core modeling algorithm

cl <- makeCluster(nCore)
registerDoParallel(cl)

regrModel <- MUVR2(X = XRVIP,

Y = YR,

ID = IDR,

nRep = nRep,

nOuter = nOuter,

varRatio = varRatio,

method = method)

stopCluster(cl)

# 4.4. Multilevel analysis

In the example in section 4.3, there was a dependency between observations due to repeated sampling. However, this represented to measurement occasions, both under free-living conditions. Since it was not related to a systematic effect (such as before-vs-after intervention or treatment A-vs-B in a crossover design), the dependency was managed by co-sampling dependent observations into the same cross-validation segments using the ID argument. However, sample dependency may also be related to such a systematic effect. Analysing such data using standard classification modeling will result in both (i) having observations from the same individual both in model training and testing if not ensuring co-sampling (as in the previous example using the ID argument) and, in addition, (ii) conflation of between-individual effect and treatment-related systematic within-individual effects. This sample dependency by experimental unit needs to be addressed in a different manner, e.g. by so-called multilevel ML analysis (Westerhuis et al, 2010). This should, however, not be confused with the multilevel model concept in classical statistics (<https://en.wikipedia.org/wiki/Multilevel_model>).

In the rationale building up to this approach, a standard machine learning-based classification analysis can be viewed as a multivariate extension of an unpaired t-test, i.e., comparing systematic differences between two groups although analysing multiple predictor variables simultaneously. Using the same analogy, a multilevel model would correspond to a paired multivariate t-test, i.e. with pairwise linked samples (Westerhuis et al, 2010).

In practice, multilevel modeling is not a separate modeling technique, but rather a clever data pre-processing trick to manage sample dependency: Instead of modeling the original data from two discrete time points (or two treatments) as separate observations, an effect matrix is instead calculated as: EM = X_B_ – X_A_ (or EM = log(X_B_ / X_A_)) which in turn is modeled by regression using a dummy variable for the Y target variable. In the MUVR2 package, multilevel analysis is invoked by setting the ML argument to TRUE. The user must manually perform pre-processing to generate and supply the effect matrix as predictors (**Figure 13**). The Y target variable is deliberately left out and calculated internally. Within the MUVR2 algorithm, further data pre-processing will be done to set up the multilevel analysis:

**Figure 13**. Illustration of the multilevel analysis n MUVR2. To make a multilevel analysis using MUVR2, the user must pre-process the original data into an effect matrix (EM). MUVR2 is then called using the parameters *X=EM* and *ML=TRUE*. A new predictor matrix and a dummy Y target variable will then be calculated internally within MUVR2.

In this multilevel example using MUVR2-RF, the “crisp” dataset is used, which has untargeted plasma metabolomics data from two different dietary interventions delivered to 21 subjects in a cross-over design, i.e., where each participant received both diets. There is thus a clear systematic sample dependency (by individual) advocating multilevel analysis. Typing data("crisp") will load an effect matrix (‘crispEM’) with 21 rows (one row per individual) and 1508 columns, consisting of differences in area-under-the-curve values (AUC) of metabolic features measured after two different breakfast meal interventions.

Comments and clarification of code and results have been elaborated in the regression and classification analysis, and here we will only add explanations for the parts that are different in multilevel analysis.

# Call in relevant libraries
library(doParallel) # Parallel processing
library(MUVR2) # Machine Learning modeling

# Call in the "crisp" data from the MUVR2 package
data("crisp")

# Set method parameters
nCore <- detectCores() - 1 # Number of processor threads to use
nRep <- nCore # Number of MUVR2 repetitions
nOuter <- 8 # Number of outer cross-validation segments
varRatio <- 0.75 # Proportion of variables kept per iteration
method <- 'RF' # Selected core modeling algorithm

# Set up parallel processing
cl <- makeCluster(nCore)
registerDoParallel(cl)

# Perform modeling
MLModel <- MUVR2(X = crispEM,

ML = TRUE,

nRep = nRep,

nOuter = nOuter,

varRatio = varRatio,

method = method)

# Stop parallel processing
stopCluster(cl)

# Examine model performance and output
MLModel$nVar # Number of variables for min, mid and max models
# min mid max
# 5 6 7

MLModel$miss # Misclassified observations
# min mid max
# 8 8 8

MLModel$ber

# min mid max

# 0.1904762 0.1904762 0.1904762

MLModel$fitMetric # Fitness metrics for min, mid and max models dummy regressions
# $R2

# min mid max

# 0.7382263 0.7560964 0.7495876

# $Q2

# min mid max

# 0.4265355 0.3909502 0.3860555

MLModel$yPred # Multilevel predictions from min, mid and max models
# min mid max

# 1_ID1 -0.5804444 -0.5025397 -0.55692063

# 2_ID2 -0.7255238 -0.6231111 -0.63400000

# 3_ID3 -0.1409365 -0.1821270 -0.22317460

# 4_ID4 -0.5986032 -0.7677460 -0.72241270
# … … … …

MLModel$yClass # Predicted class from min, mid and max models
# min mid max
# 1_ID1 -1 -1 -1
# 2_ID2 -1 -1 -1
# 3_ID3 -1 -1 -1
# 4_ID4 -1 -1 -1
# … … … …

plotVAL(MLModel)


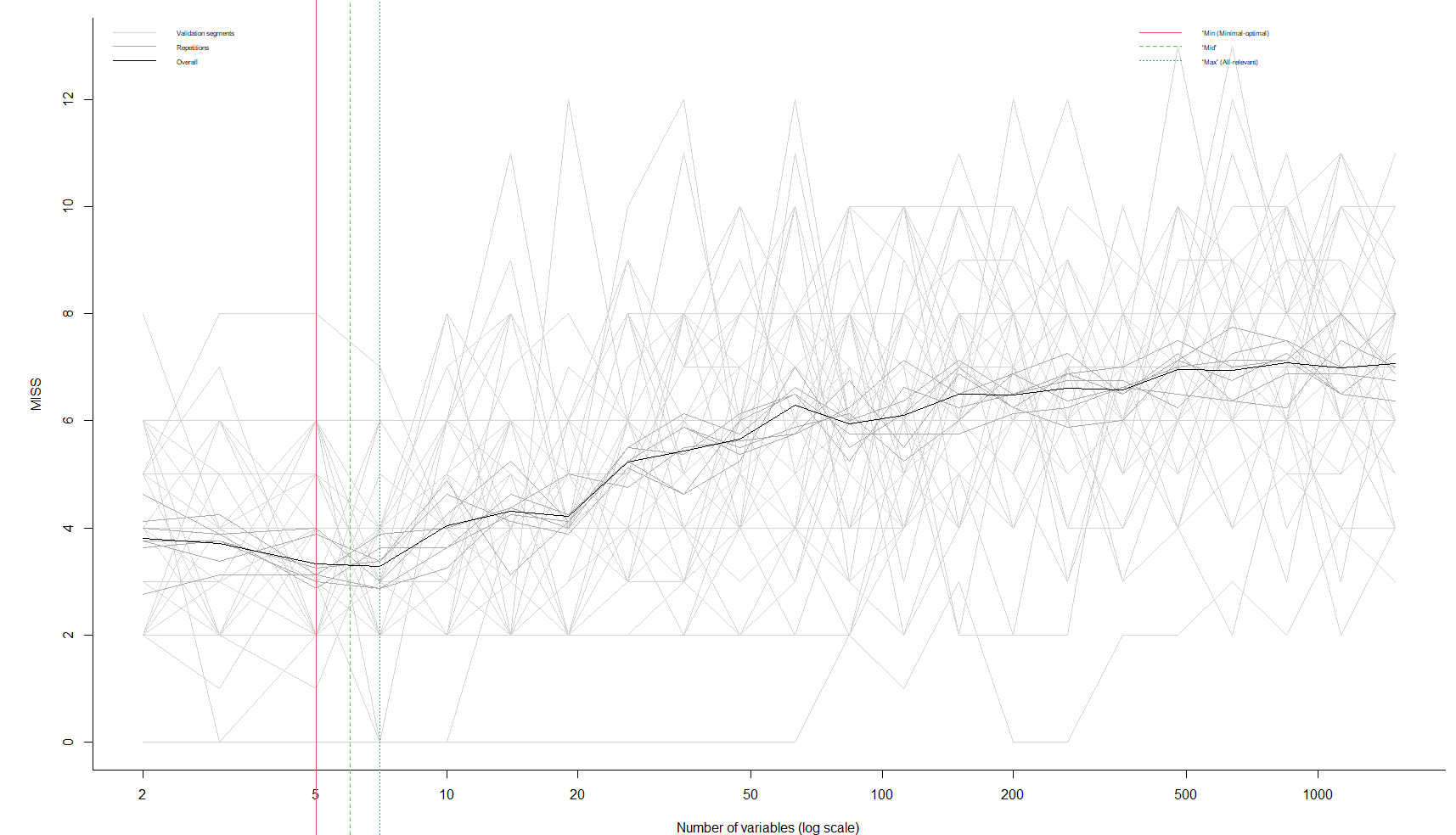


**Figure 14**. Validation plots of MUVR2-RF models in the multilevel analysis, plotted by *plotVAL()*.

The validation plot from *plotVAL()* (**Figure 14**) shows a similar behaviour as for regression. The multilevel prediction plot from *plotMV()* (**Figure 15**) is different from both the regression line plot and the classification swim lane plot. The upper and lower half of the prediction plot represent the predicted Y target variable for the positive and negative half of the effect matrix, respectively. The expected Y values are the dummy values -1 for the upper half and +1 for the lower half and predictions have a decision boundary at Y = 0. Consequently, 4 out of 21 individuals are misclassified. The MLModel$miss (number of miss classifications) will however report 8 misses since the value is based on two instances per individual (i.e. upper and lower half).

plotMV(MLModel, model = 'min') # Look at the model of choice: min, mid or max

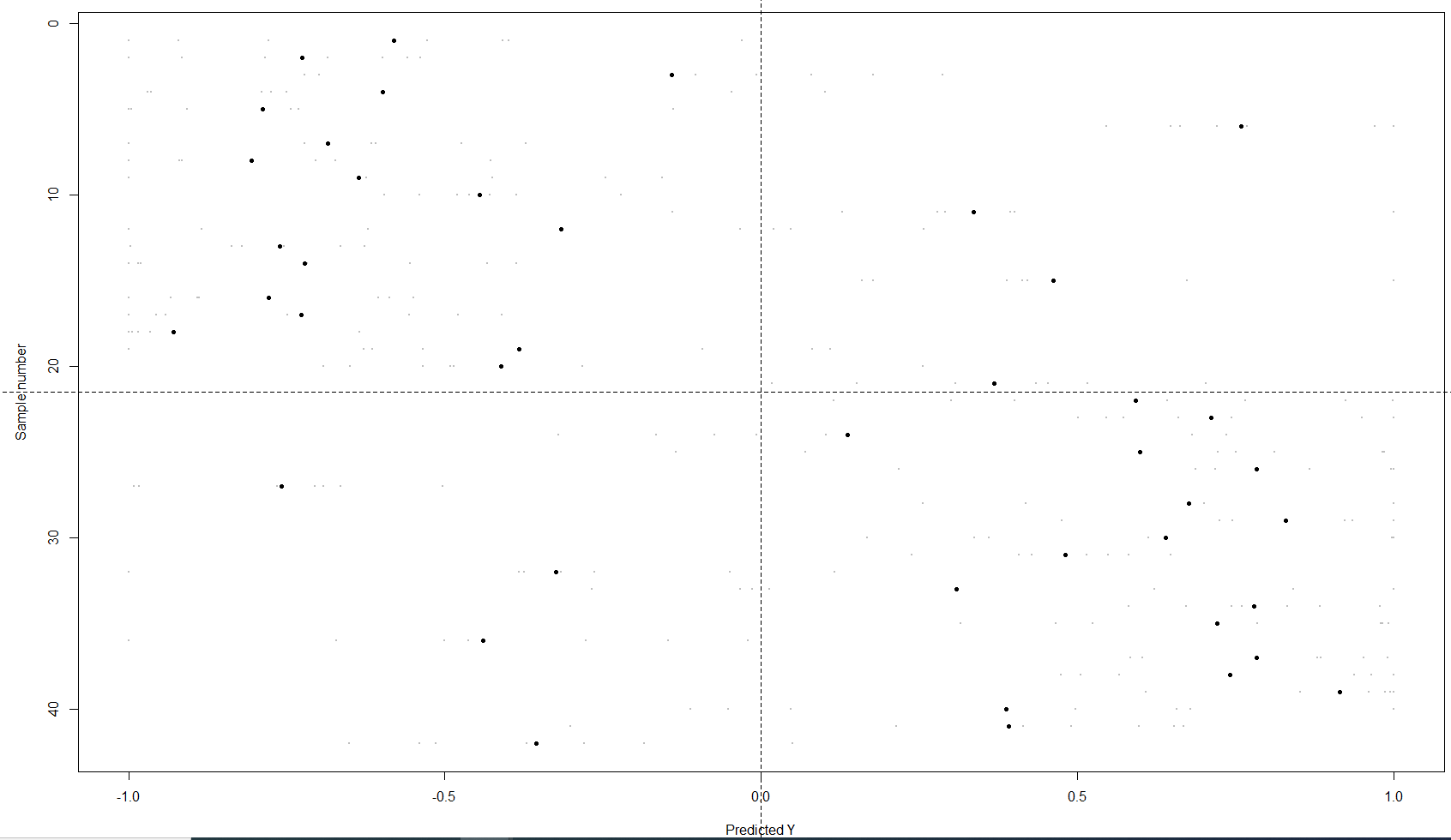


**Figure 15**. Comparison between the expected and predicted target variable of MUVR2-RF in the multilevel analysis, plotted by *plotMV()*

The stability plot from *plotStability()* (**Figure 16**) is similar to both regression and classification and will provide Q^2^ (for the dummy regression), number of misclassification and BER both per repetition and cumulative.

plotStability(MLModel, model = 'min')


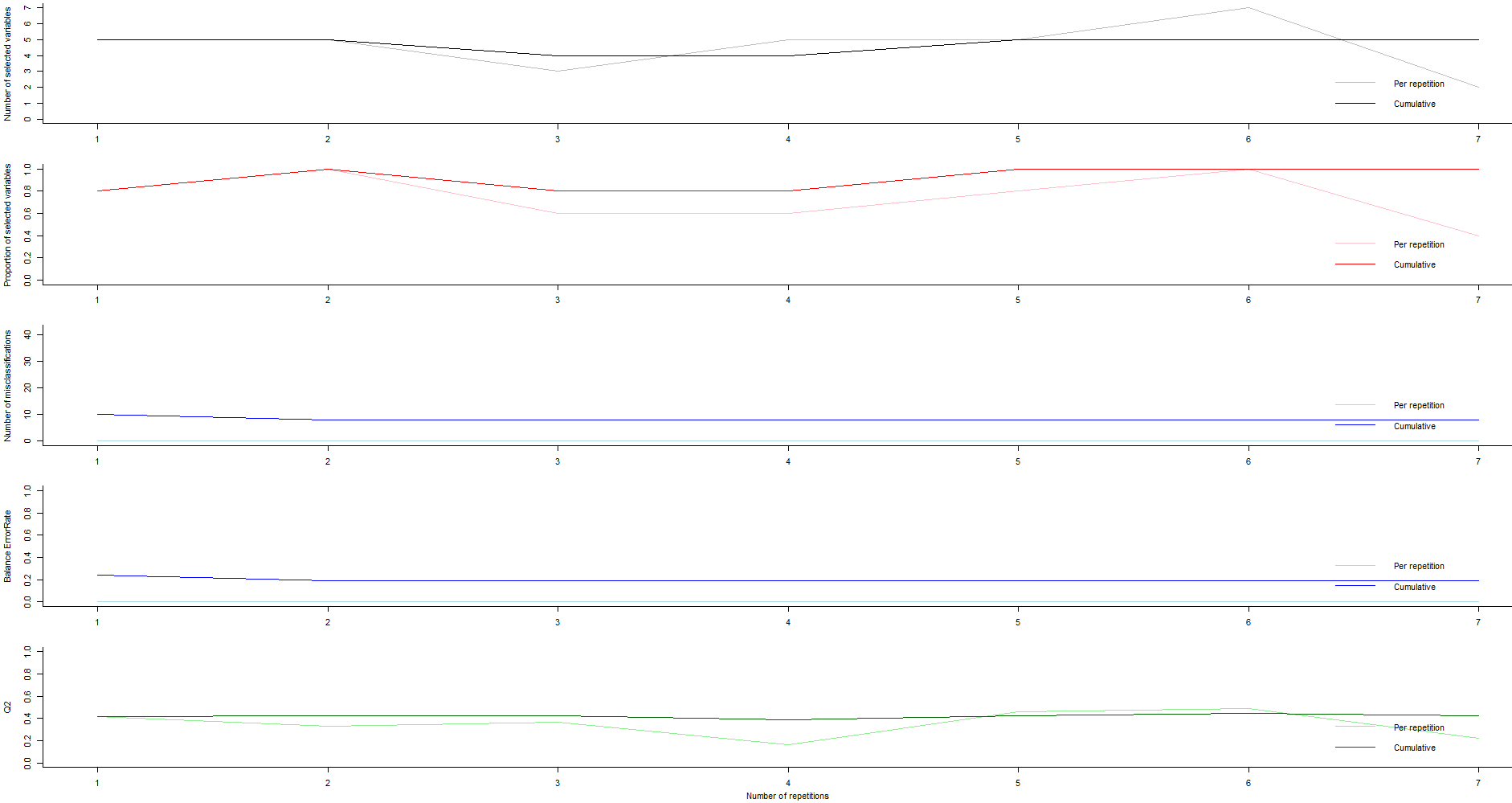


**Figure 16**. Stability plots for the minimal-optimal (‘min’) MUVR2-RF model in the multilevel analysis, plotted by *plotStability().*

The *plotVIRank()* function generates a similar boxplot (**Figure 17**) as MUVR2-PLS in section 4.1 and the getVIRank() function generates similar output as MUVR2-PLS in section 4.1 and MUVR2-EN in section 4.2

plotVIRank(MLModel, model = 'min')


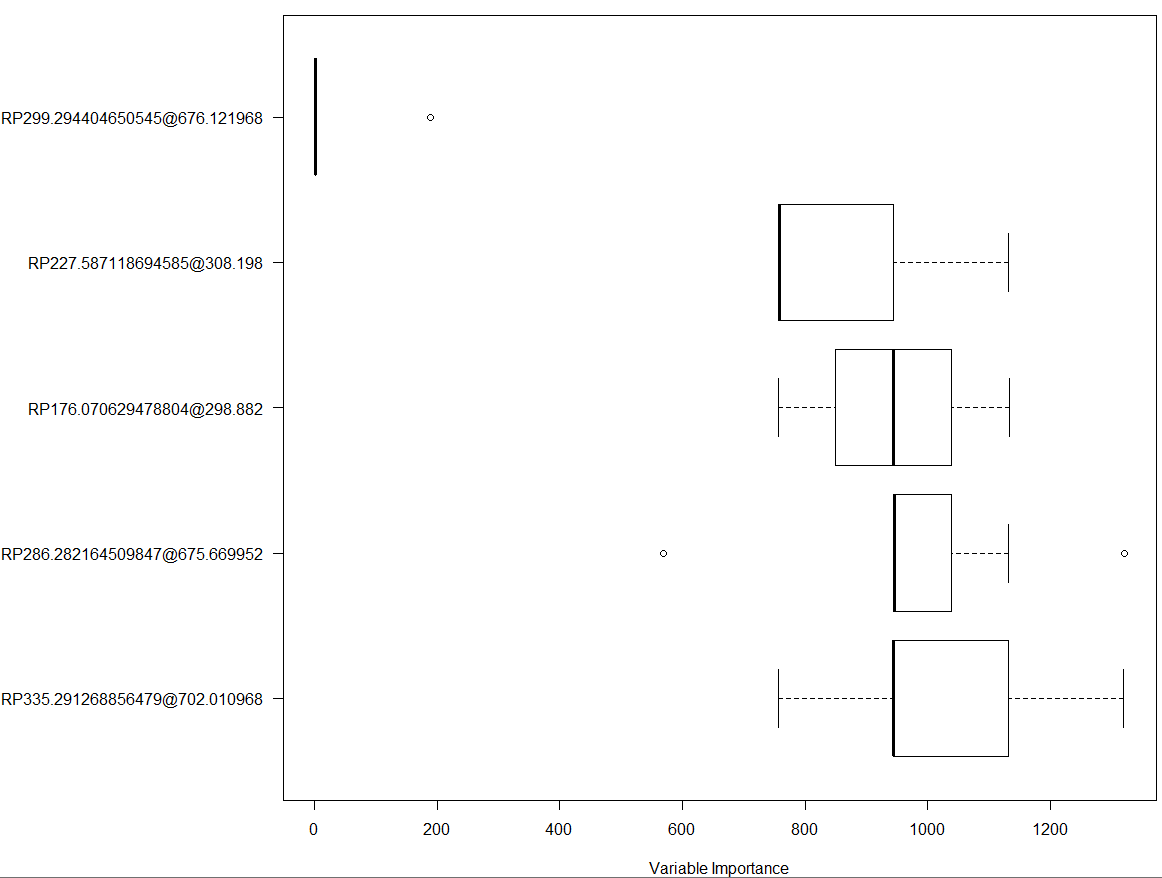


**Figure 17**. Variable importance ranks under the minimal-optimal (‘min’) MUVR2-RF model in the multilevel analysis, plotted by *plotVIRank()*

getVIRank(MLModel, model = 'min') # Extract most informative variables: Lower rank is better
# order name rank

# RP299.294404650545@676.121968 1 RP299.294404650545@676.121968 28.24362

# RP227.587118694585@308.198 2 RP227.587118694585@308.198 863.95408

# RP176.070629478804@298.882 3 RP176.070629478804@298.882 944.25000

# RP286.282164509847@675.669952 4 RP286.282164509847@675.669952 971.42092

# RP335.291268856479@702.010968 5 RP335.291268856479@702.010968 1024.52551

# 4.5. Recap

The MUVR2 algorithm provides PLS, RF and EN core machine learning methods. To avoid any confusion, ***any of the three methods can be used for regression, classification or multilevel analysis***. Moreover, all models are equally capable of addressing repeated measures (i.e. segment sampling by *ID* rather than observations) using the *ID* argument. Importantly, MUVR2-PLS and MUVR2-RF (using the *MUVR2()* function) perform variable selection by recursive variable elimination while building the models, whereas MUVR2-EN selects variables based on how often they were selected (i.e., had non-zero beta coefficients) across the *nRep x nOuter* calibrations set models. Consequently, MUVR2-PLS and MUVR2-RF gives predictions and fitness for ‘min’, ‘mid’ and ‘max’ respectively and MUVR2-EN gives only one prediction and fitness (although it still gives ‘min’, ‘mid‘ and ‘max’ variable selections). Moreover, the validation plots generated by *plotVAL()* and the plot for variable ranks by *plotVIRank()* will be similar for MUVR2-PLS and MUVR2-RF regardless of problem type, whereas *plotVAL()* on MUVR2-EN will give validation plots based on either smoothed validation fitness curves or quantiles. Additionally, the final variable selection in MUVR2-EN will use the *getVar()* function. *plotVIRank()* on MUVR2-EN will give a simple heat map that shows which variables are selected in which calibration set models.

# 5. Covariate adjustment

In the upgraded MUVR2 package, MUVR2-EN provides covariate adjustment by suppressing regularization. Importantly, similar covariate adjustment is not available in MUVR2-PLS or MUVR2-RF. In practice, to achieve such adjustment, users can quite simply provide the argument *keep = c(“names of the variables”)*. Note that the variable names need to correspond exactly to column names in the predictor (X) matrix. After covariate adjustment, the ranks of the variables that are associated with both the target variable and the covariate will decrease. An example based on section 4.2 in this tutorial is given below.

In that example, using MUVR2-EN to associate mosquitoes’ microbiota profiles to village of capture (*nRep = 35, nOuter = 8*), we can see how many times a variable had non-zero beta coefficient across *nRep x nOuter* calibration set models using classModel$varTable. Using getVIRank(classModel), we can also examine the ranks.

getVIRank(classModel, model = 'max')

# order name rank

# OUT_28 1 OUT_28 1.0

# OTU_4133 2 OTU_4133 2.0

# OUT_400 3 OUT_400 3.0

# OTU_1620 4 OTU_1620 4.0

# … … … …

# OTU_1175 76 OTU_1175 76.5

# OTU_1970 77 OTU_1970 76.5

# OUT_100 78 OUT_100 78.0

# OTU_5643 79 OTU_5643 79.0

To exemplify the use of the *keep* argument (albeit not grounded in an actual biological research question), we will artificially select one of the predictor variables as a covariate and then observe how the rank of other predictor variables that correlate with the chosen covariate change. Let’s first select a covariate to adjust for. We emphasize that this would normally be selected based on a hypothesis of the underlying causal structure. However, in this case we don’t have such covariates at hand and we instead choose one among the predictors to represent a potential covariate.

In this example, we have selected the predictor variable, “OUT_100”, that is within the ‘max’ variable selection (by quantile) in MUVR2-EN, but not within the top-ranked predictors (see rank list above). In fact, covariates should not be too strong predictors of the target variable, which would lead to a Hauck-Donner effect (Yee, 2022) and in practice throw an error from the algorithm.

We can then use the cor() function to identify which predictors among the ‘max’ predictors are strongly correlated with “OUT_100”. Using cor.test(), we can also get a p-value for the correlation. Through this procedure, we learn that the predictor “OUT_1962” correlates strongly (r = 0.83, p < 0.001) with the covariate.

# Extract the ‘max’ predictors

max_predictors <- Xotu[ , classModel$Var$max]

# Calculate the correlations between the ‘max’ predictors and "OUT_100"

max_predictor_cor <- cor(max_predictors)[ , "OTU_100"]

# Extract the name of the predictors that are strongly correlated (|r| > 0.75) with "OUT_100".

max_predictor_cor[abs(max_predictor_cor) > 0.75]

# "OTU_1962" "OTU_100"

# 0.8282541 1.0000000

# Extract the p value for the correlation

cor.test(Xotu[ , "OTU_100"], Xotu[ , "OTU_1962"])$p.value

# 2.923568e-08

We can now make a new model using "OTU_100" as a covariate (It may take some time to run):

nCore <- detectCores() - 1 # Number of processor threads to use

cl <- makeCluster(nCore)
registerDoParallel(cl)

classModel2 <- MUVR2_EN(X = Xotu,

Y = Yotu,

keep = c("OTU_100"),

nRep = 35,

nOuter = 8,

DA = TRUE)

stopCluster(cl)

Now we can observe that the variable importance rank of “OTU_1962” drops from number 20 without covariate adjustment to 703 with covariate adjustment. Across the 280 (*nRep x nOuter*) calibration set models it was selected 0 times with adjustment, comparing to 150 times without adjustment. However, please note that actual numbers of ranks and selections may fluctuate between repetitions due to stochastic effects.

# 6. Resampling tests

Permutation tests can be used to construct formal hypothesis tests and get a p-value of the actual analysis (Lindgren, et al, 1996). In brief, permutation tests are conducted by randomly sampling the target variable without replacement (permutation) and then modeling the permuted target variable using the original predictors. This modeling of random responses is repeated a number of times and statistical significance is obtained by comparing the actual modeling result to the permutation distribution, representing the null-hypothesis distribution.

In our example here, we have upgraded the permutation tests to resampling tests. In the simulation of a null-hypothesis target variable, effectively representing (pseudo-)random conditions, we have added additional variability compared to standard permutations. The rationale comes from the observation that model predictions are not bounded by the exact values or proportions of the actual target variable. We argue that the null-hypothesis target variable should similarly not have to be an exact reproduction of the actual target variable in random order. Thus, instead of obtaining the permuted target variable from sampling without replacement, we obtain the resampled target variable using random draws from its empirical distribution. In regression, sampling from the empirical distribution will thus result in numeric values not necessarily observed in the actual target variable but representing the same underlying distribution. In classification, this represents sampling the target variable based on class probabilities (i.e. sampling the actual target variable with replacement). Consequently, the null-hypothesis fitness distribution no longer exactly represents permutations of the actual target variable and, due to the sampling from empirical distributions, we refer to this new type of test as resampling tests instead of permutation tests. Resampled target variables can be generated using the *H0_test()* function and specifying the argument *type = ”resampling”.*

Here, we will show a resampling test of a random forest classification analysis of the “mosquito” data.

We first generate the actual modeling fitness using the actual target variable.

# Call in relevant libraries

data("mosquito")

# Declare modeling parameters
nCore <- detectCores() - 1
nRep <- 2 * nCore # Number of repetitions per actual model and resamplings
nOuter <- 5 # Number of validation segments
varRatio <- 0.75 # Proportion of variables to keep per iteration during variable selection
method <- 'RF' # Core modeling technique
model <- 1 # 1 for min, 2 for mid and 3 for max

# Compute actual model and extract fitness metric; Approx 0.5 min
cl <- makeCluster(nCore)
registerDoParallel(cl)
MUVR2_actual <- MUVR2(X = Xotu,

Y = Yotu,

nRep = nRep,

nOuter = nOuter,

varRatio = varRatio,

method = method)

actualFit <- MUVR2_actual$ber[model]

stopCluster(cl)

actualFit

# min

# 0.305303

Then we obtain the resampled modeling fitness distribution using the resampled target variable. To reduce computation time in this example, we set the number of resamplings to 25. The other model parameter settings should ideally be identical between the actual model and the resampled models. which is the default when performing resampling tests by the *H0_test()* function. However, to decrease the computational cost, compromises sometimes need to be made and we frequently reduce the *nRep*, *nOuter* and *varRatio* parameter settings (effectively resulting in a broader null-hypothesis distribution and larger p-values, thus “erring on the side of caution”). This can easily be achieved by changing those arguments in the call to the *H0_test()* function.

nResample <- 25 # Number of resamplings (here set to 25 for illustration; normally set to ≥100)

cl <- makeCluster(nCore)
registerDoParallel(cl)

resamplings <- H0_test(MUVR2_actual, n = nResample, type = 'resampling')

stopCluster(cl)

resampleFit <- resamplings[ , model]

resampleFit # the resampled fitness

# permutation 1 permutation 2 permutation 3 permutation 4 permutation 5 permutation 6 permutation 7

# 0.8128205 0.7285714 0.7016687 0.6840909 0.5833333 0.6111111 0.7017544

# permutation 8 permutation 9 permutation 10 permutation 11 permutation 12 permutation 13

# 0.7746032 0.7350427 0.5648148 0.7070707 0.5692308 0.5288462

# permutation 14 permutation 15 permutation 16 permutation 17 permutation 18 permutation 19

# 0.6102564 0.7449495 0.6462121 0.6433566 0.6787879 0.6259259

# permutation 20 permutation 21 permutation 22 permutation 23 permutation 24 permutation 25

# 0.6222222 0.8141026 0.8641026 0.6666667 0.4962121 0.5833333

We offer 3 different types of statistical tests to compare the actual modeling fitness to the resampled fitness distribution and to calculate a p-value: i) An empirical test, where p-values are calculated from the smoothed resampled fitness distribution as the cumulative probability. ii) A t-test, which assumes that the resampled fitness distribution follows a t-distribution and calculates the cumulative probability. iii) A non-parametric test, which uses the rank order of the actual fitness and the resampled fitness distribution, as performed by Szymańska *et al* (Szymańska *et al.*, 2012). In general, we recommend the empirical test since the resampled fitness distribution frequently does not have a Gaussian distribution and the rank-order-based non-parametric test is limited in resolution and therefore unable to assess p < (1 / *nResample*). Simulations showed that the empirical distribution-based approach generated p-values similar to those obtained from a t-distribution when the distribution was Gaussian (data not shown) and was also able to generate p-value estimates from non-Gaussian distributions that better correspond to intuitive assessment compared both to the rank order-based and t-distribution-based approaches.

The option of statistical tests can be specified by the *type* argument (*type=’smooth’* for empirical test; *type=’t’* for t-test; *type=’rank’* for rank-order based non-parametric test) in the *plotPerm()* function, which gives a visual illustration of the actual fitness, the resampled fitness distribution and the p-value. We can generate plots using the different statistical tests by the code below (**Figure 18A-C**).

# Empirical test based on smoothed curve from resampled fitness distribution (**Figure 18A**):
plotPerm(actual = actualFit, distribution = resampleFit, type = 'smooth', curve = TRUE, xlim = c(0, 1))

# Parametric resampling test significance based on student’s t test (**Figure 18B**)
plotPerm(actual = actualFit, distribution = resampleFit, type = 't', curve = TRUE, xlim = c(0, 1))

# Non-parametric resampling test significance based on the rank order of fitness values (**Figure 18C**):
plotPerm(actual = actualFit, distribution = resampleFit, type = 'rank', xlim = c(0, 1))

However, one needs to be cautious that sometimes not only the modeling using the actual target variable can be driven by overfitting: the modeling itself may be effectively hindered depending on the suitability between the machine learning method and the resampled (or permuted) data, systematically affecting the null-hypothesis distribution. We can therefore visualize the expected modeling fitness under null-hypothesis conditions using a reference distribution (**Figure 18D**), along with the actual fitness and the resampled fitness distribution (**Figure 18A-C**). This reference distribution is obtained by calculating the fitness directly from the resampled target variables, i.e., without any modeling or predictions, thereby effectively excluding any overfitting from this scenario. This can be achieved by using the *H0_reference()* function, using the target variable (*Y*), the number of resamplings (*n*) and desired performance metric (*fitness*) as arguments.

H0_reference(Y = Yotu, n = 1000, fitness = "BER")

If the resampled fitness distribution (‘resampleFit’ in the example above) resembles the reference distribution (i.e. the expected null-hypothesis distribution; ‘H0_reference’ in the example) as in **Figure 18**, it suggests that the modeling strategy does not contribute to overfitting. In this case, the estimated p-value should convincingly reflect a model’s prediction performance. On the contrary, if there is a systematic shift between the reference and the resampled fitness distributions, it suggests that the modeling strategy does not fit the data, likely driven by overfitting.


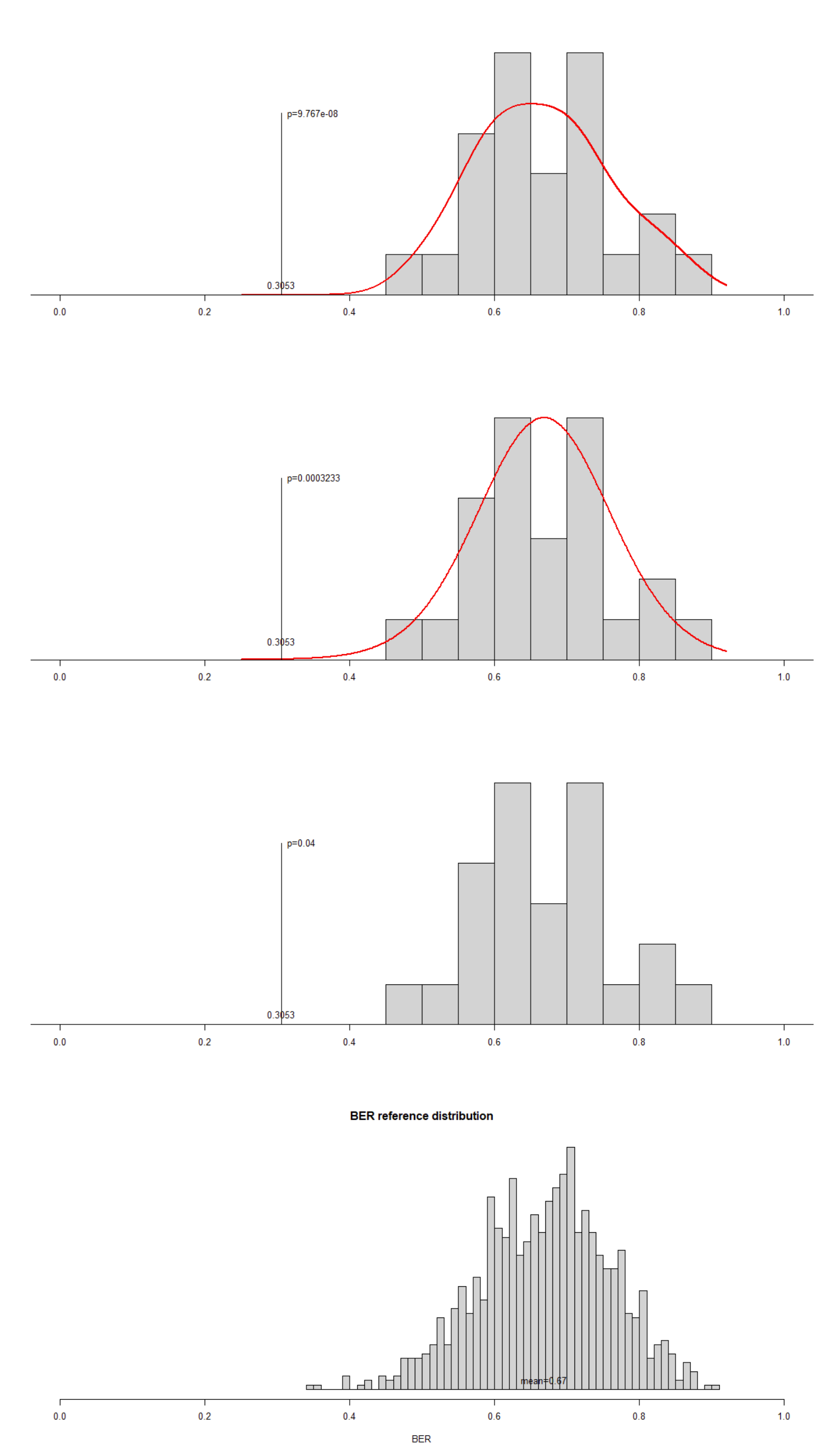


**A**
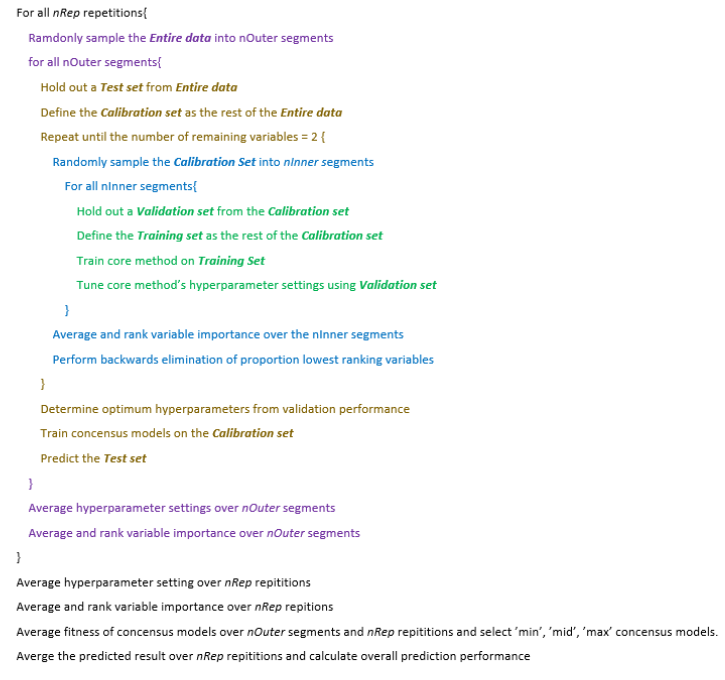


**D**
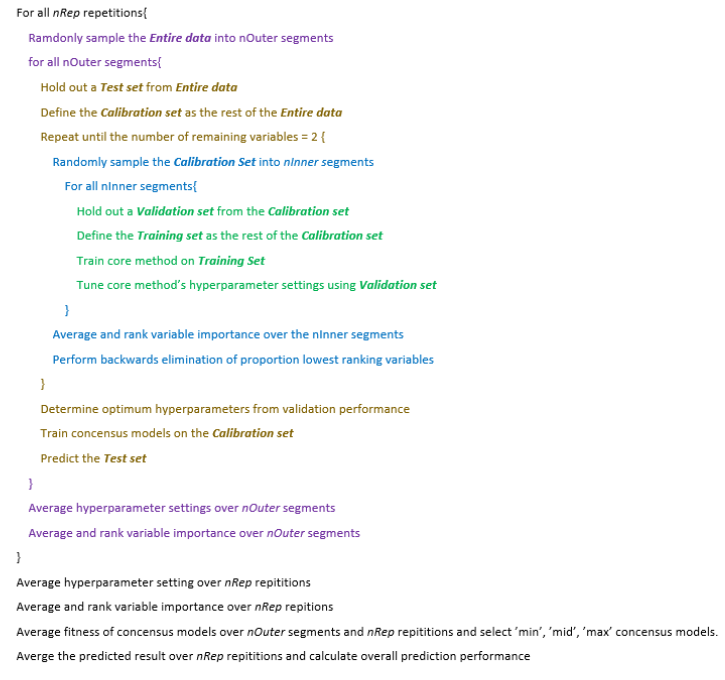


**C**
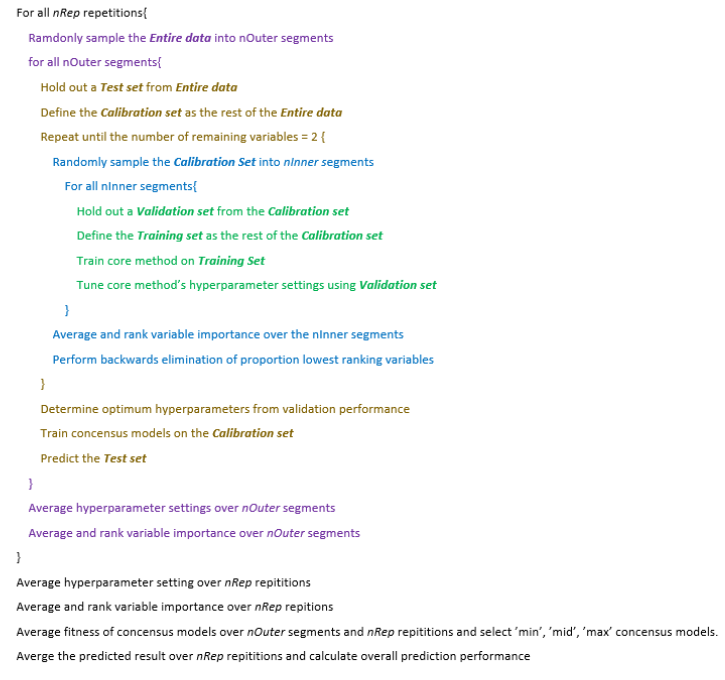


**B**
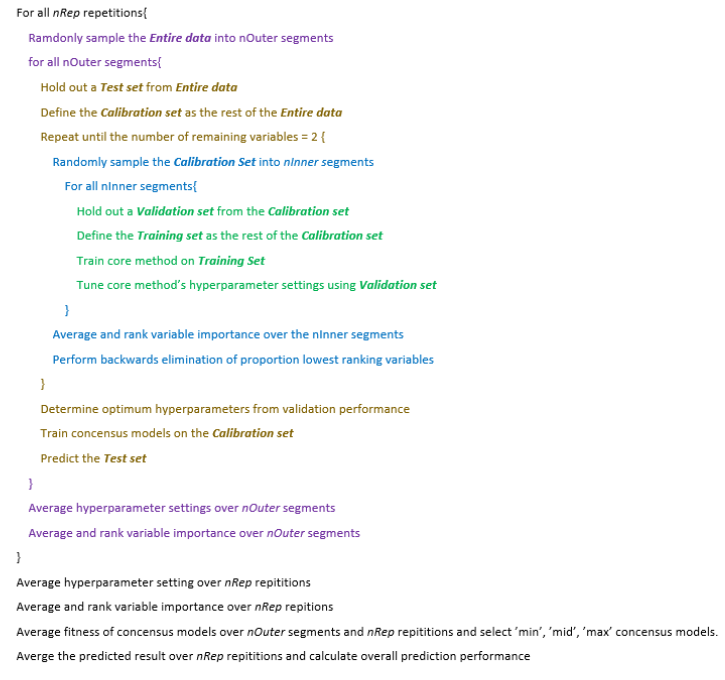


**Figure 18**. Actual finesses, resampled fitness distributions and the reference distribution **A.** The p-value is calculated as the cumulative probability based on a smoothed curve generated directly from the resampled fitness distribution. **B.** The p-value is calculated as the cumulative probability from a t-distribution approximated from the resampled fitness distribution. **C.** The p-value is calculated from the rank-order within the resampled fitness distribution (consequently with no need to calculate or visualize curves). **D.** The reference distribution is obtained by calculating the fitness directly from the resampled target variables without any modeling or predictions. BER, balanced error rate.

The above examples used BER as the fitness metric in classification analysis, but we could otherwise have used the number of misclassifications, although it is less suitable for imbalanced classes. In regression, we use Q^2^ as fitness metric. For multilevel analysis, we can use either BER, number of misclassifications or Q^2^ as fitness metrics.

Note that it is normally useful to perform initial resampling tests with few repetitions to get an indication of actual model fitness in relation to the resampled fitness distribution. Additional repetitions can then easily be added to the permutation distribution at will to obtain better p-value estimates.

# 6. Reference

Buck, M., Nilsson, L. K., Brunius, C., Dabiré, R. K., Hopkins, R., & Terenius, O. (2016). Bacterial associations reveal spatial population dynamics in Anopheles gambiae mosquitoes. Scientific Reports, 6(1), 1-9.

Hanhineva, K., Brunius, C., Andersson, A., Marklund, M., Juvonen, R., Keski‐Rahkonen, P., ... & Landberg, R. (2015). Discovery of urinary biomarkers of whole grain rye intake in free‐living subjects using nontargeted LC‐MS metabolite profiling. Molecular nutrition & food research, 59(11), 2315-2325.

Lindgren, F., Hansen, B., Karcher, W., Sjöström, M., & Eriksson, L. (1996). Model validation by permutation tests: applications to variable selection. Journal of Chemometrics, 10(5‐6), 521-532.

Shi, L., Westerhuis, J. A., Rosén, J., Landberg, R., & Brunius, C. (2019). Variable selection and validation in multivariate modelling. Bioinformatics, 35(6), 972-980.

Szymańska,E. et al. (2012) Double-check: Validation of diagnostic statistics for PLS-DA models in metabolomics studies. Metabolomics, 8, 3–16.

Westerhuis, J. A., van Velzen, E. J., Hoefsloot, H. C., & Smilde, A. K. (2010). Multivariate paired data analysis: multilevel PLSDA versus OPLSDA. Metabolomics, 6(1), 119-128.

Yee, T. W. (2022). On the Hauck–Donner effect in wald tests: Detection, tipping points, and parameter space characterization. *Journal of the American Statistical Association*, *117*(540), 1763-1774.

Yu,L. et al. (2022) Missing Data Preprocessing in Credit Classification: One-Hot Encoding or Imputation? Emerging Markets Finance and Trade, 58, 472–482.
